# Supplementary material for: Highly conserved and cis-acting lncRNAs produced from paralogous regions in the center of HOXA and HOXB clusters in the endoderm lineage
Source: PLoS Genet. 2021 Jul 19;17(7):e1009681. doi: 10.1371/journal.pgen.1009681 (PMC8330917; doi:10.1371/journal.pgen.1009681)
Supplement: S1 Dataset — (ZIP) [file pgen.1009681.s015.zip › HOXB-AS3_var1/HOXB-AS3_var1_RESULTS.html]

LncLOOM Results


# HOXB-AS3\_var1 Results

## Ulitsky Lab Weizmann Institute of Science

  

| All K-mers | Conservation by Species | Selected Species |
| --- | --- | --- |
| ▶ KMERS IN SEQUENCES | ▶ KMERS MAPPED TO ANCHOR SEQ. | ▶ DEEPEST. LEVEL SPECIFIC KMERS |
| ▶ BLOCK DIAGRAMS | ▶ BLOCK DIAGRAMS MAPPED TO ANCHOR SEQ. | ▶ DEEPEST. KMERS IN BLOCKS |
|  | ▶ MODULES |
