# Supplementary material for: Highly conserved and cis-acting lncRNAs produced from paralogous regions in the center of HOXA and HOXB clusters in the endoderm lineage
Source: PLoS Genet. 2021 Jul 19;17(7):e1009681. doi: 10.1371/journal.pgen.1009681 (PMC8330917; doi:10.1371/journal.pgen.1009681)
Supplement: S1 Dataset — (ZIP) [file pgen.1009681.s015.zip › HOXB-AS3_var1/Html_Files/Modules.html]

 MODULES


# MODULES

## start-MOTIF-end--(number of bases in between)--start-MOTIF-end

  

NAVIGATE ▼

▶HOXB\_XENOPUS (depth:5)▶HOXB\_OPOSSUM (depth:4)▶HOXB5OS (depth:3)▶HOXB\_DOG\_ISOFORM1 (depth:2)

  
  
  
  
  
  

|  |  |  |  |  |  |  |
| --- | --- | --- | --- | --- | --- | --- |
| | | | | | | | | | | | | | |
| 2 |  |  | 5 |  |  | 8 |
| Depth of motif conservation (number of species) | | | | | | |

  
  

# Modules conserved to HOXB\_XENOPUS (Depth: 5)

## Modules in Main Graph (All sequences considered):

```
>HOXB-AS3  
         1-

gtcata

gtcata  
Depth:5 (HOXB_XENOPUS)  
Ei-value:Undefined, Pi-value:Undefined  
Er-value:0.000, Pr-value:0.000  
No matches to eCLIP DataNo matches to TargetScan

-6--(0)--7-

gcgacttt

gcgacttt  
Depth:5 (HOXB_XENOPUS)  
Ei-value:Undefined, Pi-value:Undefined  
Er-value:0.000, Pr-value:0.000  
No matches to eCLIP DataNo matches to TargetScan

-14--(224)--239-

gccacc

gccacc  
Depth:5 (HOXB_XENOPUS)  
Ei-value:Undefined, Pi-value:Undefined  
Er-value:0.000, Pr-value:0.000  
No matches to eCLIP DataNo matches to TargetScan

-244--(166)--411-

taaact

taaact  
Depth:5 (HOXB_XENOPUS)  
Ei-value:Undefined, Pi-value:Undefined  
Er-value:0.000, Pr-value:0.000  
No matches to eCLIP DataNo matches to TargetScan

-416  
  
>HOXB_DOG_ISOFORM1  
        81-

gtcata

gtcata  
Depth:5 (HOXB_XENOPUS)  
Ei-value:Undefined, Pi-value:Undefined  
Er-value:0.000, Pr-value:0.000  
No matches to TargetScan

-86--(0)--87-

gcgacttt

gcgacttt  
Depth:5 (HOXB_XENOPUS)  
Ei-value:Undefined, Pi-value:Undefined  
Er-value:0.000, Pr-value:0.000  
No matches to TargetScan

-94--(227)--322-

gccacc

gccacc  
Depth:5 (HOXB_XENOPUS)  
Ei-value:Undefined, Pi-value:Undefined  
Er-value:0.000, Pr-value:0.000  
No matches to TargetScan

-327--(2309)--2637-

taaact

taaact  
Depth:5 (HOXB_XENOPUS)  
Ei-value:Undefined, Pi-value:Undefined  
Er-value:0.000, Pr-value:0.000  
No matches to TargetScan

-2642  
  
>HOXB5OS  
         3-

gtcata

gtcata  
Depth:5 (HOXB_XENOPUS)  
Ei-value:Undefined, Pi-value:Undefined  
Er-value:0.000, Pr-value:0.000  
No matches to TargetScan

-8--(0)--9-

gcgacttt

gcgacttt  
Depth:5 (HOXB_XENOPUS)  
Ei-value:Undefined, Pi-value:Undefined  
Er-value:0.000, Pr-value:0.000  
No matches to TargetScan

-16--(227)--244-

gccacc

gccacc  
Depth:5 (HOXB_XENOPUS)  
Ei-value:Undefined, Pi-value:Undefined  
Er-value:0.000, Pr-value:0.000  
No matches to TargetScan

-249--(205)--455-

taaact

taaact  
Depth:5 (HOXB_XENOPUS)  
Ei-value:Undefined, Pi-value:Undefined  
Er-value:0.000, Pr-value:0.000  
No matches to TargetScan

-460  
  
>HOXB_OPOSSUM  
       418-

gtcata

gtcata  
Depth:5 (HOXB_XENOPUS)  
Ei-value:Undefined, Pi-value:Undefined  
Er-value:0.000, Pr-value:0.000  
No matches to TargetScan

-423--(0)--424-

gcgacttt

gcgacttt  
Depth:5 (HOXB_XENOPUS)  
Ei-value:Undefined, Pi-value:Undefined  
Er-value:0.000, Pr-value:0.000  
No matches to TargetScan

-431--(526)--958-

gccacc

gccacc  
Depth:5 (HOXB_XENOPUS)  
Ei-value:Undefined, Pi-value:Undefined  
Er-value:0.000, Pr-value:0.000  
No matches to TargetScan

-963--(307)--1271-

taaact

taaact  
Depth:5 (HOXB_XENOPUS)  
Ei-value:Undefined, Pi-value:Undefined  
Er-value:0.000, Pr-value:0.000  
No matches to TargetScan

-1276  
  
>HOXB_XENOPUS  
        60-

gtcata

gtcata  
Depth:5 (HOXB_XENOPUS)  
Ei-value:Undefined, Pi-value:Undefined  
Er-value:0.000, Pr-value:0.000  
No matches to TargetScan

-65--(302)--368-

gcgacttt

gcgacttt  
Depth:5 (HOXB_XENOPUS)  
Ei-value:Undefined, Pi-value:Undefined  
Er-value:0.000, Pr-value:0.000  
No matches to TargetScan

-375--(0)--376-

gccacc

gccacc  
Depth:5 (HOXB_XENOPUS)  
Ei-value:Undefined, Pi-value:Undefined  
Er-value:0.000, Pr-value:0.000  
No matches to TargetScan

-381--(149)--531-

taaact

taaact  
Depth:5 (HOXB_XENOPUS)  
Ei-value:Undefined, Pi-value:Undefined  
Er-value:0.000, Pr-value:0.000  
No matches to TargetScan

-536
```

---

# Modules conserved to HOXB\_OPOSSUM (Depth: 4)

## Modules in Main Graph (All sequences considered):

```
>HOXB-AS3  
         1-

gtcata

gtcata  
Depth:5 (HOXB_XENOPUS)  
Ei-value:Undefined, Pi-value:Undefined  
Er-value:0.000, Pr-value:0.000  
No matches to eCLIP DataNo matches to TargetScan


gcgacttt

gcgacttt  
Depth:5 (HOXB_XENOPUS)  
Ei-value:Undefined, Pi-value:Undefined  
Er-value:0.000, Pr-value:0.000  
No matches to eCLIP DataNo matches to TargetScan


tggg

gtcatagcgacttttggg  
Depth:4 (HOXB_OPOSSUM)  
Ei-value:Undefined, Pi-value:Undefined  
Er-value:0.000, Pr-value:0.000  
No matches to eCLIP DataNo matches to TargetScan

-18--(1)--20-

tagtttgct

tagtttgct  
Depth:4 (HOXB_OPOSSUM)  
Ei-value:Undefined, Pi-value:Undefined  
Er-value:0.000, Pr-value:0.000  
No matches to eCLIP DataNo matches to TargetScan

-28--(5)--34-

caaaggg

caaaggg  
Depth:4 (HOXB_OPOSSUM)  
Ei-value:Undefined, Pi-value:Undefined  
Er-value:0.000, Pr-value:0.000  
No matches to eCLIP DataNo matches to TargetScan

-40--(73)--114-

ctcccc

ctcccc  
Depth:4 (HOXB_OPOSSUM)  
Ei-value:Undefined, Pi-value:Undefined  
Er-value:0.010, Pr-value:0.000  
No matches to eCLIP DataNo matches to TargetScan

-119--(15)--135-

gtaagaagtt

gtaagaagtt  
Depth:4 (HOXB_OPOSSUM)  
Ei-value:Undefined, Pi-value:Undefined  
Er-value:0.000, Pr-value:0.000  
No matches to eCLIP DataNo matches to TargetScan

-144--(11)--156-

gaaggga

gaaggga  
Depth:4 (HOXB_OPOSSUM)  
Ei-value:Undefined, Pi-value:Undefined  
Er-value:0.000, Pr-value:0.000  
No matches to eCLIP DataMATCHES To TargetScan▶ miR-204-5p/211-5p:UCCCUUU

-162--(27)--190-

ggcctc

ggcctc  
Depth:4 (HOXB_OPOSSUM)  
Ei-value:Undefined, Pi-value:Undefined  
Er-value:0.010, Pr-value:0.000  
No matches to eCLIP DataNo matches to TargetScan

-195--(9)--205-

tccaggc

tccaggc  
Depth:4 (HOXB_OPOSSUM)  
Ei-value:Undefined, Pi-value:Undefined  
Er-value:0.000, Pr-value:0.000  
No matches to eCLIP DataNo matches to TargetScan

-211--(27)--239-

gccacc

gccacc  
Depth:5 (HOXB_XENOPUS)  
Ei-value:Undefined, Pi-value:Undefined  
Er-value:0.000, Pr-value:0.000  
No matches to eCLIP DataNo matches to TargetScan

-244--(166)--411-

taaact

taaact  
Depth:5 (HOXB_XENOPUS)  
Ei-value:Undefined, Pi-value:Undefined  
Er-value:0.000, Pr-value:0.000  
No matches to eCLIP DataNo matches to TargetScan

-416  
  
>HOXB_DOG_ISOFORM1  
        81-

gtcata

gtcata  
Depth:5 (HOXB_XENOPUS)  
Ei-value:Undefined, Pi-value:Undefined  
Er-value:0.000, Pr-value:0.000  
No matches to TargetScan


gcgacttt

gcgacttt  
Depth:5 (HOXB_XENOPUS)  
Ei-value:Undefined, Pi-value:Undefined  
Er-value:0.000, Pr-value:0.000  
No matches to TargetScan


tggg

gtcatagcgacttttggg  
Depth:4 (HOXB_OPOSSUM)  
Ei-value:Undefined, Pi-value:Undefined  
Er-value:0.000, Pr-value:0.000  
No matches to TargetScan

-98--(1)--100-

tagtttgct

tagtttgct  
Depth:4 (HOXB_OPOSSUM)  
Ei-value:Undefined, Pi-value:Undefined  
Er-value:0.000, Pr-value:0.000  
No matches to TargetScan

-108--(5)--114-

caaaggg

caaaggg  
Depth:4 (HOXB_OPOSSUM)  
Ei-value:Undefined, Pi-value:Undefined  
Er-value:0.000, Pr-value:0.000  
No matches to TargetScan

-120--(73)--194-

ctcccc

ctcccc  
Depth:4 (HOXB_OPOSSUM)  
Ei-value:Undefined, Pi-value:Undefined  
Er-value:0.010, Pr-value:0.000  
No matches to TargetScan

-199--(15)--215-

gtaagaagtt

gtaagaagtt  
Depth:4 (HOXB_OPOSSUM)  
Ei-value:Undefined, Pi-value:Undefined  
Er-value:0.000, Pr-value:0.000  
No matches to TargetScan

-224--(11)--236-

gaaggga

gaaggga  
Depth:4 (HOXB_OPOSSUM)  
Ei-value:Undefined, Pi-value:Undefined  
Er-value:0.000, Pr-value:0.000  
MATCHES To TargetScan▶ miR-204-5p/211-5p:UCCCUUU

-242--(30)--273-

ggcctc

ggcctc  
Depth:4 (HOXB_OPOSSUM)  
Ei-value:Undefined, Pi-value:Undefined  
Er-value:0.010, Pr-value:0.000  
No matches to TargetScan

-278--(9)--288-

tccaggc

tccaggc  
Depth:4 (HOXB_OPOSSUM)  
Ei-value:Undefined, Pi-value:Undefined  
Er-value:0.000, Pr-value:0.000  
No matches to TargetScan

-294--(27)--322-

gccacc

gccacc  
Depth:5 (HOXB_XENOPUS)  
Ei-value:Undefined, Pi-value:Undefined  
Er-value:0.000, Pr-value:0.000  
No matches to TargetScan

-327--(2309)--2637-

taaact

taaact  
Depth:5 (HOXB_XENOPUS)  
Ei-value:Undefined, Pi-value:Undefined  
Er-value:0.000, Pr-value:0.000  
No matches to TargetScan

-2642  
  
>HOXB5OS  
         3-

gtcata

gtcata  
Depth:5 (HOXB_XENOPUS)  
Ei-value:Undefined, Pi-value:Undefined  
Er-value:0.000, Pr-value:0.000  
No matches to TargetScan


gcgacttt

gcgacttt  
Depth:5 (HOXB_XENOPUS)  
Ei-value:Undefined, Pi-value:Undefined  
Er-value:0.000, Pr-value:0.000  
No matches to TargetScan


tggg

gtcatagcgacttttggg  
Depth:4 (HOXB_OPOSSUM)  
Ei-value:Undefined, Pi-value:Undefined  
Er-value:0.000, Pr-value:0.000  
No matches to TargetScan

-20--(1)--22-

tagtttgct

tagtttgct  
Depth:4 (HOXB_OPOSSUM)  
Ei-value:Undefined, Pi-value:Undefined  
Er-value:0.000, Pr-value:0.000  
No matches to TargetScan

-30--(5)--36-

caaaggg

caaaggg  
Depth:4 (HOXB_OPOSSUM)  
Ei-value:Undefined, Pi-value:Undefined  
Er-value:0.000, Pr-value:0.000  
No matches to TargetScan

-42--(72)--115-

ctcccc

ctcccc  
Depth:4 (HOXB_OPOSSUM)  
Ei-value:Undefined, Pi-value:Undefined  
Er-value:0.010, Pr-value:0.000  
No matches to TargetScan

-120--(19)--140-

gtaagaagtt

gtaagaagtt  
Depth:4 (HOXB_OPOSSUM)  
Ei-value:Undefined, Pi-value:Undefined  
Er-value:0.000, Pr-value:0.000  
No matches to TargetScan

-149--(11)--161-

gaaggga

gaaggga  
Depth:4 (HOXB_OPOSSUM)  
Ei-value:Undefined, Pi-value:Undefined  
Er-value:0.000, Pr-value:0.000  
MATCHES To TargetScan▶ miR-204-5p/211-5p:UCCCUUU

-167--(27)--195-

ggcctc

ggcctc  
Depth:4 (HOXB_OPOSSUM)  
Ei-value:Undefined, Pi-value:Undefined  
Er-value:0.010, Pr-value:0.000  
No matches to TargetScan

-200--(9)--210-

tccaggc

tccaggc  
Depth:4 (HOXB_OPOSSUM)  
Ei-value:Undefined, Pi-value:Undefined  
Er-value:0.000, Pr-value:0.000  
No matches to TargetScan

-216--(27)--244-

gccacc

gccacc  
Depth:5 (HOXB_XENOPUS)  
Ei-value:Undefined, Pi-value:Undefined  
Er-value:0.000, Pr-value:0.000  
No matches to TargetScan

-249--(205)--455-

taaact

taaact  
Depth:5 (HOXB_XENOPUS)  
Ei-value:Undefined, Pi-value:Undefined  
Er-value:0.000, Pr-value:0.000  
No matches to TargetScan

-460  
  
>HOXB_OPOSSUM  
       418-

gtcata

gtcata  
Depth:5 (HOXB_XENOPUS)  
Ei-value:Undefined, Pi-value:Undefined  
Er-value:0.000, Pr-value:0.000  
No matches to TargetScan


gcgacttt

gcgacttt  
Depth:5 (HOXB_XENOPUS)  
Ei-value:Undefined, Pi-value:Undefined  
Er-value:0.000, Pr-value:0.000  
No matches to TargetScan


tggg

gtcatagcgacttttggg  
Depth:4 (HOXB_OPOSSUM)  
Ei-value:Undefined, Pi-value:Undefined  
Er-value:0.000, Pr-value:0.000  
No matches to TargetScan

-435--(1)--437-

tagtttgct

tagtttgct  
Depth:4 (HOXB_OPOSSUM)  
Ei-value:Undefined, Pi-value:Undefined  
Er-value:0.000, Pr-value:0.000  
No matches to TargetScan

-445--(35)--481-

caaaggg

caaaggg  
Depth:4 (HOXB_OPOSSUM)  
Ei-value:Undefined, Pi-value:Undefined  
Er-value:0.000, Pr-value:0.000  
No matches to TargetScan

-487--(46)--534-

ctcccc

ctcccc  
Depth:4 (HOXB_OPOSSUM)  
Ei-value:Undefined, Pi-value:Undefined  
Er-value:0.010, Pr-value:0.000  
No matches to TargetScan

-539--(17)--557-

gtaagaagtt

gtaagaagtt  
Depth:4 (HOXB_OPOSSUM)  
Ei-value:Undefined, Pi-value:Undefined  
Er-value:0.000, Pr-value:0.000  
No matches to TargetScan

-566--(7)--574-

gaaggga

gaaggga  
Depth:4 (HOXB_OPOSSUM)  
Ei-value:Undefined, Pi-value:Undefined  
Er-value:0.000, Pr-value:0.000  
MATCHES To TargetScan▶ miR-204-5p/211-5p:UCCCUUU

-580--(69)--650-

ggcctc

ggcctc  
Depth:4 (HOXB_OPOSSUM)  
Ei-value:Undefined, Pi-value:Undefined  
Er-value:0.010, Pr-value:0.000  
No matches to TargetScan

-655--(260)--916-

tccaggc

tccaggc  
Depth:4 (HOXB_OPOSSUM)  
Ei-value:Undefined, Pi-value:Undefined  
Er-value:0.000, Pr-value:0.000  
No matches to TargetScan

-922--(35)--958-

gccacc

gccacc  
Depth:5 (HOXB_XENOPUS)  
Ei-value:Undefined, Pi-value:Undefined  
Er-value:0.000, Pr-value:0.000  
No matches to TargetScan

-963--(307)--1271-

taaact

taaact  
Depth:5 (HOXB_XENOPUS)  
Ei-value:Undefined, Pi-value:Undefined  
Er-value:0.000, Pr-value:0.000  
No matches to TargetScan

-1276
```

---

# Modules conserved to HOXB5OS (Depth: 3)

## Modules in Main Graph (All sequences considered):

```
>HOXB-AS3  
         1-

gtcata

gtcata  
Depth:5 (HOXB_XENOPUS)  
Ei-value:Undefined, Pi-value:Undefined  
Er-value:0.000, Pr-value:0.000  
No matches to eCLIP DataNo matches to TargetScan


gcgacttt

gcgacttt  
Depth:5 (HOXB_XENOPUS)  
Ei-value:Undefined, Pi-value:Undefined  
Er-value:0.000, Pr-value:0.000  
No matches to eCLIP DataNo matches to TargetScan


tggg

gtcatagcgacttttggg  
Depth:4 (HOXB_OPOSSUM)  
Ei-value:Undefined, Pi-value:Undefined  
Er-value:0.000, Pr-value:0.000  
No matches to eCLIP DataNo matches to TargetScan

-18--(1)--20-

tagtttgct

tagtttgct  
Depth:4 (HOXB_OPOSSUM)  
Ei-value:Undefined, Pi-value:Undefined  
Er-value:0.000, Pr-value:0.000  
No matches to eCLIP DataNo matches to TargetScan

-28--(5)--34-

caaaggg

caaaggg  
Depth:4 (HOXB_OPOSSUM)  
Ei-value:Undefined, Pi-value:Undefined  
Er-value:0.000, Pr-value:0.000  
No matches to eCLIP DataNo matches to TargetScan

-40--(1)--42-

gacaaagtca

gacaaagtca  
Depth:3 (HOXB5OS)  
Ei-value:Undefined, Pi-value:Undefined  
Er-value:0.000, Pr-value:0.000  
No matches to eCLIP DataNo matches to TargetScan

-51--(13)--65-

aaggagg

aaggagg  
Depth:3 (HOXB5OS)  
Ei-value:Undefined, Pi-value:Undefined  
Er-value:0.000, Pr-value:0.000  
No matches to eCLIP DataNo matches to TargetScan

-71--(9)--81-

agcctc

agcctc  
Depth:3 (HOXB5OS)  
Ei-value:Undefined, Pi-value:Undefined  
Er-value:0.000, Pr-value:0.000  
No matches to eCLIP DataMATCHES To TargetScan▶ miR-485-5p:GAGGCUG

-86--(18)--105-

cctcacca

cctcacca  
Depth:3 (HOXB5OS)  
Ei-value:Undefined, Pi-value:Undefined  
Er-value:0.000, Pr-value:0.000  
No matches to eCLIP DataNo matches to TargetScan

-112--(0)--113-

g

gctcccc  
Depth:3 (HOXB5OS)  
Ei-value:Undefined, Pi-value:Undefined  
Er-value:0.000, Pr-value:0.000  
No matches to eCLIP DataNo matches to TargetScan


ctcccc

ctcccc  
Depth:4 (HOXB_OPOSSUM)  
Ei-value:Undefined, Pi-value:Undefined  
Er-value:0.010, Pr-value:0.000  
No matches to eCLIP DataNo matches to TargetScan

-119--(15)--135-

gtaagaagtt

gtaagaagtt  
Depth:4 (HOXB_OPOSSUM)  
Ei-value:Undefined, Pi-value:Undefined  
Er-value:0.000, Pr-value:0.000  
No matches to eCLIP DataNo matches to TargetScan


gggcc

gtaagaagttgggcc  
Depth:3 (HOXB5OS)  
Ei-value:Undefined, Pi-value:Undefined  
Er-value:0.000, Pr-value:0.000  
No matches to eCLIP DataNo matches to TargetScan

-149--(1)--151-

agctg

agctggaagggattgaccg  
Depth:3 (HOXB5OS)  
Ei-value:Undefined, Pi-value:Undefined  
Er-value:0.000, Pr-value:0.000  
No matches to eCLIP DataMATCHES To TargetScan▶ miR-188-5p:AUCCCUU▶ miR-204-5p/211-5p:UCCCUUU


gaaggga

gaaggga  
Depth:4 (HOXB_OPOSSUM)  
Ei-value:Undefined, Pi-value:Undefined  
Er-value:0.000, Pr-value:0.000  
No matches to eCLIP DataMATCHES To TargetScan▶ miR-204-5p/211-5p:UCCCUUU


ttgaccg

agctggaagggattgaccg  
Depth:3 (HOXB5OS)  
Ei-value:Undefined, Pi-value:Undefined  
Er-value:0.000, Pr-value:0.000  
No matches to eCLIP DataMATCHES To TargetScan▶ miR-188-5p:AUCCCUU▶ miR-204-5p/211-5p:UCCCUUU

-169--(20)--190-

ggcctc

ggcctc  
Depth:4 (HOXB_OPOSSUM)  
Ei-value:Undefined, Pi-value:Undefined  
Er-value:0.010, Pr-value:0.000  
No matches to eCLIP DataNo matches to TargetScan

-195--(1)--197-

gcggagat

gcggagattccaggccc  
Depth:3 (HOXB5OS)  
Ei-value:Undefined, Pi-value:Undefined  
Er-value:0.000, Pr-value:0.000  
No matches to eCLIP DataMATCHES To TargetScan▶ miR-216a-5p:AAUCUCA▶ miR-216b-5p:AAUCUCU


tccaggc

tccaggc  
Depth:4 (HOXB_OPOSSUM)  
Ei-value:Undefined, Pi-value:Undefined  
Er-value:0.000, Pr-value:0.000  
No matches to eCLIP DataNo matches to TargetScan


cc

gcggagattccaggccc  
Depth:3 (HOXB5OS)  
Ei-value:Undefined, Pi-value:Undefined  
Er-value:0.000, Pr-value:0.000  
No matches to eCLIP DataMATCHES To TargetScan▶ miR-216a-5p:AAUCUCA▶ miR-216b-5p:AAUCUCU

-213--(22)--236-

agc

agcgccaccgcc  
Depth:3 (HOXB5OS)  
Ei-value:Undefined, Pi-value:Undefined  
Er-value:0.000, Pr-value:0.000  
No matches to eCLIP DataNo matches to TargetScan


gccacc

gccacc  
Depth:5 (HOXB_XENOPUS)  
Ei-value:Undefined, Pi-value:Undefined  
Er-value:0.000, Pr-value:0.000  
No matches to eCLIP DataNo matches to TargetScan


gcc

agcgccaccgcc  
Depth:3 (HOXB5OS)  
Ei-value:Undefined, Pi-value:Undefined  
Er-value:0.000, Pr-value:0.000  
No matches to eCLIP DataNo matches to TargetScan

-247--(137)--385-

gccggga

gccggga  
Depth:3 (HOXB5OS)  
Ei-value:Undefined, Pi-value:Undefined  
Er-value:0.000, Pr-value:0.000  
No matches to eCLIP DataNo matches to TargetScan

-391--(5)--397-

ccacac

ccacac  
Depth:3 (HOXB5OS)  
Ei-value:Undefined, Pi-value:Undefined  
Er-value:0.000, Pr-value:0.000  
No matches to eCLIP DataNo matches to TargetScan

-402--(6)--409-

gg

ggtaaact  
Depth:3 (HOXB5OS)  
Ei-value:Undefined, Pi-value:Undefined  
Er-value:0.000, Pr-value:0.000  
No matches to eCLIP DataNo matches to TargetScan


taaact

taaact  
Depth:5 (HOXB_XENOPUS)  
Ei-value:Undefined, Pi-value:Undefined  
Er-value:0.000, Pr-value:0.000  
No matches to eCLIP DataNo matches to TargetScan

-416--(80)--497-

aatttagaa

aatttagaa  
Depth:3 (HOXB5OS)  
Ei-value:Undefined, Pi-value:Undefined  
Er-value:0.000, Pr-value:0.000  
No matches to eCLIP DataNo matches to TargetScan

-505  
  
>HOXB_DOG_ISOFORM1  
        81-

gtcata

gtcata  
Depth:5 (HOXB_XENOPUS)  
Ei-value:Undefined, Pi-value:Undefined  
Er-value:0.000, Pr-value:0.000  
No matches to TargetScan


gcgacttt

gcgacttt  
Depth:5 (HOXB_XENOPUS)  
Ei-value:Undefined, Pi-value:Undefined  
Er-value:0.000, Pr-value:0.000  
No matches to TargetScan


tggg

gtcatagcgacttttggg  
Depth:4 (HOXB_OPOSSUM)  
Ei-value:Undefined, Pi-value:Undefined  
Er-value:0.000, Pr-value:0.000  
No matches to TargetScan

-98--(1)--100-

tagtttgct

tagtttgct  
Depth:4 (HOXB_OPOSSUM)  
Ei-value:Undefined, Pi-value:Undefined  
Er-value:0.000, Pr-value:0.000  
No matches to TargetScan

-108--(5)--114-

caaaggg

caaaggg  
Depth:4 (HOXB_OPOSSUM)  
Ei-value:Undefined, Pi-value:Undefined  
Er-value:0.000, Pr-value:0.000  
No matches to TargetScan

-120--(1)--122-

gacaaagtca

gacaaagtca  
Depth:3 (HOXB5OS)  
Ei-value:Undefined, Pi-value:Undefined  
Er-value:0.000, Pr-value:0.000  
No matches to TargetScan

-131--(13)--145-

aaggagg

aaggagg  
Depth:3 (HOXB5OS)  
Ei-value:Undefined, Pi-value:Undefined  
Er-value:0.000, Pr-value:0.000  
No matches to TargetScan

-151--(9)--161-

agcctc

agcctc  
Depth:3 (HOXB5OS)  
Ei-value:Undefined, Pi-value:Undefined  
Er-value:0.000, Pr-value:0.000  
MATCHES To TargetScan▶ miR-485-5p:GAGGCUG

-166--(18)--185-

cctcacca

cctcacca  
Depth:3 (HOXB5OS)  
Ei-value:Undefined, Pi-value:Undefined  
Er-value:0.000, Pr-value:0.000  
No matches to TargetScan

-192--(0)--193-

g

gctcccc  
Depth:3 (HOXB5OS)  
Ei-value:Undefined, Pi-value:Undefined  
Er-value:0.000, Pr-value:0.000  
No matches to TargetScan


ctcccc

ctcccc  
Depth:4 (HOXB_OPOSSUM)  
Ei-value:Undefined, Pi-value:Undefined  
Er-value:0.010, Pr-value:0.000  
No matches to TargetScan

-199--(15)--215-

gtaagaagtt

gtaagaagtt  
Depth:4 (HOXB_OPOSSUM)  
Ei-value:Undefined, Pi-value:Undefined  
Er-value:0.000, Pr-value:0.000  
No matches to TargetScan


gggcc

gtaagaagttgggcc  
Depth:3 (HOXB5OS)  
Ei-value:Undefined, Pi-value:Undefined  
Er-value:0.000, Pr-value:0.000  
No matches to TargetScan

-229--(1)--231-

agctg

agctggaagggattgaccg  
Depth:3 (HOXB5OS)  
Ei-value:Undefined, Pi-value:Undefined  
Er-value:0.000, Pr-value:0.000  
MATCHES To TargetScan▶ miR-188-5p:AUCCCUU▶ miR-204-5p/211-5p:UCCCUUU


gaaggga

gaaggga  
Depth:4 (HOXB_OPOSSUM)  
Ei-value:Undefined, Pi-value:Undefined  
Er-value:0.000, Pr-value:0.000  
MATCHES To TargetScan▶ miR-204-5p/211-5p:UCCCUUU


ttgaccg

agctggaagggattgaccg  
Depth:3 (HOXB5OS)  
Ei-value:Undefined, Pi-value:Undefined  
Er-value:0.000, Pr-value:0.000  
MATCHES To TargetScan▶ miR-188-5p:AUCCCUU▶ miR-204-5p/211-5p:UCCCUUU

-249--(23)--273-

ggcctc

ggcctc  
Depth:4 (HOXB_OPOSSUM)  
Ei-value:Undefined, Pi-value:Undefined  
Er-value:0.010, Pr-value:0.000  
No matches to TargetScan

-278--(1)--280-

gcggagat

gcggagattccaggccc  
Depth:3 (HOXB5OS)  
Ei-value:Undefined, Pi-value:Undefined  
Er-value:0.000, Pr-value:0.000  
MATCHES To TargetScan▶ miR-216a-5p:AAUCUCA▶ miR-216b-5p:AAUCUCU


tccaggc

tccaggc  
Depth:4 (HOXB_OPOSSUM)  
Ei-value:Undefined, Pi-value:Undefined  
Er-value:0.000, Pr-value:0.000  
No matches to TargetScan


cc

gcggagattccaggccc  
Depth:3 (HOXB5OS)  
Ei-value:Undefined, Pi-value:Undefined  
Er-value:0.000, Pr-value:0.000  
MATCHES To TargetScan▶ miR-216a-5p:AAUCUCA▶ miR-216b-5p:AAUCUCU

-296--(22)--319-

agc

agcgccaccgcc  
Depth:3 (HOXB5OS)  
Ei-value:Undefined, Pi-value:Undefined  
Er-value:0.000, Pr-value:0.000  
No matches to TargetScan


gccacc

gccacc  
Depth:5 (HOXB_XENOPUS)  
Ei-value:Undefined, Pi-value:Undefined  
Er-value:0.000, Pr-value:0.000  
No matches to TargetScan


gcc

agcgccaccgcc  
Depth:3 (HOXB5OS)  
Ei-value:Undefined, Pi-value:Undefined  
Er-value:0.000, Pr-value:0.000  
No matches to TargetScan

-330--(2280)--2611-

gccggga

gccggga  
Depth:3 (HOXB5OS)  
Ei-value:Undefined, Pi-value:Undefined  
Er-value:0.000, Pr-value:0.000  
No matches to TargetScan

-2617--(5)--2623-

ccacac

ccacac  
Depth:3 (HOXB5OS)  
Ei-value:Undefined, Pi-value:Undefined  
Er-value:0.000, Pr-value:0.000  
No matches to TargetScan

-2628--(6)--2635-

gg

ggtaaact  
Depth:3 (HOXB5OS)  
Ei-value:Undefined, Pi-value:Undefined  
Er-value:0.000, Pr-value:0.000  
No matches to TargetScan


taaact

taaact  
Depth:5 (HOXB_XENOPUS)  
Ei-value:Undefined, Pi-value:Undefined  
Er-value:0.000, Pr-value:0.000  
No matches to TargetScan

-2642--(81)--2724-

aatttagaa

aatttagaa  
Depth:3 (HOXB5OS)  
Ei-value:Undefined, Pi-value:Undefined  
Er-value:0.000, Pr-value:0.000  
No matches to TargetScan

-2732  
  
>HOXB5OS  
         3-

gtcata

gtcata  
Depth:5 (HOXB_XENOPUS)  
Ei-value:Undefined, Pi-value:Undefined  
Er-value:0.000, Pr-value:0.000  
No matches to TargetScan


gcgacttt

gcgacttt  
Depth:5 (HOXB_XENOPUS)  
Ei-value:Undefined, Pi-value:Undefined  
Er-value:0.000, Pr-value:0.000  
No matches to TargetScan


tggg

gtcatagcgacttttggg  
Depth:4 (HOXB_OPOSSUM)  
Ei-value:Undefined, Pi-value:Undefined  
Er-value:0.000, Pr-value:0.000  
No matches to TargetScan

-20--(1)--22-

tagtttgct

tagtttgct  
Depth:4 (HOXB_OPOSSUM)  
Ei-value:Undefined, Pi-value:Undefined  
Er-value:0.000, Pr-value:0.000  
No matches to TargetScan

-30--(5)--36-

caaaggg

caaaggg  
Depth:4 (HOXB_OPOSSUM)  
Ei-value:Undefined, Pi-value:Undefined  
Er-value:0.000, Pr-value:0.000  
No matches to TargetScan

-42--(1)--44-

gacaaagtca

gacaaagtca  
Depth:3 (HOXB5OS)  
Ei-value:Undefined, Pi-value:Undefined  
Er-value:0.000, Pr-value:0.000  
No matches to TargetScan

-53--(13)--67-

aaggagg

aaggagg  
Depth:3 (HOXB5OS)  
Ei-value:Undefined, Pi-value:Undefined  
Er-value:0.000, Pr-value:0.000  
No matches to TargetScan

-73--(9)--83-

agcctc

agcctc  
Depth:3 (HOXB5OS)  
Ei-value:Undefined, Pi-value:Undefined  
Er-value:0.000, Pr-value:0.000  
MATCHES To TargetScan▶ miR-485-5p:GAGGCUG

-88--(6)--95-

cctcacca

cctcacca  
Depth:3 (HOXB5OS)  
Ei-value:Undefined, Pi-value:Undefined  
Er-value:0.000, Pr-value:0.000  
No matches to TargetScan

-102--(11)--114-

g

gctcccc  
Depth:3 (HOXB5OS)  
Ei-value:Undefined, Pi-value:Undefined  
Er-value:0.000, Pr-value:0.000  
No matches to TargetScan


ctcccc

ctcccc  
Depth:4 (HOXB_OPOSSUM)  
Ei-value:Undefined, Pi-value:Undefined  
Er-value:0.010, Pr-value:0.000  
No matches to TargetScan

-120--(19)--140-

gtaagaagtt

gtaagaagtt  
Depth:4 (HOXB_OPOSSUM)  
Ei-value:Undefined, Pi-value:Undefined  
Er-value:0.000, Pr-value:0.000  
No matches to TargetScan


gggcc

gtaagaagttgggcc  
Depth:3 (HOXB5OS)  
Ei-value:Undefined, Pi-value:Undefined  
Er-value:0.000, Pr-value:0.000  
No matches to TargetScan

-154--(1)--156-

agctg

agctggaagggattgaccg  
Depth:3 (HOXB5OS)  
Ei-value:Undefined, Pi-value:Undefined  
Er-value:0.000, Pr-value:0.000  
MATCHES To TargetScan▶ miR-188-5p:AUCCCUU▶ miR-204-5p/211-5p:UCCCUUU


gaaggga

gaaggga  
Depth:4 (HOXB_OPOSSUM)  
Ei-value:Undefined, Pi-value:Undefined  
Er-value:0.000, Pr-value:0.000  
MATCHES To TargetScan▶ miR-204-5p/211-5p:UCCCUUU


ttgaccg

agctggaagggattgaccg  
Depth:3 (HOXB5OS)  
Ei-value:Undefined, Pi-value:Undefined  
Er-value:0.000, Pr-value:0.000  
MATCHES To TargetScan▶ miR-188-5p:AUCCCUU▶ miR-204-5p/211-5p:UCCCUUU

-174--(20)--195-

ggcctc

ggcctc  
Depth:4 (HOXB_OPOSSUM)  
Ei-value:Undefined, Pi-value:Undefined  
Er-value:0.010, Pr-value:0.000  
No matches to TargetScan

-200--(1)--202-

gcggagat

gcggagattccaggccc  
Depth:3 (HOXB5OS)  
Ei-value:Undefined, Pi-value:Undefined  
Er-value:0.000, Pr-value:0.000  
MATCHES To TargetScan▶ miR-216a-5p:AAUCUCA▶ miR-216b-5p:AAUCUCU


tccaggc

tccaggc  
Depth:4 (HOXB_OPOSSUM)  
Ei-value:Undefined, Pi-value:Undefined  
Er-value:0.000, Pr-value:0.000  
No matches to TargetScan


cc

gcggagattccaggccc  
Depth:3 (HOXB5OS)  
Ei-value:Undefined, Pi-value:Undefined  
Er-value:0.000, Pr-value:0.000  
MATCHES To TargetScan▶ miR-216a-5p:AAUCUCA▶ miR-216b-5p:AAUCUCU

-218--(22)--241-

agc

agcgccaccgcc  
Depth:3 (HOXB5OS)  
Ei-value:Undefined, Pi-value:Undefined  
Er-value:0.000, Pr-value:0.000  
No matches to TargetScan


gccacc

gccacc  
Depth:5 (HOXB_XENOPUS)  
Ei-value:Undefined, Pi-value:Undefined  
Er-value:0.000, Pr-value:0.000  
No matches to TargetScan


gcc

agcgccaccgcc  
Depth:3 (HOXB5OS)  
Ei-value:Undefined, Pi-value:Undefined  
Er-value:0.000, Pr-value:0.000  
No matches to TargetScan

-252--(15)--268-

gccggga

gccggga  
Depth:3 (HOXB5OS)  
Ei-value:Undefined, Pi-value:Undefined  
Er-value:0.000, Pr-value:0.000  
No matches to TargetScan

-274--(12)--287-

ccacac

ccacac  
Depth:3 (HOXB5OS)  
Ei-value:Undefined, Pi-value:Undefined  
Er-value:0.000, Pr-value:0.000  
No matches to TargetScan

-292--(160)--453-

gg

ggtaaact  
Depth:3 (HOXB5OS)  
Ei-value:Undefined, Pi-value:Undefined  
Er-value:0.000, Pr-value:0.000  
No matches to TargetScan


taaact

taaact  
Depth:5 (HOXB_XENOPUS)  
Ei-value:Undefined, Pi-value:Undefined  
Er-value:0.000, Pr-value:0.000  
No matches to TargetScan

-460--(63)--524-

aatttagaa

aatttagaa  
Depth:3 (HOXB5OS)  
Ei-value:Undefined, Pi-value:Undefined  
Er-value:0.000, Pr-value:0.000  
No matches to TargetScan

-532
```

---

# Modules conserved to HOXB\_DOG\_ISOFORM1 (Depth: 2)

## Modules in Main Graph (All sequences considered):

```
>HOXB-AS3  
         1-

gtcata

gtcata  
Depth:5 (HOXB_XENOPUS)  
Ei-value:Undefined, Pi-value:Undefined  
Er-value:0.000, Pr-value:0.000  
No matches to eCLIP DataNo matches to TargetScan


gcgacttt

gcgacttt  
Depth:5 (HOXB_XENOPUS)  
Ei-value:Undefined, Pi-value:Undefined  
Er-value:0.000, Pr-value:0.000  
No matches to eCLIP DataNo matches to TargetScan


tggg

gtcatagcgacttttggg  
Depth:4 (HOXB_OPOSSUM)  
Ei-value:Undefined, Pi-value:Undefined  
Er-value:0.000, Pr-value:0.000  
No matches to eCLIP DataNo matches to TargetScan


a

gtcatagcgacttttgggatagtttgctat  
Depth:2 (HOXB_DOG_ISOFORM1)  
Ei-value:Undefined, Pi-value:Undefined  
Er-value:0.000, Pr-value:0.000  
No matches to eCLIP DataNo matches to TargetScan


tagtttgct

tagtttgct  
Depth:4 (HOXB_OPOSSUM)  
Ei-value:Undefined, Pi-value:Undefined  
Er-value:0.000, Pr-value:0.000  
No matches to eCLIP DataNo matches to TargetScan


at

gtcatagcgacttttgggatagtttgctat  
Depth:2 (HOXB_DOG_ISOFORM1)  
Ei-value:Undefined, Pi-value:Undefined  
Er-value:0.000, Pr-value:0.000  
No matches to eCLIP DataNo matches to TargetScan

-30--(1)--32-

ga

gacaaaggg  
Depth:2 (HOXB_DOG_ISOFORM1)  
Ei-value:Undefined, Pi-value:Undefined  
Er-value:0.000, Pr-value:0.000  
No matches to eCLIP DataNo matches to TargetScan


caaaggg

caaaggg  
Depth:4 (HOXB_OPOSSUM)  
Ei-value:Undefined, Pi-value:Undefined  
Er-value:0.000, Pr-value:0.000  
No matches to eCLIP DataNo matches to TargetScan

-40--(1)--42-

gacaaagtca

gacaaagtca  
Depth:3 (HOXB5OS)  
Ei-value:Undefined, Pi-value:Undefined  
Er-value:0.000, Pr-value:0.000  
No matches to eCLIP DataNo matches to TargetScan


agggg

gacaaagtcaagggg  
Depth:2 (HOXB_DOG_ISOFORM1)  
Ei-value:Undefined, Pi-value:Undefined  
Er-value:0.000, Pr-value:0.000  
No matches to eCLIP DataNo matches to TargetScan

-56--(8)--65-

aaggagg

aaggagg  
Depth:3 (HOXB5OS)  
Ei-value:Undefined, Pi-value:Undefined  
Er-value:0.000, Pr-value:0.000  
No matches to eCLIP DataNo matches to TargetScan


gcc

aaggagggcc  
Depth:2 (HOXB_DOG_ISOFORM1)  
Ei-value:Undefined, Pi-value:Undefined  
Er-value:0.000, Pr-value:0.000  
No matches to eCLIP DataNo matches to TargetScan

-74--(1)--76-

agtag

agtagagcctc  
Depth:2 (HOXB_DOG_ISOFORM1)  
Ei-value:Undefined, Pi-value:Undefined  
Er-value:0.000, Pr-value:0.000  
No matches to eCLIP DataMATCHES To TargetScan▶ miR-485-5p:GAGGCUG▶ miR-760:GGCUCUG


agcctc

agcctc  
Depth:3 (HOXB5OS)  
Ei-value:Undefined, Pi-value:Undefined  
Er-value:0.000, Pr-value:0.000  
No matches to eCLIP DataMATCHES To TargetScan▶ miR-485-5p:GAGGCUG

-86--(16)--103-

ct

ctcctcaccagctcccc  
Depth:2 (HOXB_DOG_ISOFORM1)  
Ei-value:Undefined, Pi-value:Undefined  
Er-value:0.000, Pr-value:0.000  
No matches to eCLIP DataMATCHES To TargetScan▶ miR-1224-5p:UGAGGAC▶ miR-138-5p:GCUGGUG


cctcacca

cctcacca  
Depth:3 (HOXB5OS)  
Ei-value:Undefined, Pi-value:Undefined  
Er-value:0.000, Pr-value:0.000  
No matches to eCLIP DataNo matches to TargetScan


g

gctcccc  
Depth:3 (HOXB5OS)  
Ei-value:Undefined, Pi-value:Undefined  
Er-value:0.000, Pr-value:0.000  
No matches to eCLIP DataNo matches to TargetScan


ctcccc

ctcccc  
Depth:4 (HOXB_OPOSSUM)  
Ei-value:Undefined, Pi-value:Undefined  
Er-value:0.010, Pr-value:0.000  
No matches to eCLIP DataNo matches to TargetScan

-119--(6)--126-

ccaagtcc

ccaagtcc  
Depth:2 (HOXB_DOG_ISOFORM1)  
Ei-value:Undefined, Pi-value:Undefined  
Er-value:0.000, Pr-value:0.000  
No matches to eCLIP DataNo matches to TargetScan

-133--(1)--135-

gtaagaagtt

gtaagaagtt  
Depth:4 (HOXB_OPOSSUM)  
Ei-value:Undefined, Pi-value:Undefined  
Er-value:0.000, Pr-value:0.000  
No matches to eCLIP DataNo matches to TargetScan


gggcc

gtaagaagttgggcc  
Depth:3 (HOXB5OS)  
Ei-value:Undefined, Pi-value:Undefined  
Er-value:0.000, Pr-value:0.000  
No matches to eCLIP DataNo matches to TargetScan


a

gtaagaagttgggccaagctggaagggattgaccggccg  
Depth:2 (HOXB_DOG_ISOFORM1)  
Ei-value:Undefined, Pi-value:Undefined  
Er-value:0.000, Pr-value:0.000  
No matches to eCLIP DataMATCHES To TargetScan▶ miR-188-5p:AUCCCUU▶ miR-204-5p/211-5p:UCCCUUU▶ miR-328-3p:UGGCCCU


agctg

agctggaagggattgaccg  
Depth:3 (HOXB5OS)  
Ei-value:Undefined, Pi-value:Undefined  
Er-value:0.000, Pr-value:0.000  
No matches to eCLIP DataMATCHES To TargetScan▶ miR-188-5p:AUCCCUU▶ miR-204-5p/211-5p:UCCCUUU


gaaggga

gaaggga  
Depth:4 (HOXB_OPOSSUM)  
Ei-value:Undefined, Pi-value:Undefined  
Er-value:0.000, Pr-value:0.000  
No matches to eCLIP DataMATCHES To TargetScan▶ miR-204-5p/211-5p:UCCCUUU


ttgaccg

agctggaagggattgaccg  
Depth:3 (HOXB5OS)  
Ei-value:Undefined, Pi-value:Undefined  
Er-value:0.000, Pr-value:0.000  
No matches to eCLIP DataMATCHES To TargetScan▶ miR-188-5p:AUCCCUU▶ miR-204-5p/211-5p:UCCCUUU


gccg

gtaagaagttgggccaagctggaagggattgaccggccg  
Depth:2 (HOXB_DOG_ISOFORM1)  
Ei-value:Undefined, Pi-value:Undefined  
Er-value:0.000, Pr-value:0.000  
No matches to eCLIP DataMATCHES To TargetScan▶ miR-188-5p:AUCCCUU▶ miR-204-5p/211-5p:UCCCUUU▶ miR-328-3p:UGGCCCU

-173--(9)--183-

cctcgcc

cctcgcc  
Depth:2 (HOXB_DOG_ISOFORM1)  
Ei-value:Undefined, Pi-value:Undefined  
Er-value:0.000, Pr-value:0.000  
No matches to eCLIP DataNo matches to TargetScan

-189--(0)--190-

ggcctc

ggcctc  
Depth:4 (HOXB_OPOSSUM)  
Ei-value:Undefined, Pi-value:Undefined  
Er-value:0.010, Pr-value:0.000  
No matches to eCLIP DataNo matches to TargetScan

-195--(1)--197-

gcggagat

gcggagattccaggccc  
Depth:3 (HOXB5OS)  
Ei-value:Undefined, Pi-value:Undefined  
Er-value:0.000, Pr-value:0.000  
No matches to eCLIP DataMATCHES To TargetScan▶ miR-216a-5p:AAUCUCA▶ miR-216b-5p:AAUCUCU


tccaggc

tccaggc  
Depth:4 (HOXB_OPOSSUM)  
Ei-value:Undefined, Pi-value:Undefined  
Er-value:0.000, Pr-value:0.000  
No matches to eCLIP DataNo matches to TargetScan


cc

gcggagattccaggccc  
Depth:3 (HOXB5OS)  
Ei-value:Undefined, Pi-value:Undefined  
Er-value:0.000, Pr-value:0.000  
No matches to eCLIP DataMATCHES To TargetScan▶ miR-216a-5p:AAUCUCA▶ miR-216b-5p:AAUCUCU


t

gcggagattccaggccct  
Depth:2 (HOXB_DOG_ISOFORM1)  
Ei-value:Undefined, Pi-value:Undefined  
Er-value:0.000, Pr-value:0.000  
No matches to eCLIP DataMATCHES To TargetScan▶ miR-216a-5p:AAUCUCA▶ miR-216b-5p:AAUCUCU

-214--(10)--225-

ggacgtccct

ggacgtccct  
Depth:2 (HOXB_DOG_ISOFORM1)  
Ei-value:Undefined, Pi-value:Undefined  
Er-value:0.000, Pr-value:0.000  
No matches to eCLIP DataNo matches to TargetScan

-234--(1)--236-

agc

agcgccaccgcc  
Depth:3 (HOXB5OS)  
Ei-value:Undefined, Pi-value:Undefined  
Er-value:0.000, Pr-value:0.000  
No matches to eCLIP DataNo matches to TargetScan


gccacc

gccacc  
Depth:5 (HOXB_XENOPUS)  
Ei-value:Undefined, Pi-value:Undefined  
Er-value:0.000, Pr-value:0.000  
No matches to eCLIP DataNo matches to TargetScan


gcc

agcgccaccgcc  
Depth:3 (HOXB5OS)  
Ei-value:Undefined, Pi-value:Undefined  
Er-value:0.000, Pr-value:0.000  
No matches to eCLIP DataNo matches to TargetScan

-247--(34)--282-

ccgcacc

ccgcacc  
Depth:2 (HOXB_DOG_ISOFORM1)  
Ei-value:Undefined, Pi-value:Undefined  
Er-value:0.000, Pr-value:0.000  
No matches to eCLIP DataNo matches to TargetScan

-288--(14)--303-

caggctgc

caggctgc  
Depth:2 (HOXB_DOG_ISOFORM1)  
Ei-value:Undefined, Pi-value:Undefined  
Er-value:0.000, Pr-value:0.000  
No matches to eCLIP DataNo matches to TargetScan

-310--(4)--315-

ggcggcgc

ggcggcgc  
Depth:2 (HOXB_DOG_ISOFORM1)  
Ei-value:Undefined, Pi-value:Undefined  
Er-value:0.000, Pr-value:0.000  
No matches to eCLIP DataNo matches to TargetScan

-322--(31)--354-

ccgggc

ccgggc  
Depth:2 (HOXB_DOG_ISOFORM1)  
Ei-value:Undefined, Pi-value:Undefined  
Er-value:0.090, Pr-value:0.010  
No matches to eCLIP DataNo matches to TargetScan

-359--(20)--380-

gagcg

gagcggccgggatgcggccacacc  
Depth:2 (HOXB_DOG_ISOFORM1)  
Ei-value:Undefined, Pi-value:Undefined  
Er-value:0.000, Pr-value:0.000  
No matches to eCLIP DataMATCHES To TargetScan▶ miR-324-5p:GCAUCCC


gccggga

gccggga  
Depth:3 (HOXB5OS)  
Ei-value:Undefined, Pi-value:Undefined  
Er-value:0.000, Pr-value:0.000  
No matches to eCLIP DataNo matches to TargetScan


tgcgg

gagcggccgggatgcggccacacc  
Depth:2 (HOXB_DOG_ISOFORM1)  
Ei-value:Undefined, Pi-value:Undefined  
Er-value:0.000, Pr-value:0.000  
No matches to eCLIP DataMATCHES To TargetScan▶ miR-324-5p:GCAUCCC


ccacac

ccacac  
Depth:3 (HOXB5OS)  
Ei-value:Undefined, Pi-value:Undefined  
Er-value:0.000, Pr-value:0.000  
No matches to eCLIP DataNo matches to TargetScan


c

gagcggccgggatgcggccacacc  
Depth:2 (HOXB_DOG_ISOFORM1)  
Ei-value:Undefined, Pi-value:Undefined  
Er-value:0.000, Pr-value:0.000  
No matches to eCLIP DataMATCHES To TargetScan▶ miR-324-5p:GCAUCCC

-403--(5)--409-

gg

ggtaaact  
Depth:3 (HOXB5OS)  
Ei-value:Undefined, Pi-value:Undefined  
Er-value:0.000, Pr-value:0.000  
No matches to eCLIP DataNo matches to TargetScan


taaact

taaact  
Depth:5 (HOXB_XENOPUS)  
Ei-value:Undefined, Pi-value:Undefined  
Er-value:0.000, Pr-value:0.000  
No matches to eCLIP DataNo matches to TargetScan

-416--(37)--454-

tccccac

tccccac  
Depth:2 (HOXB_DOG_ISOFORM1)  
Ei-value:Undefined, Pi-value:Undefined  
Er-value:0.000, Pr-value:0.000  
No matches to eCLIP DataMATCHES To TargetScan▶ miR-491-5p:GUGGGGA

-460--(14)--475-

ttttatttgg

ttttatttgg  
Depth:2 (HOXB_DOG_ISOFORM1)  
Ei-value:Undefined, Pi-value:Undefined  
Er-value:0.000, Pr-value:0.000  
No matches to eCLIP DataNo matches to TargetScan

-484--(12)--497-

aatttagaa

aatttagaa  
Depth:3 (HOXB5OS)  
Ei-value:Undefined, Pi-value:Undefined  
Er-value:0.000, Pr-value:0.000  
No matches to eCLIP DataNo matches to TargetScan

-505--(1)--507-

gagataaa

gagataaa  
Depth:2 (HOXB_DOG_ISOFORM1)  
Ei-value:Undefined, Pi-value:Undefined  
Er-value:0.000, Pr-value:0.000  
No matches to eCLIP DataNo matches to TargetScan

-514  
  
>HOXB_DOG_ISOFORM1  
        81-

gtcata

gtcata  
Depth:5 (HOXB_XENOPUS)  
Ei-value:Undefined, Pi-value:Undefined  
Er-value:0.000, Pr-value:0.000  
No matches to TargetScan


gcgacttt

gcgacttt  
Depth:5 (HOXB_XENOPUS)  
Ei-value:Undefined, Pi-value:Undefined  
Er-value:0.000, Pr-value:0.000  
No matches to TargetScan


tggg

gtcatagcgacttttggg  
Depth:4 (HOXB_OPOSSUM)  
Ei-value:Undefined, Pi-value:Undefined  
Er-value:0.000, Pr-value:0.000  
No matches to TargetScan


a

gtcatagcgacttttgggatagtttgctat  
Depth:2 (HOXB_DOG_ISOFORM1)  
Ei-value:Undefined, Pi-value:Undefined  
Er-value:0.000, Pr-value:0.000  
No matches to TargetScan


tagtttgct

tagtttgct  
Depth:4 (HOXB_OPOSSUM)  
Ei-value:Undefined, Pi-value:Undefined  
Er-value:0.000, Pr-value:0.000  
No matches to TargetScan


at

gtcatagcgacttttgggatagtttgctat  
Depth:2 (HOXB_DOG_ISOFORM1)  
Ei-value:Undefined, Pi-value:Undefined  
Er-value:0.000, Pr-value:0.000  
No matches to TargetScan

-110--(1)--112-

ga

gacaaaggg  
Depth:2 (HOXB_DOG_ISOFORM1)  
Ei-value:Undefined, Pi-value:Undefined  
Er-value:0.000, Pr-value:0.000  
No matches to TargetScan


caaaggg

caaaggg  
Depth:4 (HOXB_OPOSSUM)  
Ei-value:Undefined, Pi-value:Undefined  
Er-value:0.000, Pr-value:0.000  
No matches to TargetScan

-120--(1)--122-

gacaaagtca

gacaaagtca  
Depth:3 (HOXB5OS)  
Ei-value:Undefined, Pi-value:Undefined  
Er-value:0.000, Pr-value:0.000  
No matches to TargetScan


agggg

gacaaagtcaagggg  
Depth:2 (HOXB_DOG_ISOFORM1)  
Ei-value:Undefined, Pi-value:Undefined  
Er-value:0.000, Pr-value:0.000  
No matches to TargetScan

-136--(8)--145-

aaggagg

aaggagg  
Depth:3 (HOXB5OS)  
Ei-value:Undefined, Pi-value:Undefined  
Er-value:0.000, Pr-value:0.000  
No matches to TargetScan


gcc

aaggagggcc  
Depth:2 (HOXB_DOG_ISOFORM1)  
Ei-value:Undefined, Pi-value:Undefined  
Er-value:0.000, Pr-value:0.000  
No matches to TargetScan

-154--(1)--156-

agtag

agtagagcctc  
Depth:2 (HOXB_DOG_ISOFORM1)  
Ei-value:Undefined, Pi-value:Undefined  
Er-value:0.000, Pr-value:0.000  
MATCHES To TargetScan▶ miR-485-5p:GAGGCUG▶ miR-760:GGCUCUG


agcctc

agcctc  
Depth:3 (HOXB5OS)  
Ei-value:Undefined, Pi-value:Undefined  
Er-value:0.000, Pr-value:0.000  
MATCHES To TargetScan▶ miR-485-5p:GAGGCUG

-166--(16)--183-

ct

ctcctcaccagctcccc  
Depth:2 (HOXB_DOG_ISOFORM1)  
Ei-value:Undefined, Pi-value:Undefined  
Er-value:0.000, Pr-value:0.000  
MATCHES To TargetScan▶ miR-1224-5p:UGAGGAC▶ miR-138-5p:GCUGGUG


cctcacca

cctcacca  
Depth:3 (HOXB5OS)  
Ei-value:Undefined, Pi-value:Undefined  
Er-value:0.000, Pr-value:0.000  
No matches to TargetScan


g

gctcccc  
Depth:3 (HOXB5OS)  
Ei-value:Undefined, Pi-value:Undefined  
Er-value:0.000, Pr-value:0.000  
No matches to TargetScan


ctcccc

ctcccc  
Depth:4 (HOXB_OPOSSUM)  
Ei-value:Undefined, Pi-value:Undefined  
Er-value:0.010, Pr-value:0.000  
No matches to TargetScan

-199--(6)--206-

ccaagtcc

ccaagtcc  
Depth:2 (HOXB_DOG_ISOFORM1)  
Ei-value:Undefined, Pi-value:Undefined  
Er-value:0.000, Pr-value:0.000  
No matches to TargetScan

-213--(1)--215-

gtaagaagtt

gtaagaagtt  
Depth:4 (HOXB_OPOSSUM)  
Ei-value:Undefined, Pi-value:Undefined  
Er-value:0.000, Pr-value:0.000  
No matches to TargetScan


gggcc

gtaagaagttgggcc  
Depth:3 (HOXB5OS)  
Ei-value:Undefined, Pi-value:Undefined  
Er-value:0.000, Pr-value:0.000  
No matches to TargetScan


a

gtaagaagttgggccaagctggaagggattgaccggccg  
Depth:2 (HOXB_DOG_ISOFORM1)  
Ei-value:Undefined, Pi-value:Undefined  
Er-value:0.000, Pr-value:0.000  
MATCHES To TargetScan▶ miR-188-5p:AUCCCUU▶ miR-204-5p/211-5p:UCCCUUU▶ miR-328-3p:UGGCCCU


agctg

agctggaagggattgaccg  
Depth:3 (HOXB5OS)  
Ei-value:Undefined, Pi-value:Undefined  
Er-value:0.000, Pr-value:0.000  
MATCHES To TargetScan▶ miR-188-5p:AUCCCUU▶ miR-204-5p/211-5p:UCCCUUU


gaaggga

gaaggga  
Depth:4 (HOXB_OPOSSUM)  
Ei-value:Undefined, Pi-value:Undefined  
Er-value:0.000, Pr-value:0.000  
MATCHES To TargetScan▶ miR-204-5p/211-5p:UCCCUUU


ttgaccg

agctggaagggattgaccg  
Depth:3 (HOXB5OS)  
Ei-value:Undefined, Pi-value:Undefined  
Er-value:0.000, Pr-value:0.000  
MATCHES To TargetScan▶ miR-188-5p:AUCCCUU▶ miR-204-5p/211-5p:UCCCUUU


gccg

gtaagaagttgggccaagctggaagggattgaccggccg  
Depth:2 (HOXB_DOG_ISOFORM1)  
Ei-value:Undefined, Pi-value:Undefined  
Er-value:0.000, Pr-value:0.000  
MATCHES To TargetScan▶ miR-188-5p:AUCCCUU▶ miR-204-5p/211-5p:UCCCUUU▶ miR-328-3p:UGGCCCU

-253--(6)--260-

cctcgcc

cctcgcc  
Depth:2 (HOXB_DOG_ISOFORM1)  
Ei-value:Undefined, Pi-value:Undefined  
Er-value:0.000, Pr-value:0.000  
No matches to TargetScan

-266--(6)--273-

ggcctc

ggcctc  
Depth:4 (HOXB_OPOSSUM)  
Ei-value:Undefined, Pi-value:Undefined  
Er-value:0.010, Pr-value:0.000  
No matches to TargetScan

-278--(1)--280-

gcggagat

gcggagattccaggccc  
Depth:3 (HOXB5OS)  
Ei-value:Undefined, Pi-value:Undefined  
Er-value:0.000, Pr-value:0.000  
MATCHES To TargetScan▶ miR-216a-5p:AAUCUCA▶ miR-216b-5p:AAUCUCU


tccaggc

tccaggc  
Depth:4 (HOXB_OPOSSUM)  
Ei-value:Undefined, Pi-value:Undefined  
Er-value:0.000, Pr-value:0.000  
No matches to TargetScan


cc

gcggagattccaggccc  
Depth:3 (HOXB5OS)  
Ei-value:Undefined, Pi-value:Undefined  
Er-value:0.000, Pr-value:0.000  
MATCHES To TargetScan▶ miR-216a-5p:AAUCUCA▶ miR-216b-5p:AAUCUCU


t

gcggagattccaggccct  
Depth:2 (HOXB_DOG_ISOFORM1)  
Ei-value:Undefined, Pi-value:Undefined  
Er-value:0.000, Pr-value:0.000  
MATCHES To TargetScan▶ miR-216a-5p:AAUCUCA▶ miR-216b-5p:AAUCUCU

-297--(10)--308-

ggacgtccct

ggacgtccct  
Depth:2 (HOXB_DOG_ISOFORM1)  
Ei-value:Undefined, Pi-value:Undefined  
Er-value:0.000, Pr-value:0.000  
No matches to TargetScan

-317--(1)--319-

agc

agcgccaccgcc  
Depth:3 (HOXB5OS)  
Ei-value:Undefined, Pi-value:Undefined  
Er-value:0.000, Pr-value:0.000  
No matches to TargetScan


gccacc

gccacc  
Depth:5 (HOXB_XENOPUS)  
Ei-value:Undefined, Pi-value:Undefined  
Er-value:0.000, Pr-value:0.000  
No matches to TargetScan


gcc

agcgccaccgcc  
Depth:3 (HOXB5OS)  
Ei-value:Undefined, Pi-value:Undefined  
Er-value:0.000, Pr-value:0.000  
No matches to TargetScan

-330--(33)--364-

ccgcacc

ccgcacc  
Depth:2 (HOXB_DOG_ISOFORM1)  
Ei-value:Undefined, Pi-value:Undefined  
Er-value:0.000, Pr-value:0.000  
No matches to TargetScan

-370--(635)--1006-

caggctgc

caggctgc  
Depth:2 (HOXB_DOG_ISOFORM1)  
Ei-value:Undefined, Pi-value:Undefined  
Er-value:0.000, Pr-value:0.000  
No matches to TargetScan

-1013--(1573)--2587-

ggcggcgc

ggcggcgc  
Depth:2 (HOXB_DOG_ISOFORM1)  
Ei-value:Undefined, Pi-value:Undefined  
Er-value:0.000, Pr-value:0.000  
No matches to TargetScan

-2594--(2)--2597-

ccgggc

ccgggc  
Depth:2 (HOXB_DOG_ISOFORM1)  
Ei-value:Undefined, Pi-value:Undefined  
Er-value:0.090, Pr-value:0.010  
No matches to TargetScan

-2602--(3)--2606-

gagcg

gagcggccgggatgcggccacacc  
Depth:2 (HOXB_DOG_ISOFORM1)  
Ei-value:Undefined, Pi-value:Undefined  
Er-value:0.000, Pr-value:0.000  
MATCHES To TargetScan▶ miR-324-5p:GCAUCCC


gccggga

gccggga  
Depth:3 (HOXB5OS)  
Ei-value:Undefined, Pi-value:Undefined  
Er-value:0.000, Pr-value:0.000  
No matches to TargetScan


tgcgg

gagcggccgggatgcggccacacc  
Depth:2 (HOXB_DOG_ISOFORM1)  
Ei-value:Undefined, Pi-value:Undefined  
Er-value:0.000, Pr-value:0.000  
MATCHES To TargetScan▶ miR-324-5p:GCAUCCC


ccacac

ccacac  
Depth:3 (HOXB5OS)  
Ei-value:Undefined, Pi-value:Undefined  
Er-value:0.000, Pr-value:0.000  
No matches to TargetScan


c

gagcggccgggatgcggccacacc  
Depth:2 (HOXB_DOG_ISOFORM1)  
Ei-value:Undefined, Pi-value:Undefined  
Er-value:0.000, Pr-value:0.000  
MATCHES To TargetScan▶ miR-324-5p:GCAUCCC

-2629--(5)--2635-

gg

ggtaaact  
Depth:3 (HOXB5OS)  
Ei-value:Undefined, Pi-value:Undefined  
Er-value:0.000, Pr-value:0.000  
No matches to TargetScan


taaact

taaact  
Depth:5 (HOXB_XENOPUS)  
Ei-value:Undefined, Pi-value:Undefined  
Er-value:0.000, Pr-value:0.000  
No matches to TargetScan

-2642--(46)--2689-

tccccac

tccccac  
Depth:2 (HOXB_DOG_ISOFORM1)  
Ei-value:Undefined, Pi-value:Undefined  
Er-value:0.000, Pr-value:0.000  
MATCHES To TargetScan▶ miR-491-5p:GUGGGGA

-2695--(7)--2703-

ttttatttgg

ttttatttgg  
Depth:2 (HOXB_DOG_ISOFORM1)  
Ei-value:Undefined, Pi-value:Undefined  
Er-value:0.000, Pr-value:0.000  
No matches to TargetScan

-2712--(11)--2724-

aatttagaa

aatttagaa  
Depth:3 (HOXB5OS)  
Ei-value:Undefined, Pi-value:Undefined  
Er-value:0.000, Pr-value:0.000  
No matches to TargetScan

-2732--(1)--2734-

gagataaa

gagataaa  
Depth:2 (HOXB_DOG_ISOFORM1)  
Ei-value:Undefined, Pi-value:Undefined  
Er-value:0.000, Pr-value:0.000  
No matches to TargetScan

-2741
```

---
