# Supplementary material for: Highly conserved and cis-acting lncRNAs produced from paralogous regions in the center of HOXA and HOXB clusters in the endoderm lineage
Source: PLoS Genet. 2021 Jul 19;17(7):e1009681. doi: 10.1371/journal.pgen.1009681 (PMC8330917; doi:10.1371/journal.pgen.1009681)
Supplement: S1 Dataset — (ZIP) [file pgen.1009681.s015.zip › HOXB-AS3_var1/Html_Files/kmers_in_seqs_level.html]

 MOTIFS\_IN\_SEQS\_OVERLAP

# MOTIFS IN SEQUENCES

## Motifs conserved to (and beyond) HOXB\_XENOPUS (depth:5)

  

NAVIGATE ▼

▶HOXB-AS3 (depth:1)▶HOXB\_DOG\_ISOFORM1 (depth:2)▶HOXB5OS (depth:3)▶HOXB\_OPOSSUM (depth:4)▶HOXB\_XENOPUS (depth:5)▶HOXB\_COELACANTH\_HOXB (depth:6)▶HOXB\_GAR (depth:7)▶HOXB\_SHARK (depth:8)

  
  
  
  
Coloured by Conservation >>  

## >HOXB-AS3 (573 bases)

```
gtcata

gtcata  
Depth:5 (HOXB_XENOPUS)  
Ei-value:Undefined, Pi-value:Undefined  
Er-value:0.000, Pr-value:0.000  
No matches to eCLIP DataNo matches to TargetScan


gcgacttt

gcgacttt  
Depth:5 (HOXB_XENOPUS)  
Ei-value:Undefined, Pi-value:Undefined  
Er-value:0.000, Pr-value:0.000  
No matches to eCLIP DataNo matches to TargetScan

TGGGATAGTTTGCTATCGACAAAGGGAGACAAAGTCAAGGGGTGAAGGGAAAGGAGGGCCAAGTAGAGCCTCCACGACCCTCGGCTTCCTCCTCACCAGCTCCCCC 120  
 TCCCTCCAAGTCCAGTAAGAAGTTGGGCCAAGCTGGAAGGGATTGACCGG||CCGTTTCCTCTCCCTCGCCGGCCTCGGCGGAGATTCCAGGCCCTATAGAAACCAGGACGTCCCTTAGC 238  


gccacc

gccacc  
Depth:5 (HOXB_XENOPUS)  
Ei-value:Undefined, Pi-value:Undefined  
Er-value:0.000, Pr-value:0.000  
No matches to eCLIP DataNo matches to TargetScan

GCCTCACATGCCAGTGCTGCCGGGAACCCAGCGATATCCGCACCAG||CGGAGAAGGTTCCAGGCTGCCGGCGGCGGCGCAGAGAGCGGGAAGAGAGGCTCGGAGGAAGCCCCG 356  
 GGCGTGGCGTGGTCAGGCTCCGAGAGCGGCCGGGATGCGGCCACACCGGCCTGG

taaact

taaact  
Depth:5 (HOXB_XENOPUS)  
Ei-value:Undefined, Pi-value:Undefined  
Er-value:0.000, Pr-value:0.000  
No matches to eCLIP DataNo matches to TargetScan

CGCACCTCTTAGGATCTTGCTCCCGGACTCATTCCCTTCCCCACCCCCTATTTTAAAGTT 476  
 TTATTTGGGTCGTCTGTATCAATTTAGAACGAGATAAATTAAGACAAAGAAAGTAAAATAAATCGAAATAAAATATAGGAATAGCTCTTGGCGAAAA                        573
```

---

## >HOXB\_DOG\_ISOFORM1 (2741 bases)

```
 TGAGGGGTATCTGTCTGACTTCTCGGCGATTTTTACGATCTAACTTCGAGATAAAACCCCTATCCATTTGACATCTAAAT

gtcata

gtcata  
Depth:5 (HOXB_XENOPUS)  
Ei-value:Undefined, Pi-value:Undefined  
Er-value:0.000, Pr-value:0.000  
No matches to TargetScan


gcgacttt

gcgacttt  
Depth:5 (HOXB_XENOPUS)  
Ei-value:Undefined, Pi-value:Undefined  
Er-value:0.000, Pr-value:0.000  
No matches to TargetScan

TGGGATAGTTTGCTATGGACAAAGGG 120  
 GGACAAAGTCAAGGGGCGAGGGGGAAGGAGGGCCCAGTAGAGCCTCTACGATTCTTGGCTGTCTCCTCACCAGCTCCCCTCCCCCCCAAGTCCTGTAAGAAGTTGGGCCAAGCTGGAAGG 240  
 GATTGACCGG||CCGCCGCCTCCTCGCCCTCGAGGGCCTCTGCGGAGATTCCAGGCCCTCCAGAGACCCGGACGTCCCTCAGC

gccacc

gccacc  
Depth:5 (HOXB_XENOPUS)  
Ei-value:Undefined, Pi-value:Undefined  
Er-value:0.000, Pr-value:0.000  
No matches to TargetScan

GCCCCTGTGCCAATGCCGCTGGGAAACCGCC 358  
 GAGACCCGCACCGGGCAAGAGAACTGCAGGGGGCCAACTGGGGGAGGCCGGAACGAGGGAGGGAAGGGGGGAGGGGGCGCACACCCACAGAGGCCAGAGCGACCGGCTTCTGGGCGCCCT 478  
 GAAGCCCAGACACCCCTATGCTGTCTTTCGCAGCCCCCTCTCTGGAGCCTCCTCTCTCAGCTTAAAAGGGCGACTTAGAGCTCGCCTCCGTGGCCCCTTTCGGTTCCTCCTCCCATCTTC 598  
 CCTGCCTTGGCTCCCCCTCCAGGAAGCAGCCACCCCTCTCTCTTTCTCAGCCACCGAATTTCCTGGGAAGCCTCTCCCTGCAGCGCCCAGAGATGGGGTTCAGCCCCGTCTTGGGGTGGG 718  
 GGGAGAGGGAGACAAACCTAAGCCCCACCCCCTCCAAAATCGATGCAAAAGAATAAGAATTGTAGAATCTCCCTTACCTTTGTAGACCTTGGGATTTCTTCCCTCCGATTGAAAGGGGTG 838  
 AAGAGGAAGGGGGGGGGGAAGCGTTCTGTTTCTTCTTCATCTTCCAGATAATATTGTCCCAACCTGAGAGCCGTCGCTTCCCCTTCTCTGACTTGGGAGAGAGGGTGTTTCGCTTTTCTG 958  
 ATATTTGCATAGAAAATGGAGTAAGTTCTGGCTTTGAAAAATGGTATCAGGCTGCAGTCCTGGGCAGATTGTCCATTTACCCTCCATGATGACTAAAATCCCAGATCAGCAAGCAAATCA 1078  
 ACTTAGAAATTAAAATTATTTTTGGTTCTCTCTTTTGTGTAATTCCCACCCCTCCTTCTTTCTCTCTTTCTCTCTCTCTCTCTCTCTCTCTCTCTCTCTCCCTCCTTTCCCTCTCTCTTC 1198  
 CTCTCTCTCTTTTGCTTTATCGAGCTGATATTGAAGTATAAAAATATCAAGAGGCTGGAGCCCTGAAGGACGACAGTGCTCCGACCTAGGTGTGGTGTCCAAAAGAATGCTGCATTATAA 1318  
 CCACCAGGGAAATGATAAAAAGTTCATGTTCACGATCGCCCGGCCACATGACCGGCGCCGGCCAATCGCTGGATTCAACCACTCATAAACTTCTATCACAAAGTTGTAAATTTTCATAAA 1438  
 ACAACAAGGAATTTATTGCATTTCTTCATGGCTGCTCCACCAGCAACCCTTTTCTCGGTCGCCATCTTCTTTTCTTCTCCTTCTCGCTATTTGGGGAAACCCCAATCTGAGAAGGGATGA 1558  
 GATTTGGGGTGCAGGGAATCAGACTGAGGAGCCACAGGGGTCCATCTGGAGAGGGGATTCTCTCCAGAACCTCCCTATTCTCACAGGAGCTCTGCCTCCCTCTTCTAGTAAGAATTCACC 1678  
 CCAAATCCTCCACACAGGGGCCATTCTAAGGCAGTTTTGGGGTGAGTCGGGAGTAACAGCGGGTATCCAAAATTGCCACTTGCATCAAGGCAAAATGAAGGCTTCAGGGTCCCCTCTCCT 1798  
 CCTCTGAATGCCCCCCTCATTCCCAGCCCTCTAGATGGTGGGACCCCCACCTGTGAGGCGTGTAGCATTGCGGGTGTGGGGGTCTTAGATCCCCTAGGGCCTAGTTTTCACCTAGGAAAG 1918  
 AAAGGCGGCCCTGACAGAGGCCAAGGTGGACAGCCTAGCAACCAGAGTCTCGCCTCTGCACCCCAGAAACTGGAAGAGGGTGAAGCCAGATCCCTGCCTCTTCCACCCAAATGCCCACAT 2038  
 GCTCAGGTTCCCAATGACAGCTCCATGTGCTCTCCAGGGGGTTCTGCGGTCATTAATGGGGGAAAGTGCCGGTTCAGGGGGACTTTTACCTACAAATTCAAATTAAAGAGATTCTCCTAA 2158  
 AACGCGCTTCCATTCAAACCTGCTTCTTTCTTTCTGTCCCAAAACCTGTTCTTTGCATTTAAATAATCCAAGGTCTGTAATCGAATAAATTCTAAAAATCAGCTCCACAGGTCTAGATGC 2278  
 GTAGGCACCGTCCTCCAGGTTTATGTTCCTCAATCAAGGGGTGTTTTGGAGGAAAAAACCCTACAAAACAAACAAAAAAATCTCCACCGCCACCAAACAGAATTCACCCCGTGTTTCCCA 2398  
 GGGCCCCTGAAATACTGCTGTATTTTGAATTGAGCTACCAGCGCCCAAGTGCAGGAAACGAAATAACATCCCCCAAAAAACGTTTTCTCTTTTTTCTCTCTTTCTCTGTATTCCCGAGGA 2518  
 GATTCCAGGTTGCCGGCAGCGCGGACCGCGGGCAGCAAGGCGCGGAGGGAGCCCGCGGAGCTTGGGCGGGCGGCGCGGCCGGGCTCCGAGCGGCCGGGATGCGGCCACACCAGCCGGG

ta

taaact  
Depth:5 (HOXB_XENOPUS)  
Ei-value:Undefined, Pi-value:Undefined  
Er-value:0.000, Pr-value:0.000  
No matches to TargetScan

 2638  


aact

taaact  
Depth:5 (HOXB_XENOPUS)  
Ei-value:Undefined, Pi-value:Undefined  
Er-value:0.000, Pr-value:0.000  
No matches to TargetScan

TGCACCGCTCAGACTCTCGTTCTCAGGGCTCATCCACTCCCCCACCTCCCCACGTAAAAATTTTATTTGGATCGCTGTGTTAATTTAGAATGAGATAAA                  2741
```

---

## >HOXB5OS (596 bases)

```
 AT

gtcata

gtcata  
Depth:5 (HOXB_XENOPUS)  
Ei-value:Undefined, Pi-value:Undefined  
Er-value:0.000, Pr-value:0.000  
No matches to TargetScan


gcgacttt

gcgacttt  
Depth:5 (HOXB_XENOPUS)  
Ei-value:Undefined, Pi-value:Undefined  
Er-value:0.000, Pr-value:0.000  
No matches to TargetScan

TGGGGTAGTTTGCTTTCGGCAAAGGGGGACAAAGTCATGGGGTGAGAGGGAAGGAGGACCCAAAACAGCCTCCAAGACCCTCACCATCTCTTCACCGGCTCCCC 120  
 CTCCCCCCCCACAGGTCCTGTAAGAAGTTGGGCCCAGCTGGAAGGGATTGACCG||ACGCCTCTTCGCCCTAGGCTGGCCTCTGCGGAGATTCCAGGCCCCACAGAGACCAGGACTTCCC 238  
 TCAGC

gccacc

gccacc  
Depth:5 (HOXB_XENOPUS)  
Ei-value:Undefined, Pi-value:Undefined  
Er-value:0.000, Pr-value:0.000  
No matches to TargetScan

GCCCCTCGTGCCAATGCAGCCGGGAAATCGCCATTACCCACACTGGGCAAGAGAAACCGCACGGGGGCAATCGGAGAGGCCAGGAAAAGGGAGAGATGGAGGGAG||AA 356  
 GGTTCCAGGTTGGCCGTACTCAGGAGCAGGCCGAACGGCCCAGAGGAAGCCCTGGCGTCCGGTGCTGCCAGGCTCACTGCGGACGCACCCCAGCCCGG

taaact

taaact  
Depth:5 (HOXB_XENOPUS)  
Ei-value:Undefined, Pi-value:Undefined  
Er-value:0.000, Pr-value:0.000  
No matches to TargetScan

CACTGTCGCTCGCCGG 476  
 GACTGGTTTCCCTTCAATTTAAAAATTTCATGTGGATCGCCCATGCCAATTTAGAATGAAATAGATTTAAATACATAAAGTAAAAATAAATTGAAATAAAAGCAAGGCATCTGATGAAAA 596  
                                                                                                                          596
```

---

## >HOXB\_OPOSSUM (1381 bases)

```
 ATCTAACCCCCAAGCCCCCATTGCCCTAAACACGCAGCGAAGAGAGGGAGAGGAACCGGACTTACTCTGGAGAACGCTGGACCTCGGGCCTCCCTCCGTTGTCTCCGCCGCCGCCGCCGC 120  
 CACCGTCGCTGCCACCTCTGCTTTTGCTGCTGCTGAGGCCGGGCCCTCAGCTCCTCTCAGCTGGGAGAGAGCTCACCTACCTCTGCCCCCTTCCCCGCCACTGCGAGCAGTTAAAGTGTC 240  
 ACTTACATTCTCGAGAATGTGAAATATACCGCGCGGTGTCAACTCCCCAAAACCATAAAACTAACTTTATGGACCTCACGTGACTTTCTCGAGCCAGTGAGGGGTATCTGTCTGACTTCT 360  
 CGGCGATTTTTACGATCTAACTTCGAGATAAAACCCCTATCCATTTGACATCTAAAT

gtcata

gtcata  
Depth:5 (HOXB_XENOPUS)  
Ei-value:Undefined, Pi-value:Undefined  
Er-value:0.000, Pr-value:0.000  
No matches to TargetScan


gcgacttt

gcgacttt  
Depth:5 (HOXB_XENOPUS)  
Ei-value:Undefined, Pi-value:Undefined  
Er-value:0.000, Pr-value:0.000  
No matches to TargetScan

TGGGATAGTTTGCTATCGATAAAGGAAAAAAAATAAAGGGGGGGAGGGG 480  
 CAAAGGGGGGCGAACAGACACTCCTGGACTTTCGACCGGTCGCCTCCCTAGCTCTCCCCCCCACCCCCCAAGTCCTGTAAGAAGTTTGGCTAAGAAGGGAGAGATTGAGCTGG||CCAGT 598  
 TCCCCAACCCTTACACGGAGTCCCTGCTCCTGCTGCCCTTCTTAGAACCAGGGCCTCCAGGGATCTTTCCGATCCAGCTTGTTTCCCGTGCCAACTCCACCTGGACGCCTTGAAAACCCA 718  
 GCGTCAGACAAGAAAACTCCACTGGCACAATTGAGGCGGGGGTGAAGAAGGACTTGAACCCTCAGAGGCTAGAGAG||ATGTAAGGGAATACGACCATGCTGGGGACCATAAGGAACAGA 836  
 ACTAAAGAAGTGGGGAGCCACTAGGAGCACGGAGGCCCTCCCCGACTGACGCATTGCGGTTGCCTCCTGGTCTAAGGAATCCAGGCACCAACTGACCTCTTCAAACCCCAAGTTTACCAA 956  
 T

gccacc

gccacc  
Depth:5 (HOXB_XENOPUS)  
Ei-value:Undefined, Pi-value:Undefined  
Er-value:0.000, Pr-value:0.000  
No matches to TargetScan

TTAGGAATCACTTCTTCTCTTTTCTCTCCTTTTTCTTGCCCTCTGCTTTTCTTCTTTCTTTCTTAATTCCATTTACTCCCCCCCTCCCACCCCTTCTTCTCCACCTCTTCTAT 1076  
 CCGTGGAACCATAATGAAATCAAATAAAAGAAATTAGGGCCGAAAGAGAGCTTGCTCAGGAAGGCGGTTAATAAAGGGAAGCTGGTTTGAGGCACTTGATTTCCCCGTCTAATTGCCTTC 1196  
 ATTGGCTTTCCCTACTTTGATGTGGAACAGGATTTGGCGCTAATAAAATAAGGACTTTCGGTCTGGGTCTGCAA

taaact

taaact  
Depth:5 (HOXB_XENOPUS)  
Ei-value:Undefined, Pi-value:Undefined  
Er-value:0.000, Pr-value:0.000  
No matches to TargetScan

CATCTCCACACAAAATCAACCAAGTCTTCTTCCCTGCGTC 1316  
 TTCCTTTCCTAATGACCAGAGTGAAAAACATATATGTTATTTTCTTGCTGCTGAATTTTTTCAGT                                                        1381
```

---

## >HOXB\_XENOPUS (647 bases)

```
 TCTGGGCCATTTGTACCATGGAACTTCTACATAAAACCCAATCCATTTGACATGGAAAT

gtcata

gtcata  
Depth:5 (HOXB_XENOPUS)  
Ei-value:Undefined, Pi-value:Undefined  
Er-value:0.000, Pr-value:0.000  
No matches to TargetScan

CCTACTTGGGCTTTACTTTGCCCAAATCACCAAAGGGGACGGGGATGCCTTGGCT 120  
 TCACTCTGCTCTGGG||GGGAGCTGTATTTTAGCCTGATCGAATTTTCGTGGCTGCACAGAACAAACAGAAGAAAAGACTTTGCGGAACGGGGGGGAAAAAAAATGCAACAAAGTCATCT 238  
 GCTTTCCAGATTATTCGCCATAAACCACCCTCCCAATTCCCATGCCTGTAACTAAATAATCCGCTAGAAACGCAGCAAAACTGGTGTCATCTCCACAAAGTGTAAAAACCTGGAATTCAG 358  
 TGAGTAATT

gcgacttt

gcgacttt  
Depth:5 (HOXB_XENOPUS)  
Ei-value:Undefined, Pi-value:Undefined  
Er-value:0.000, Pr-value:0.000  
No matches to TargetScan


gccacc

gccacc  
Depth:5 (HOXB_XENOPUS)  
Ei-value:Undefined, Pi-value:Undefined  
Er-value:0.000, Pr-value:0.000  
No matches to TargetScan

TCTGTAACAGCATCAATTTGTCTGCCCTGCTCTGTGCAACACTTTCAGCTTCCCCCTTGGGTTACATAAGGGTGCAAAGAATCCCAATGATTGAGAA 478  
 AAGTTTATTTTCATTGCCAATGTGTTAACCTATTACATCATGGTTTTTATCA

taaact

taaact  
Depth:5 (HOXB_XENOPUS)  
Ei-value:Undefined, Pi-value:Undefined  
Er-value:0.000, Pr-value:0.000  
No matches to TargetScan

GCTCCGACCCTGTGTAAATAAATGCTTCTTAACGAGTGTGGGTGCTTATGATTTCCTCATTA 598  
 ACAACCTTGTTATACTCCAATTTACAGTATTTATATTTGAACGTGTCAG                                                                        647
```

---

## >HOXB\_COELACANTH\_HOXB (974 bases)

```
 NO CONSERVED NODES FOUND  
AAAACACTAATACTAAGCTTTTCAACTGCATGTCATGGACGATTTACAATTTTTGGAGAATAAGAAAGTTTAGAACTCATTGGATTCATCTAAAAGTGCATGATATTTAATCGCCTAACG 120  
 GGATGACACATAGGTGAGTTTTGATTAAATAATTAGCTTGTTCAAATTTTGCATTGGAGTGTTGTGTGATTTAGCAACGCTCTGGTTGTCTCGTGCGAACTTAAATTTGTTCAGGCGGTA 240  
 GGAAAATAGATTCAATGGCCGGCAGCAAATTTTATCTAAAAGAGGATGCAATGACTGTGAACTTGGTCTGAAGCGGGCTCTGCTGGTGGTTTGTGGTGTTACAG||ATCCTAGAGAATCA 358  
 ATTGTTCTACTGTCGGTATACCATTAAAGAGAGGAAGCCAATGAAAAATGAATTTGAAGTCTTCGCCTTTCTACACG||GGTTTTTCCTTTGTATATGGCTTGAAATCAGACCTATCCAA 476  
 AGTTTGCATTATTCACGCCAAGAATGTGGAAAAAGAGAAAAAGGAAACCGAACTCTTGAAACGGTTTGAAAAGAAAACGCCCTTAACAAGAAGTAATATCCAATTTAGGGAGGGGGCAAC 596  
 TAAGCAGGAGAATAACTAACTGGGGACATTGCAAAAACGTTTATAAATACAATTGTATCTATTTTCAAGTTAAAAGAAACAAGAAAGCGTTTGAAGACAACCCACTCCCTTCTTATTGGA 716  
 GAATGTTAAAAGAAGTTTGAAACCCTTATATTTAACTGCGTGAGTATTTCTTCTATGCATTATATTTTCTTGTACTTATACTTTAATGTATTGGATTAATTGTATTTTTTTTATCTGTAA 836  
 ATACCTTTTATAGCAATAGTTAGATGATCAGCATTGCCTTTGTAACTATTGTTGACTTCAGAACTGGATTATCTAACCGCAGTTTTAGTTTCCTTTATTGCACTGTATTTAAAACATGGA 956  
 AAAATAAATTGAAATGAG                                                                                                       974
```

---

## >HOXB\_GAR (2133 bases)

```
 NO CONSERVED NODES FOUND  
TTTGGTATACTTTGGCAAAGGGTACAGCAAAATTTCAAAACGCGGGGAGGCAGAGAAAGGCAGATGGTTACATTTGGCTTGAGAGCGCCTCCACTCCACCCCGAGCTCTCAAAAGAAATG 120  
 TGGGAAGTATCTCAACAGTGGGGGATTAATTG||GGTTTTCCTTTGTGAAACCGGTCGGACTCCGAGCAGCACAAACACATTTTCGAACTCGCCCCCTGAAAGAAACCAAGCGACAAACT 238  
 GTTTGGGGAGGGAGGGGGGAATTCAGAAGCCAGTGTCGCCAGGAGGAAACTGGGACAAGCAGCGAGAGAGCCCGGAATCTCAGCGGAAACACCGCCGAGGAATCGCACCTACGAGAACAG 358  
 CGCTCTTTTAAAACTATTAGCCTTGTTTTACCGAGACAGAAAAACAAGTCGGGCATGCCTGTCATTTTGAGTAACTTCTCCGAAGATCGCTCACTGCTCTGTGTACATGCTAGATTTGTT 478  
 TATGTAAGTAAATACTCTATTCGTAAAATGAAATGTTTTTTGTGTTTGTATTTGCTCTGATAAATCACATTGATCAAGATTTATTCAGGGAACTGATAGCATTGTTCGAGGTTTATGCCA 598  
 AATATTTTAATGTATGAGGTCATAATTACGAAAATTATATATATATATGCTTAGAAAATGTGTATATAAGGGCTTGATGCGTTTTGCATAAGTTAATTACAGTAAGTCCGTGTTCCTAGG 718  
 TACAGCTTCATTACAATTATACTGAAAATCATTTAGGTCTCTTTCTGTCTGGGTTTTAAACCCATTCTAGCTTTTTTAGTGTAAAATAAAAATTAAAATGTGAAGAAATGAATTTTCATC 838  
 TTTTTTTTTAAAGGAACCAAAAACTACTGAGTACTCTCTGGCAAAGAAAGGTCAGAACTCCAAAGGAAACTTTTAAATTCAAAACAGACCGTAGCGACCCAAAGGAAAAAACAGACTGCG 958  
 ATTTATTTCAACTTTTTTTTTCGAAAATAGTTTCTTTGTATACTTCCTTAAGAATAAATAAGGAGCTCGGTAAGTGGACTCCATGTTACACTTAAAAATGTTTAAATAGATGCACCCACT 1078  
 GAATTAAAGTCGAAGGAGTCCCGCAAGAGCTCATAAAAAGACTTTTCGAGTTCCTCTCGCCTATTTTTCACAGCACAGATTTCAGCCTTTTTAAAACGTTTTAACAAAAAAAAGTTTGAG 1198  
 GCTAAGAGTCCGAATCAATCGTCATTTACAATTCTTAAAATTAAATGTTAAAACATTAACAAAATGGTGAGATGTTTCGTTACATATTCTTAGCTATTATAAATCTGAACTTTTAATTTT 1318  
 AAGCACATTAAACAGAAGGCTTTTCTTTTTTTTTTATTCGCTACCCCGAGATCTTTCCCCTCATTCACCCTGTGAGACTGTTGCACTGATAAAAATCGCCCAAGCAAGACAGTTTGAAAA 1438  
 CACACTTTCCGACTGTTTCCAAAGTCAGAGCTTAATTGTTACTTACGGCTTTCGCATGAGGTTTCTAAGCGCGGTGGGTCCTTATTTCTGCATTAACTCGAGTTTATTTTAAAAAAAATC 1558  
 AAATACAAGAAGAAAATAAACATCAGCAAATCGATAATTACTGTAACATCGCTCTCTCGCGCGCGGGGGGGGAAGCCGCCTGTATTTAATTACTCTATTCCACGTGAGATTGTTCTTAAT 1678  
 TATAATTTAAAAAGGCACCATAAGGTGAACAATTAATAAAAGAAAGGCTGTTTTCGTCGCTAAAACGGTACCAATCATAACGACAATATAGAGAAAGAATCACCCTATAATATTTAAACC 1798  
 GACGTGGCAGGCAGAGTGATTCTATGGATTAAATGAAGTAGGTAAAAGATAATATAAACTTGCCTTGTTGACTGAGGTTTATTTCTAATTATTACTCATCAATTACTTGATCCTCAAAGA 1918  
 CACTTAATTGCGCTGAACTCATCAGCTGGCCCGTTACCTCCTACAATTCTGCGCAGATTAGCCGGTAAATATTTAATTGCTAGGTGCGAGTTTGATCTCATTTACTCCCGTTTTAAACGC 2038  
 TGATGTGGAAAACTGTAGCAAAGGCCAGGAACAACTTAAATGTTCACTGCTTTGCCCACTAACTAATGATTAACTCGGCTGGGGTGGAAATCCGC                          2133
```

---

## >HOXB\_SHARK (912 bases)

```
 NO CONSERVED NODES FOUND  
GCCTCCTTTTTTATAGTTTGTCCCAAAGCAGACTGGGGTTAGTTCAAAAACTGCCTCAGATAGCACACATACACACACAGACAGAATGCACGGAGGAGCGGGTCCAGAGGAGCAGGACCT 120  
 CTGCGCCCTAAATGCGTCTGGGAGCAGAGACGCTGCAGCACATCAGCCGCTG||AAAAGTAAACGGTGGCCGGTCTTGGAGAATGGGACACCTGGAGCTTGCATTTTGGGGATTGACAGC 238  
 GGCGTGTCATCCACCCGTGCTCTGCGCTGCAGCTTCACAGGCGGAGAAATTAACCGGCAGGCTCCACTTTCAGAGGGTCCACAGACCTCTGCACGGCTGCGCAAGGTTTTCCTCCATCCT 358  
 CTTCTTCCTCTCTGTGACCTAAATGTTTTTAGTTTGTTCTTCTGTTCAAACAGTTACTCCGTCCGTTTGTCCTCTTCATCTGTACCATTCACTGCCGAGGAAGAGGAAGCTGTCGGGGAT 478  
 GAGTTCATCAGTTTGTTCTCCTTCTTCCACTTCATCCTCCGGTTCTGGAACCAGATCTTGATCTGGCGCTCCGTGAGACAGAGCGCATGGGCGATCTCGATGCGCCGCCGCCGCGTCAGG 598  
 TACCGGTTGAAGTGGAACTCCTTCTCCAGCTCCAGAGTCTGGTAGCGCGTGTACGTCTGCCGCCCGCGCCTGCCGGGGCTGCCGAAGGTGCCTGCGTGCGGGGACAGAAGTACAGAAGGA 718  
 AAGTTAACGTTTAACCCACCTCAAAACAAAAACTGTACGTGTTTGAGACAGAAACAAGCTAAGCCTGTATAATTTACAGTATTTCATTTTCTCTTTTATTTTATTTTATTTTTACCAGAC 838  
 GATGTATTTGCATTTAATAGAATAAATATTTATTTTAGATTGTGTTTAACATTTTTGCCGGTTGTGCAGTACTT                                               912
```

---
