# Supplementary material for: Highly conserved and cis-acting lncRNAs produced from paralogous regions in the center of HOXA and HOXB clusters in the endoderm lineage
Source: PLoS Genet. 2021 Jul 19;17(7):e1009681. doi: 10.1371/journal.pgen.1009681 (PMC8330917; doi:10.1371/journal.pgen.1009681)
Supplement: S1 Dataset — (ZIP) [file pgen.1009681.s015.zip › HOXB-AS3_var1/Html_Files/kmers_in_blocks.html]

 MOTIFS IN BLOCKS

# MOTIFS IN BLOCK DIAGRAMS

  

NAVIGATE ▼

▶HOXB-AS3 (depth:1)▶HOXB\_DOG\_ISOFORM1 (depth:2)▶HOXB5OS (depth:3)▶HOXB\_OPOSSUM (depth:4)▶HOXB\_XENOPUS (depth:5)▶HOXB\_COELACANTH\_HOXB (depth:6)▶HOXB\_GAR (depth:7)▶HOXB\_SHARK (depth:8)

  
  
  

## >HOXB-AS3 (573 bases)

```
gtcata

gtcata  
Depth:5 (HOXB_XENOPUS)  
Ei-value:Undefined, Pi-value:Undefined  
Er-value:0.000, Pr-value:0.000  
No matches to eCLIP DataNo matches to TargetScan


gcgacttt

gcgacttt  
Depth:5 (HOXB_XENOPUS)  
Ei-value:Undefined, Pi-value:Undefined  
Er-value:0.000, Pr-value:0.000  
No matches to eCLIP DataNo matches to TargetScan


tggg

gtcatagcgacttttggg  
Depth:4 (HOXB_OPOSSUM)  
Ei-value:Undefined, Pi-value:Undefined  
Er-value:0.000, Pr-value:0.000  
No matches to eCLIP DataNo matches to TargetScan


a

gtcatagcgacttttgggatagtttgctat  
Depth:2 (HOXB_DOG_ISOFORM1)  
Ei-value:Undefined, Pi-value:Undefined  
Er-value:0.000, Pr-value:0.000  
No matches to eCLIP DataNo matches to TargetScan


tagtttgct

tagtttgct  
Depth:4 (HOXB_OPOSSUM)  
Ei-value:Undefined, Pi-value:Undefined  
Er-value:0.000, Pr-value:0.000  
No matches to eCLIP DataNo matches to TargetScan


at

gtcatagcgacttttgggatagtttgctat  
Depth:2 (HOXB_DOG_ISOFORM1)  
Ei-value:Undefined, Pi-value:Undefined  
Er-value:0.000, Pr-value:0.000  
No matches to eCLIP DataNo matches to TargetScan

-

ga

gacaaaggg  
Depth:2 (HOXB_DOG_ISOFORM1)  
Ei-value:Undefined, Pi-value:Undefined  
Er-value:0.000, Pr-value:0.000  
No matches to eCLIP DataNo matches to TargetScan


caaaggg

caaaggg  
Depth:4 (HOXB_OPOSSUM)  
Ei-value:Undefined, Pi-value:Undefined  
Er-value:0.000, Pr-value:0.000  
No matches to eCLIP DataNo matches to TargetScan

-

gacaaagtca

gacaaagtca  
Depth:3 (HOXB5OS)  
Ei-value:Undefined, Pi-value:Undefined  
Er-value:0.000, Pr-value:0.000  
No matches to eCLIP DataNo matches to TargetScan


agggg

gacaaagtcaagggg  
Depth:2 (HOXB_DOG_ISOFORM1)  
Ei-value:Undefined, Pi-value:Undefined  
Er-value:0.000, Pr-value:0.000  
No matches to eCLIP DataNo matches to TargetScan

--------

aaggagg

aaggagg  
Depth:3 (HOXB5OS)  
Ei-value:Undefined, Pi-value:Undefined  
Er-value:0.000, Pr-value:0.000  
No matches to eCLIP DataNo matches to TargetScan


gcc

aaggagggcc  
Depth:2 (HOXB_DOG_ISOFORM1)  
Ei-value:Undefined, Pi-value:Undefined  
Er-value:0.000, Pr-value:0.000  
No matches to eCLIP DataNo matches to TargetScan

-

agtag

agtagagcctc  
Depth:2 (HOXB_DOG_ISOFORM1)  
Ei-value:Undefined, Pi-value:Undefined  
Er-value:0.000, Pr-value:0.000  
No matches to eCLIP DataMATCHES To TargetScan▶ miR-485-5p:GAGGCUG▶ miR-760:GGCUCUG


agcctc

agcctc  
Depth:3 (HOXB5OS)  
Ei-value:Undefined, Pi-value:Undefined  
Er-value:0.000, Pr-value:0.000  
No matches to eCLIP DataMATCHES To TargetScan▶ miR-485-5p:GAGGCUG

----------------

ct

ctcctcaccagctcccc  
Depth:2 (HOXB_DOG_ISOFORM1)  
Ei-value:Undefined, Pi-value:Undefined  
Er-value:0.000, Pr-value:0.000  
No matches to eCLIP DataMATCHES To TargetScan▶ miR-1224-5p:UGAGGAC▶ miR-138-5p:GCUGGUG


cctcacca

cctcacca  
Depth:3 (HOXB5OS)  
Ei-value:Undefined, Pi-value:Undefined  
Er-value:0.000, Pr-value:0.000  
No matches to eCLIP DataNo matches to TargetScan


g

gctcccc  
Depth:3 (HOXB5OS)  
Ei-value:Undefined, Pi-value:Undefined  
Er-value:0.000, Pr-value:0.000  
No matches to eCLIP DataNo matches to TargetScan


ctcccc

ctcccc  
Depth:4 (HOXB_OPOSSUM)  
Ei-value:Undefined, Pi-value:Undefined  
Er-value:0.010, Pr-value:0.000  
No matches to eCLIP DataNo matches to TargetScan

- 120  
 -----

ccaagtcc

ccaagtcc  
Depth:2 (HOXB_DOG_ISOFORM1)  
Ei-value:Undefined, Pi-value:Undefined  
Er-value:0.000, Pr-value:0.000  
No matches to eCLIP DataNo matches to TargetScan

-

gtaagaagtt

gtaagaagtt  
Depth:4 (HOXB_OPOSSUM)  
Ei-value:Undefined, Pi-value:Undefined  
Er-value:0.000, Pr-value:0.000  
No matches to eCLIP DataNo matches to TargetScan


gggcc

gtaagaagttgggcc  
Depth:3 (HOXB5OS)  
Ei-value:Undefined, Pi-value:Undefined  
Er-value:0.000, Pr-value:0.000  
No matches to eCLIP DataNo matches to TargetScan


a

gtaagaagttgggccaagctggaagggattgaccggccg  
Depth:2 (HOXB_DOG_ISOFORM1)  
Ei-value:Undefined, Pi-value:Undefined  
Er-value:0.000, Pr-value:0.000  
No matches to eCLIP DataMATCHES To TargetScan▶ miR-188-5p:AUCCCUU▶ miR-204-5p/211-5p:UCCCUUU▶ miR-328-3p:UGGCCCU


agctg

agctggaagggattgaccg  
Depth:3 (HOXB5OS)  
Ei-value:Undefined, Pi-value:Undefined  
Er-value:0.000, Pr-value:0.000  
No matches to eCLIP DataMATCHES To TargetScan▶ miR-188-5p:AUCCCUU▶ miR-204-5p/211-5p:UCCCUUU


gaaggga

gaaggga  
Depth:4 (HOXB_OPOSSUM)  
Ei-value:Undefined, Pi-value:Undefined  
Er-value:0.000, Pr-value:0.000  
No matches to eCLIP DataMATCHES To TargetScan▶ miR-204-5p/211-5p:UCCCUUU


ttgaccg

agctggaagggattgaccg  
Depth:3 (HOXB5OS)  
Ei-value:Undefined, Pi-value:Undefined  
Er-value:0.000, Pr-value:0.000  
No matches to eCLIP DataMATCHES To TargetScan▶ miR-188-5p:AUCCCUU▶ miR-204-5p/211-5p:UCCCUUU


g||ccg

gtaagaagttgggccaagctggaagggattgaccggccg  
Depth:2 (HOXB_DOG_ISOFORM1)  
Ei-value:Undefined, Pi-value:Undefined  
Er-value:0.000, Pr-value:0.000  
No matches to eCLIP DataMATCHES To TargetScan▶ miR-188-5p:AUCCCUU▶ miR-204-5p/211-5p:UCCCUUU▶ miR-328-3p:UGGCCCU

---------

cctcgcc

cctcgcc  
Depth:2 (HOXB_DOG_ISOFORM1)  
Ei-value:Undefined, Pi-value:Undefined  
Er-value:0.000, Pr-value:0.000  
No matches to eCLIP DataNo matches to TargetScan


ggcctc

ggcctc  
Depth:4 (HOXB_OPOSSUM)  
Ei-value:Undefined, Pi-value:Undefined  
Er-value:0.010, Pr-value:0.000  
No matches to eCLIP DataNo matches to TargetScan

-

gcggagat

gcggagattccaggccc  
Depth:3 (HOXB5OS)  
Ei-value:Undefined, Pi-value:Undefined  
Er-value:0.000, Pr-value:0.000  
No matches to eCLIP DataMATCHES To TargetScan▶ miR-216a-5p:AAUCUCA▶ miR-216b-5p:AAUCUCU


tccaggc

tccaggc  
Depth:4 (HOXB_OPOSSUM)  
Ei-value:Undefined, Pi-value:Undefined  
Er-value:0.000, Pr-value:0.000  
No matches to eCLIP DataNo matches to TargetScan


cc

gcggagattccaggccc  
Depth:3 (HOXB5OS)  
Ei-value:Undefined, Pi-value:Undefined  
Er-value:0.000, Pr-value:0.000  
No matches to eCLIP DataMATCHES To TargetScan▶ miR-216a-5p:AAUCUCA▶ miR-216b-5p:AAUCUCU


t

gcggagattccaggccct  
Depth:2 (HOXB_DOG_ISOFORM1)  
Ei-value:Undefined, Pi-value:Undefined  
Er-value:0.000, Pr-value:0.000  
No matches to eCLIP DataMATCHES To TargetScan▶ miR-216a-5p:AAUCUCA▶ miR-216b-5p:AAUCUCU

----------

ggacgtccct

ggacgtccct  
Depth:2 (HOXB_DOG_ISOFORM1)  
Ei-value:Undefined, Pi-value:Undefined  
Er-value:0.000, Pr-value:0.000  
No matches to eCLIP DataNo matches to TargetScan

-

agc

agcgccaccgcc  
Depth:3 (HOXB5OS)  
Ei-value:Undefined, Pi-value:Undefined  
Er-value:0.000, Pr-value:0.000  
No matches to eCLIP DataNo matches to TargetScan

 238  


agcgccaccgcc  
Depth:3 (HOXB5OS)  
Ei-value:Undefined, Pi-value:Undefined  
Er-value:0.000, Pr-value:0.000  
No matches to eCLIP DataNo matches to TargetScan


gccacc

gccacc  
Depth:5 (HOXB_XENOPUS)  
Ei-value:Undefined, Pi-value:Undefined  
Er-value:0.000, Pr-value:0.000  
No matches to eCLIP DataNo matches to TargetScan


gcc

agcgccaccgcc  
Depth:3 (HOXB5OS)  
Ei-value:Undefined, Pi-value:Undefined  
Er-value:0.000, Pr-value:0.000  
No matches to eCLIP DataNo matches to TargetScan

----------------------------------

ccgcacc

ccgcacc  
Depth:2 (HOXB_DOG_ISOFORM1)  
Ei-value:Undefined, Pi-value:Undefined  
Er-value:0.000, Pr-value:0.000  
No matches to eCLIP DataNo matches to TargetScan

--||------------

caggctgc

caggctgc  
Depth:2 (HOXB_DOG_ISOFORM1)  
Ei-value:Undefined, Pi-value:Undefined  
Er-value:0.000, Pr-value:0.000  
No matches to eCLIP DataNo matches to TargetScan

----

ggcggcgc

ggcggcgc  
Depth:2 (HOXB_DOG_ISOFORM1)  
Ei-value:Undefined, Pi-value:Undefined  
Er-value:0.000, Pr-value:0.000  
No matches to eCLIP DataNo matches to TargetScan

-------------------------------

ccg

ccgggc  
Depth:2 (HOXB_DOG_ISOFORM1)  
Ei-value:Undefined, Pi-value:Undefined  
Er-value:0.090, Pr-value:0.010  
No matches to eCLIP DataNo matches to TargetScan

 356  


ggc

ccgggc  
Depth:2 (HOXB_DOG_ISOFORM1)  
Ei-value:Undefined, Pi-value:Undefined  
Er-value:0.090, Pr-value:0.010  
No matches to eCLIP DataNo matches to TargetScan

--------------------

gagcg

gagcggccgggatgcggccacacc  
Depth:2 (HOXB_DOG_ISOFORM1)  
Ei-value:Undefined, Pi-value:Undefined  
Er-value:0.000, Pr-value:0.000  
No matches to eCLIP DataMATCHES To TargetScan▶ miR-324-5p:GCAUCCC


gccggga

gccggga  
Depth:3 (HOXB5OS)  
Ei-value:Undefined, Pi-value:Undefined  
Er-value:0.000, Pr-value:0.000  
No matches to eCLIP DataNo matches to TargetScan


tgcgg

gagcggccgggatgcggccacacc  
Depth:2 (HOXB_DOG_ISOFORM1)  
Ei-value:Undefined, Pi-value:Undefined  
Er-value:0.000, Pr-value:0.000  
No matches to eCLIP DataMATCHES To TargetScan▶ miR-324-5p:GCAUCCC


ccacac

ccacac  
Depth:3 (HOXB5OS)  
Ei-value:Undefined, Pi-value:Undefined  
Er-value:0.000, Pr-value:0.000  
No matches to eCLIP DataNo matches to TargetScan


c

gagcggccgggatgcggccacacc  
Depth:2 (HOXB_DOG_ISOFORM1)  
Ei-value:Undefined, Pi-value:Undefined  
Er-value:0.000, Pr-value:0.000  
No matches to eCLIP DataMATCHES To TargetScan▶ miR-324-5p:GCAUCCC

-----

gg

ggtaaact  
Depth:3 (HOXB5OS)  
Ei-value:Undefined, Pi-value:Undefined  
Er-value:0.000, Pr-value:0.000  
No matches to eCLIP DataNo matches to TargetScan


taaact

taaact  
Depth:5 (HOXB_XENOPUS)  
Ei-value:Undefined, Pi-value:Undefined  
Er-value:0.000, Pr-value:0.000  
No matches to eCLIP DataNo matches to TargetScan

-------------------------------------

tccccac

tccccac  
Depth:2 (HOXB_DOG_ISOFORM1)  
Ei-value:Undefined, Pi-value:Undefined  
Er-value:0.000, Pr-value:0.000  
No matches to eCLIP DataMATCHES To TargetScan▶ miR-491-5p:GUGGGGA

--------------

tt

ttttatttgg  
Depth:2 (HOXB_DOG_ISOFORM1)  
Ei-value:Undefined, Pi-value:Undefined  
Er-value:0.000, Pr-value:0.000  
No matches to eCLIP DataNo matches to TargetScan

 476  


ttatttgg

ttttatttgg  
Depth:2 (HOXB_DOG_ISOFORM1)  
Ei-value:Undefined, Pi-value:Undefined  
Er-value:0.000, Pr-value:0.000  
No matches to eCLIP DataNo matches to TargetScan

------------

aatttagaa

aatttagaa  
Depth:3 (HOXB5OS)  
Ei-value:Undefined, Pi-value:Undefined  
Er-value:0.000, Pr-value:0.000  
No matches to eCLIP DataNo matches to TargetScan

-

gagataaa

gagataaa  
Depth:2 (HOXB_DOG_ISOFORM1)  
Ei-value:Undefined, Pi-value:Undefined  
Er-value:0.000, Pr-value:0.000  
No matches to eCLIP DataNo matches to TargetScan

-----------------------------------------------------------                        573
```

---

## >HOXB\_DOG\_ISOFORM1 (2741 bases)

```
 --------------------------------------------------------------------------------

gtcata

gtcata  
Depth:5 (HOXB_XENOPUS)  
Ei-value:Undefined, Pi-value:Undefined  
Er-value:0.000, Pr-value:0.000  
No matches to TargetScan


gcgacttt

gcgacttt  
Depth:5 (HOXB_XENOPUS)  
Ei-value:Undefined, Pi-value:Undefined  
Er-value:0.000, Pr-value:0.000  
No matches to TargetScan


tggg

gtcatagcgacttttggg  
Depth:4 (HOXB_OPOSSUM)  
Ei-value:Undefined, Pi-value:Undefined  
Er-value:0.000, Pr-value:0.000  
No matches to TargetScan


a

gtcatagcgacttttgggatagtttgctat  
Depth:2 (HOXB_DOG_ISOFORM1)  
Ei-value:Undefined, Pi-value:Undefined  
Er-value:0.000, Pr-value:0.000  
No matches to TargetScan


tagtttgct

tagtttgct  
Depth:4 (HOXB_OPOSSUM)  
Ei-value:Undefined, Pi-value:Undefined  
Er-value:0.000, Pr-value:0.000  
No matches to TargetScan


at

gtcatagcgacttttgggatagtttgctat  
Depth:2 (HOXB_DOG_ISOFORM1)  
Ei-value:Undefined, Pi-value:Undefined  
Er-value:0.000, Pr-value:0.000  
No matches to TargetScan

-

ga

gacaaaggg  
Depth:2 (HOXB_DOG_ISOFORM1)  
Ei-value:Undefined, Pi-value:Undefined  
Er-value:0.000, Pr-value:0.000  
No matches to TargetScan


caaaggg

caaaggg  
Depth:4 (HOXB_OPOSSUM)  
Ei-value:Undefined, Pi-value:Undefined  
Er-value:0.000, Pr-value:0.000  
No matches to TargetScan

 120  


caaaggg  
Depth:4 (HOXB_OPOSSUM)  
Ei-value:Undefined, Pi-value:Undefined  
Er-value:0.000, Pr-value:0.000  
No matches to TargetScan

-

gacaaagtca

gacaaagtca  
Depth:3 (HOXB5OS)  
Ei-value:Undefined, Pi-value:Undefined  
Er-value:0.000, Pr-value:0.000  
No matches to TargetScan


agggg

gacaaagtcaagggg  
Depth:2 (HOXB_DOG_ISOFORM1)  
Ei-value:Undefined, Pi-value:Undefined  
Er-value:0.000, Pr-value:0.000  
No matches to TargetScan

--------

aaggagg

aaggagg  
Depth:3 (HOXB5OS)  
Ei-value:Undefined, Pi-value:Undefined  
Er-value:0.000, Pr-value:0.000  
No matches to TargetScan


gcc

aaggagggcc  
Depth:2 (HOXB_DOG_ISOFORM1)  
Ei-value:Undefined, Pi-value:Undefined  
Er-value:0.000, Pr-value:0.000  
No matches to TargetScan

-

agtag

agtagagcctc  
Depth:2 (HOXB_DOG_ISOFORM1)  
Ei-value:Undefined, Pi-value:Undefined  
Er-value:0.000, Pr-value:0.000  
MATCHES To TargetScan▶ miR-485-5p:GAGGCUG▶ miR-760:GGCUCUG


agcctc

agcctc  
Depth:3 (HOXB5OS)  
Ei-value:Undefined, Pi-value:Undefined  
Er-value:0.000, Pr-value:0.000  
MATCHES To TargetScan▶ miR-485-5p:GAGGCUG

----------------

ct

ctcctcaccagctcccc  
Depth:2 (HOXB_DOG_ISOFORM1)  
Ei-value:Undefined, Pi-value:Undefined  
Er-value:0.000, Pr-value:0.000  
MATCHES To TargetScan▶ miR-1224-5p:UGAGGAC▶ miR-138-5p:GCUGGUG


cctcacca

cctcacca  
Depth:3 (HOXB5OS)  
Ei-value:Undefined, Pi-value:Undefined  
Er-value:0.000, Pr-value:0.000  
No matches to TargetScan


g

gctcccc  
Depth:3 (HOXB5OS)  
Ei-value:Undefined, Pi-value:Undefined  
Er-value:0.000, Pr-value:0.000  
No matches to TargetScan


ctcccc

ctcccc  
Depth:4 (HOXB_OPOSSUM)  
Ei-value:Undefined, Pi-value:Undefined  
Er-value:0.010, Pr-value:0.000  
No matches to TargetScan

------

ccaagtcc

ccaagtcc  
Depth:2 (HOXB_DOG_ISOFORM1)  
Ei-value:Undefined, Pi-value:Undefined  
Er-value:0.000, Pr-value:0.000  
No matches to TargetScan

-

gtaagaagtt

gtaagaagtt  
Depth:4 (HOXB_OPOSSUM)  
Ei-value:Undefined, Pi-value:Undefined  
Er-value:0.000, Pr-value:0.000  
No matches to TargetScan


gggcc

gtaagaagttgggcc  
Depth:3 (HOXB5OS)  
Ei-value:Undefined, Pi-value:Undefined  
Er-value:0.000, Pr-value:0.000  
No matches to TargetScan


a

gtaagaagttgggccaagctggaagggattgaccggccg  
Depth:2 (HOXB_DOG_ISOFORM1)  
Ei-value:Undefined, Pi-value:Undefined  
Er-value:0.000, Pr-value:0.000  
MATCHES To TargetScan▶ miR-188-5p:AUCCCUU▶ miR-204-5p/211-5p:UCCCUUU▶ miR-328-3p:UGGCCCU


agctg

agctggaagggattgaccg  
Depth:3 (HOXB5OS)  
Ei-value:Undefined, Pi-value:Undefined  
Er-value:0.000, Pr-value:0.000  
MATCHES To TargetScan▶ miR-188-5p:AUCCCUU▶ miR-204-5p/211-5p:UCCCUUU


gaagg

gaaggga  
Depth:4 (HOXB_OPOSSUM)  
Ei-value:Undefined, Pi-value:Undefined  
Er-value:0.000, Pr-value:0.000  
MATCHES To TargetScan▶ miR-204-5p/211-5p:UCCCUUU

 240  


ga

gaaggga  
Depth:4 (HOXB_OPOSSUM)  
Ei-value:Undefined, Pi-value:Undefined  
Er-value:0.000, Pr-value:0.000  
MATCHES To TargetScan▶ miR-204-5p/211-5p:UCCCUUU


ttgaccg

agctggaagggattgaccg  
Depth:3 (HOXB5OS)  
Ei-value:Undefined, Pi-value:Undefined  
Er-value:0.000, Pr-value:0.000  
MATCHES To TargetScan▶ miR-188-5p:AUCCCUU▶ miR-204-5p/211-5p:UCCCUUU


g||ccg

gtaagaagttgggccaagctggaagggattgaccggccg  
Depth:2 (HOXB_DOG_ISOFORM1)  
Ei-value:Undefined, Pi-value:Undefined  
Er-value:0.000, Pr-value:0.000  
MATCHES To TargetScan▶ miR-188-5p:AUCCCUU▶ miR-204-5p/211-5p:UCCCUUU▶ miR-328-3p:UGGCCCU

------

cctcgcc

cctcgcc  
Depth:2 (HOXB_DOG_ISOFORM1)  
Ei-value:Undefined, Pi-value:Undefined  
Er-value:0.000, Pr-value:0.000  
No matches to TargetScan

------

ggcctc

ggcctc  
Depth:4 (HOXB_OPOSSUM)  
Ei-value:Undefined, Pi-value:Undefined  
Er-value:0.010, Pr-value:0.000  
No matches to TargetScan

-

gcggagat

gcggagattccaggccc  
Depth:3 (HOXB5OS)  
Ei-value:Undefined, Pi-value:Undefined  
Er-value:0.000, Pr-value:0.000  
MATCHES To TargetScan▶ miR-216a-5p:AAUCUCA▶ miR-216b-5p:AAUCUCU


tccaggc

tccaggc  
Depth:4 (HOXB_OPOSSUM)  
Ei-value:Undefined, Pi-value:Undefined  
Er-value:0.000, Pr-value:0.000  
No matches to TargetScan


cc

gcggagattccaggccc  
Depth:3 (HOXB5OS)  
Ei-value:Undefined, Pi-value:Undefined  
Er-value:0.000, Pr-value:0.000  
MATCHES To TargetScan▶ miR-216a-5p:AAUCUCA▶ miR-216b-5p:AAUCUCU


t

gcggagattccaggccct  
Depth:2 (HOXB_DOG_ISOFORM1)  
Ei-value:Undefined, Pi-value:Undefined  
Er-value:0.000, Pr-value:0.000  
MATCHES To TargetScan▶ miR-216a-5p:AAUCUCA▶ miR-216b-5p:AAUCUCU

----------

ggacgtccct

ggacgtccct  
Depth:2 (HOXB_DOG_ISOFORM1)  
Ei-value:Undefined, Pi-value:Undefined  
Er-value:0.000, Pr-value:0.000  
No matches to TargetScan

-

agc

agcgccaccgcc  
Depth:3 (HOXB5OS)  
Ei-value:Undefined, Pi-value:Undefined  
Er-value:0.000, Pr-value:0.000  
No matches to TargetScan


gccacc

gccacc  
Depth:5 (HOXB_XENOPUS)  
Ei-value:Undefined, Pi-value:Undefined  
Er-value:0.000, Pr-value:0.000  
No matches to TargetScan


gcc

agcgccaccgcc  
Depth:3 (HOXB5OS)  
Ei-value:Undefined, Pi-value:Undefined  
Er-value:0.000, Pr-value:0.000  
No matches to TargetScan

---------------------------- 358  
 -----

ccgcacc

ccgcacc  
Depth:2 (HOXB_DOG_ISOFORM1)  
Ei-value:Undefined, Pi-value:Undefined  
Er-value:0.000, Pr-value:0.000  
No matches to TargetScan

------------------------------------------------------------------------------------------------------------ 478  
 ------------------------------------------------------------------------------------------------------------------------ 598  
 ------------------------------------------------------------------------------------------------------------------------ 718  
 ------------------------------------------------------------------------------------------------------------------------ 838  
 ------------------------------------------------------------------------------------------------------------------------ 958  
 -----------------------------------------------

caggctgc

caggctgc  
Depth:2 (HOXB_DOG_ISOFORM1)  
Ei-value:Undefined, Pi-value:Undefined  
Er-value:0.000, Pr-value:0.000  
No matches to TargetScan

----------------------------------------------------------------- 1078  
 ------------------------------------------------------------------------------------------------------------------------ 1198  
 ------------------------------------------------------------------------------------------------------------------------ 1318  
 ------------------------------------------------------------------------------------------------------------------------ 1438  
 ------------------------------------------------------------------------------------------------------------------------ 1558  
 ------------------------------------------------------------------------------------------------------------------------ 1678  
 ------------------------------------------------------------------------------------------------------------------------ 1798  
 ------------------------------------------------------------------------------------------------------------------------ 1918  
 ------------------------------------------------------------------------------------------------------------------------ 2038  
 ------------------------------------------------------------------------------------------------------------------------ 2158  
 ------------------------------------------------------------------------------------------------------------------------ 2278  
 ------------------------------------------------------------------------------------------------------------------------ 2398  
 ------------------------------------------------------------------------------------------------------------------------ 2518  
 --------------------------------------------------------------------

ggcggcgc

ggcggcgc  
Depth:2 (HOXB_DOG_ISOFORM1)  
Ei-value:Undefined, Pi-value:Undefined  
Er-value:0.000, Pr-value:0.000  
No matches to TargetScan

--

ccgggc

ccgggc  
Depth:2 (HOXB_DOG_ISOFORM1)  
Ei-value:Undefined, Pi-value:Undefined  
Er-value:0.090, Pr-value:0.010  
No matches to TargetScan

---

gagcg

gagcggccgggatgcggccacacc  
Depth:2 (HOXB_DOG_ISOFORM1)  
Ei-value:Undefined, Pi-value:Undefined  
Er-value:0.000, Pr-value:0.000  
MATCHES To TargetScan▶ miR-324-5p:GCAUCCC


gccggga

gccggga  
Depth:3 (HOXB5OS)  
Ei-value:Undefined, Pi-value:Undefined  
Er-value:0.000, Pr-value:0.000  
No matches to TargetScan


tgcgg

gagcggccgggatgcggccacacc  
Depth:2 (HOXB_DOG_ISOFORM1)  
Ei-value:Undefined, Pi-value:Undefined  
Er-value:0.000, Pr-value:0.000  
MATCHES To TargetScan▶ miR-324-5p:GCAUCCC


ccacac

ccacac  
Depth:3 (HOXB5OS)  
Ei-value:Undefined, Pi-value:Undefined  
Er-value:0.000, Pr-value:0.000  
No matches to TargetScan


c

gagcggccgggatgcggccacacc  
Depth:2 (HOXB_DOG_ISOFORM1)  
Ei-value:Undefined, Pi-value:Undefined  
Er-value:0.000, Pr-value:0.000  
MATCHES To TargetScan▶ miR-324-5p:GCAUCCC

-----

gg

ggtaaact  
Depth:3 (HOXB5OS)  
Ei-value:Undefined, Pi-value:Undefined  
Er-value:0.000, Pr-value:0.000  
No matches to TargetScan


ta

taaact  
Depth:5 (HOXB_XENOPUS)  
Ei-value:Undefined, Pi-value:Undefined  
Er-value:0.000, Pr-value:0.000  
No matches to TargetScan

 2638  


aact

taaact  
Depth:5 (HOXB_XENOPUS)  
Ei-value:Undefined, Pi-value:Undefined  
Er-value:0.000, Pr-value:0.000  
No matches to TargetScan

----------------------------------------------

tccccac

tccccac  
Depth:2 (HOXB_DOG_ISOFORM1)  
Ei-value:Undefined, Pi-value:Undefined  
Er-value:0.000, Pr-value:0.000  
MATCHES To TargetScan▶ miR-491-5p:GUGGGGA

-------

ttttatttgg

ttttatttgg  
Depth:2 (HOXB_DOG_ISOFORM1)  
Ei-value:Undefined, Pi-value:Undefined  
Er-value:0.000, Pr-value:0.000  
No matches to TargetScan

-----------

aatttagaa

aatttagaa  
Depth:3 (HOXB5OS)  
Ei-value:Undefined, Pi-value:Undefined  
Er-value:0.000, Pr-value:0.000  
No matches to TargetScan

-

gagataaa

gagataaa  
Depth:2 (HOXB_DOG_ISOFORM1)  
Ei-value:Undefined, Pi-value:Undefined  
Er-value:0.000, Pr-value:0.000  
No matches to TargetScan

2741
```

---

## >HOXB5OS (596 bases)

```
 --

gtcata

gtcata  
Depth:5 (HOXB_XENOPUS)  
Ei-value:Undefined, Pi-value:Undefined  
Er-value:0.000, Pr-value:0.000  
No matches to TargetScan


gcgacttt

gcgacttt  
Depth:5 (HOXB_XENOPUS)  
Ei-value:Undefined, Pi-value:Undefined  
Er-value:0.000, Pr-value:0.000  
No matches to TargetScan


tggg

gtcatagcgacttttggg  
Depth:4 (HOXB_OPOSSUM)  
Ei-value:Undefined, Pi-value:Undefined  
Er-value:0.000, Pr-value:0.000  
No matches to TargetScan

-

tagtttgct

tagtttgct  
Depth:4 (HOXB_OPOSSUM)  
Ei-value:Undefined, Pi-value:Undefined  
Er-value:0.000, Pr-value:0.000  
No matches to TargetScan

-----

caaaggg

caaaggg  
Depth:4 (HOXB_OPOSSUM)  
Ei-value:Undefined, Pi-value:Undefined  
Er-value:0.000, Pr-value:0.000  
No matches to TargetScan

-

gacaaagtca

gacaaagtca  
Depth:3 (HOXB5OS)  
Ei-value:Undefined, Pi-value:Undefined  
Er-value:0.000, Pr-value:0.000  
No matches to TargetScan

-------------

aaggagg

aaggagg  
Depth:3 (HOXB5OS)  
Ei-value:Undefined, Pi-value:Undefined  
Er-value:0.000, Pr-value:0.000  
No matches to TargetScan

---------

agcctc

agcctc  
Depth:3 (HOXB5OS)  
Ei-value:Undefined, Pi-value:Undefined  
Er-value:0.000, Pr-value:0.000  
MATCHES To TargetScan▶ miR-485-5p:GAGGCUG

------

cctcacca

cctcacca  
Depth:3 (HOXB5OS)  
Ei-value:Undefined, Pi-value:Undefined  
Er-value:0.000, Pr-value:0.000  
No matches to TargetScan

-----------

g

gctcccc  
Depth:3 (HOXB5OS)  
Ei-value:Undefined, Pi-value:Undefined  
Er-value:0.000, Pr-value:0.000  
No matches to TargetScan


ctcccc

ctcccc  
Depth:4 (HOXB_OPOSSUM)  
Ei-value:Undefined, Pi-value:Undefined  
Er-value:0.010, Pr-value:0.000  
No matches to TargetScan

 120  


ctcccc  
Depth:4 (HOXB_OPOSSUM)  
Ei-value:Undefined, Pi-value:Undefined  
Er-value:0.010, Pr-value:0.000  
No matches to TargetScan

-------------------

gtaagaagtt

gtaagaagtt  
Depth:4 (HOXB_OPOSSUM)  
Ei-value:Undefined, Pi-value:Undefined  
Er-value:0.000, Pr-value:0.000  
No matches to TargetScan


gggcc

gtaagaagttgggcc  
Depth:3 (HOXB5OS)  
Ei-value:Undefined, Pi-value:Undefined  
Er-value:0.000, Pr-value:0.000  
No matches to TargetScan

-

agctg

agctggaagggattgaccg  
Depth:3 (HOXB5OS)  
Ei-value:Undefined, Pi-value:Undefined  
Er-value:0.000, Pr-value:0.000  
MATCHES To TargetScan▶ miR-188-5p:AUCCCUU▶ miR-204-5p/211-5p:UCCCUUU


gaaggga

gaaggga  
Depth:4 (HOXB_OPOSSUM)  
Ei-value:Undefined, Pi-value:Undefined  
Er-value:0.000, Pr-value:0.000  
MATCHES To TargetScan▶ miR-204-5p/211-5p:UCCCUUU


ttgaccg||

agctggaagggattgaccg  
Depth:3 (HOXB5OS)  
Ei-value:Undefined, Pi-value:Undefined  
Er-value:0.000, Pr-value:0.000  
MATCHES To TargetScan▶ miR-188-5p:AUCCCUU▶ miR-204-5p/211-5p:UCCCUUU

--------------------

ggcctc

ggcctc  
Depth:4 (HOXB_OPOSSUM)  
Ei-value:Undefined, Pi-value:Undefined  
Er-value:0.010, Pr-value:0.000  
No matches to TargetScan

-

gcggagat

gcggagattccaggccc  
Depth:3 (HOXB5OS)  
Ei-value:Undefined, Pi-value:Undefined  
Er-value:0.000, Pr-value:0.000  
MATCHES To TargetScan▶ miR-216a-5p:AAUCUCA▶ miR-216b-5p:AAUCUCU


tccaggc

tccaggc  
Depth:4 (HOXB_OPOSSUM)  
Ei-value:Undefined, Pi-value:Undefined  
Er-value:0.000, Pr-value:0.000  
No matches to TargetScan


cc

gcggagattccaggccc  
Depth:3 (HOXB5OS)  
Ei-value:Undefined, Pi-value:Undefined  
Er-value:0.000, Pr-value:0.000  
MATCHES To TargetScan▶ miR-216a-5p:AAUCUCA▶ miR-216b-5p:AAUCUCU

-------------------- 238  
 --

agc

agcgccaccgcc  
Depth:3 (HOXB5OS)  
Ei-value:Undefined, Pi-value:Undefined  
Er-value:0.000, Pr-value:0.000  
No matches to TargetScan


gccacc

gccacc  
Depth:5 (HOXB_XENOPUS)  
Ei-value:Undefined, Pi-value:Undefined  
Er-value:0.000, Pr-value:0.000  
No matches to TargetScan


gcc

agcgccaccgcc  
Depth:3 (HOXB5OS)  
Ei-value:Undefined, Pi-value:Undefined  
Er-value:0.000, Pr-value:0.000  
No matches to TargetScan

---------------

gccggga

gccggga  
Depth:3 (HOXB5OS)  
Ei-value:Undefined, Pi-value:Undefined  
Er-value:0.000, Pr-value:0.000  
No matches to TargetScan

------------

ccacac

ccacac  
Depth:3 (HOXB5OS)  
Ei-value:Undefined, Pi-value:Undefined  
Er-value:0.000, Pr-value:0.000  
No matches to TargetScan

--------------------------------------------------------------||-- 356  
 ------------------------------------------------------------------------------------------------

gg

ggtaaact  
Depth:3 (HOXB5OS)  
Ei-value:Undefined, Pi-value:Undefined  
Er-value:0.000, Pr-value:0.000  
No matches to TargetScan


taaact

taaact  
Depth:5 (HOXB_XENOPUS)  
Ei-value:Undefined, Pi-value:Undefined  
Er-value:0.000, Pr-value:0.000  
No matches to TargetScan

---------------- 476  
 -----------------------------------------------

aatttagaa

aatttagaa  
Depth:3 (HOXB5OS)  
Ei-value:Undefined, Pi-value:Undefined  
Er-value:0.000, Pr-value:0.000  
No matches to TargetScan

---------------------------------------------------------------- 596  
                                                                                                                          596
```

---

## >HOXB\_OPOSSUM (1381 bases)

```
 ------------------------------------------------------------------------------------------------------------------------ 120  
 ------------------------------------------------------------------------------------------------------------------------ 240  
 ------------------------------------------------------------------------------------------------------------------------ 360  
 ---------------------------------------------------------

gtcata

gtcata  
Depth:5 (HOXB_XENOPUS)  
Ei-value:Undefined, Pi-value:Undefined  
Er-value:0.000, Pr-value:0.000  
No matches to TargetScan


gcgacttt

gcgacttt  
Depth:5 (HOXB_XENOPUS)  
Ei-value:Undefined, Pi-value:Undefined  
Er-value:0.000, Pr-value:0.000  
No matches to TargetScan


tggg

gtcatagcgacttttggg  
Depth:4 (HOXB_OPOSSUM)  
Ei-value:Undefined, Pi-value:Undefined  
Er-value:0.000, Pr-value:0.000  
No matches to TargetScan

-

tagtttgct

tagtttgct  
Depth:4 (HOXB_OPOSSUM)  
Ei-value:Undefined, Pi-value:Undefined  
Er-value:0.000, Pr-value:0.000  
No matches to TargetScan

----------------------------------- 480  


caaaggg

caaaggg  
Depth:4 (HOXB_OPOSSUM)  
Ei-value:Undefined, Pi-value:Undefined  
Er-value:0.000, Pr-value:0.000  
No matches to TargetScan

----------------------------------------------

ctcccc

ctcccc  
Depth:4 (HOXB_OPOSSUM)  
Ei-value:Undefined, Pi-value:Undefined  
Er-value:0.010, Pr-value:0.000  
No matches to TargetScan

-----------------

gtaagaagtt

gtaagaagtt  
Depth:4 (HOXB_OPOSSUM)  
Ei-value:Undefined, Pi-value:Undefined  
Er-value:0.000, Pr-value:0.000  
No matches to TargetScan

-------

gaaggga

gaaggga  
Depth:4 (HOXB_OPOSSUM)  
Ei-value:Undefined, Pi-value:Undefined  
Er-value:0.000, Pr-value:0.000  
MATCHES To TargetScan▶ miR-204-5p/211-5p:UCCCUUU

-------------||----- 598  
 ---------------------------------------------------

ggcctc

ggcctc  
Depth:4 (HOXB_OPOSSUM)  
Ei-value:Undefined, Pi-value:Undefined  
Er-value:0.010, Pr-value:0.000  
No matches to TargetScan

--------------------------------------------------------------- 718  
 ----------------------------------------------------------------------------||------------------------------------------ 836  
 -------------------------------------------------------------------------------

tccaggc

tccaggc  
Depth:4 (HOXB_OPOSSUM)  
Ei-value:Undefined, Pi-value:Undefined  
Er-value:0.000, Pr-value:0.000  
No matches to TargetScan

---------------------------------- 956  
 -

gccacc

gccacc  
Depth:5 (HOXB_XENOPUS)  
Ei-value:Undefined, Pi-value:Undefined  
Er-value:0.000, Pr-value:0.000  
No matches to TargetScan

----------------------------------------------------------------------------------------------------------------- 1076  
 ------------------------------------------------------------------------------------------------------------------------ 1196  
 --------------------------------------------------------------------------

taaact

taaact  
Depth:5 (HOXB_XENOPUS)  
Ei-value:Undefined, Pi-value:Undefined  
Er-value:0.000, Pr-value:0.000  
No matches to TargetScan

---------------------------------------- 1316  
 -----------------------------------------------------------------                                                        1381
```

---

## >HOXB\_XENOPUS (647 bases)

```
 -----------------------------------------------------------

gtcata

gtcata  
Depth:5 (HOXB_XENOPUS)  
Ei-value:Undefined, Pi-value:Undefined  
Er-value:0.000, Pr-value:0.000  
No matches to TargetScan

------------------------------------------------------- 120  
 ---------------||------------------------------------------------------------------------------------------------------- 238  
 ------------------------------------------------------------------------------------------------------------------------ 358  
 ---------

gcgacttt

gcgacttt  
Depth:5 (HOXB_XENOPUS)  
Ei-value:Undefined, Pi-value:Undefined  
Er-value:0.000, Pr-value:0.000  
No matches to TargetScan


gccacc

gccacc  
Depth:5 (HOXB_XENOPUS)  
Ei-value:Undefined, Pi-value:Undefined  
Er-value:0.000, Pr-value:0.000  
No matches to TargetScan

------------------------------------------------------------------------------------------------- 478  
 ----------------------------------------------------

taaact

taaact  
Depth:5 (HOXB_XENOPUS)  
Ei-value:Undefined, Pi-value:Undefined  
Er-value:0.000, Pr-value:0.000  
No matches to TargetScan

-------------------------------------------------------------- 598  
 -------------------------------------------------                                                                        647
```

---

## >HOXB\_COELACANTH\_HOXB (974 bases)

```
 NO CONSERVED NODES FOUND  
------------------------------------------------------------------------------------------------------------------------ 120  
 ------------------------------------------------------------------------------------------------------------------------ 240  
 --------------------------------------------------------------------------------------------------------||-------------- 358  
 -----------------------------------------------------------------------------||----------------------------------------- 476  
 ------------------------------------------------------------------------------------------------------------------------ 596  
 ------------------------------------------------------------------------------------------------------------------------ 716  
 ------------------------------------------------------------------------------------------------------------------------ 836  
 ------------------------------------------------------------------------------------------------------------------------ 956  
 ------------------                                                                                                       974
```

---

## >HOXB\_GAR (2133 bases)

```
 NO CONSERVED NODES FOUND  
------------------------------------------------------------------------------------------------------------------------ 120  
 --------------------------------||-------------------------------------------------------------------------------------- 238  
 ------------------------------------------------------------------------------------------------------------------------ 358  
 ------------------------------------------------------------------------------------------------------------------------ 478  
 ------------------------------------------------------------------------------------------------------------------------ 598  
 ------------------------------------------------------------------------------------------------------------------------ 718  
 ------------------------------------------------------------------------------------------------------------------------ 838  
 ------------------------------------------------------------------------------------------------------------------------ 958  
 ------------------------------------------------------------------------------------------------------------------------ 1078  
 ------------------------------------------------------------------------------------------------------------------------ 1198  
 ------------------------------------------------------------------------------------------------------------------------ 1318  
 ------------------------------------------------------------------------------------------------------------------------ 1438  
 ------------------------------------------------------------------------------------------------------------------------ 1558  
 ------------------------------------------------------------------------------------------------------------------------ 1678  
 ------------------------------------------------------------------------------------------------------------------------ 1798  
 ------------------------------------------------------------------------------------------------------------------------ 1918  
 ------------------------------------------------------------------------------------------------------------------------ 2038  
 -----------------------------------------------------------------------------------------------                          2133
```

---

## >HOXB\_SHARK (912 bases)

```
 NO CONSERVED NODES FOUND  
------------------------------------------------------------------------------------------------------------------------ 120  
 ----------------------------------------------------||------------------------------------------------------------------ 238  
 ------------------------------------------------------------------------------------------------------------------------ 358  
 ------------------------------------------------------------------------------------------------------------------------ 478  
 ------------------------------------------------------------------------------------------------------------------------ 598  
 ------------------------------------------------------------------------------------------------------------------------ 718  
 ------------------------------------------------------------------------------------------------------------------------ 838  
 --------------------------------------------------------------------------                                               912
```

---
