# Supplementary material for: Highly conserved and cis-acting lncRNAs produced from paralogous regions in the center of HOXA and HOXB clusters in the endoderm lineage
Source: PLoS Genet. 2021 Jul 19;17(7):e1009681. doi: 10.1371/journal.pgen.1009681 (PMC8330917; doi:10.1371/journal.pgen.1009681)
Supplement: S1 Dataset — (ZIP) [file pgen.1009681.s015.zip › HOXB-AS3_var1/Html_Files/kmers_in_blocks_level.html]

 MOTIFS IN BLOCKS

# MOTIFS IN BLOCK DIAGRAMS

## Motifs conserved to (and beyond) HOXB\_XENOPUS (depth:5)

  

NAVIGATE ▼

▶HOXB-AS3 (depth:1)▶HOXB\_DOG\_ISOFORM1 (depth:2)▶HOXB5OS (depth:3)▶HOXB\_OPOSSUM (depth:4)▶HOXB\_XENOPUS (depth:5)▶HOXB\_COELACANTH\_HOXB (depth:6)▶HOXB\_GAR (depth:7)▶HOXB\_SHARK (depth:8)

  
  
  

## >HOXB-AS3 (573 bases)

```
gtcata

gtcata  
Depth:5 (HOXB_XENOPUS)  
Ei-value:Undefined, Pi-value:Undefined  
Er-value:0.000, Pr-value:0.000  
No matches to eCLIP DataNo matches to TargetScan


gcgacttt

gcgacttt  
Depth:5 (HOXB_XENOPUS)  
Ei-value:Undefined, Pi-value:Undefined  
Er-value:0.000, Pr-value:0.000  
No matches to eCLIP DataNo matches to TargetScan

---------------------------------------------------------------------------------------------------------- 120  
 --------------------------------------------------||-------------------------------------------------------------------- 238  


gccacc

gccacc  
Depth:5 (HOXB_XENOPUS)  
Ei-value:Undefined, Pi-value:Undefined  
Er-value:0.000, Pr-value:0.000  
No matches to eCLIP DataNo matches to TargetScan

----------------------------------------------||------------------------------------------------------------------ 356  
 ------------------------------------------------------

taaact

taaact  
Depth:5 (HOXB_XENOPUS)  
Ei-value:Undefined, Pi-value:Undefined  
Er-value:0.000, Pr-value:0.000  
No matches to eCLIP DataNo matches to TargetScan

------------------------------------------------------------ 476  
 -------------------------------------------------------------------------------------------------                        573
```

---

## >HOXB\_DOG\_ISOFORM1 (2741 bases)

```
 --------------------------------------------------------------------------------

gtcata

gtcata  
Depth:5 (HOXB_XENOPUS)  
Ei-value:Undefined, Pi-value:Undefined  
Er-value:0.000, Pr-value:0.000  
No matches to TargetScan


gcgacttt

gcgacttt  
Depth:5 (HOXB_XENOPUS)  
Ei-value:Undefined, Pi-value:Undefined  
Er-value:0.000, Pr-value:0.000  
No matches to TargetScan

-------------------------- 120  
 ------------------------------------------------------------------------------------------------------------------------ 240  
 ----------||-----------------------------------------------------------------------

gccacc

gccacc  
Depth:5 (HOXB_XENOPUS)  
Ei-value:Undefined, Pi-value:Undefined  
Er-value:0.000, Pr-value:0.000  
No matches to TargetScan

------------------------------- 358  
 ------------------------------------------------------------------------------------------------------------------------ 478  
 ------------------------------------------------------------------------------------------------------------------------ 598  
 ------------------------------------------------------------------------------------------------------------------------ 718  
 ------------------------------------------------------------------------------------------------------------------------ 838  
 ------------------------------------------------------------------------------------------------------------------------ 958  
 ------------------------------------------------------------------------------------------------------------------------ 1078  
 ------------------------------------------------------------------------------------------------------------------------ 1198  
 ------------------------------------------------------------------------------------------------------------------------ 1318  
 ------------------------------------------------------------------------------------------------------------------------ 1438  
 ------------------------------------------------------------------------------------------------------------------------ 1558  
 ------------------------------------------------------------------------------------------------------------------------ 1678  
 ------------------------------------------------------------------------------------------------------------------------ 1798  
 ------------------------------------------------------------------------------------------------------------------------ 1918  
 ------------------------------------------------------------------------------------------------------------------------ 2038  
 ------------------------------------------------------------------------------------------------------------------------ 2158  
 ------------------------------------------------------------------------------------------------------------------------ 2278  
 ------------------------------------------------------------------------------------------------------------------------ 2398  
 ------------------------------------------------------------------------------------------------------------------------ 2518  
 ----------------------------------------------------------------------------------------------------------------------

ta

taaact  
Depth:5 (HOXB_XENOPUS)  
Ei-value:Undefined, Pi-value:Undefined  
Er-value:0.000, Pr-value:0.000  
No matches to TargetScan

 2638  


aact

taaact  
Depth:5 (HOXB_XENOPUS)  
Ei-value:Undefined, Pi-value:Undefined  
Er-value:0.000, Pr-value:0.000  
No matches to TargetScan

---------------------------------------------------------------------------------------------------                  2741
```

---

## >HOXB5OS (596 bases)

```
 --

gtcata

gtcata  
Depth:5 (HOXB_XENOPUS)  
Ei-value:Undefined, Pi-value:Undefined  
Er-value:0.000, Pr-value:0.000  
No matches to TargetScan


gcgacttt

gcgacttt  
Depth:5 (HOXB_XENOPUS)  
Ei-value:Undefined, Pi-value:Undefined  
Er-value:0.000, Pr-value:0.000  
No matches to TargetScan

-------------------------------------------------------------------------------------------------------- 120  
 ------------------------------------------------------||---------------------------------------------------------------- 238  
 -----

gccacc

gccacc  
Depth:5 (HOXB_XENOPUS)  
Ei-value:Undefined, Pi-value:Undefined  
Er-value:0.000, Pr-value:0.000  
No matches to TargetScan

---------------------------------------------------------------------------------------------------------||-- 356  
 --------------------------------------------------------------------------------------------------

taaact

taaact  
Depth:5 (HOXB_XENOPUS)  
Ei-value:Undefined, Pi-value:Undefined  
Er-value:0.000, Pr-value:0.000  
No matches to TargetScan

---------------- 476  
 ------------------------------------------------------------------------------------------------------------------------ 596  
                                                                                                                          596
```

---

## >HOXB\_OPOSSUM (1381 bases)

```
 ------------------------------------------------------------------------------------------------------------------------ 120  
 ------------------------------------------------------------------------------------------------------------------------ 240  
 ------------------------------------------------------------------------------------------------------------------------ 360  
 ---------------------------------------------------------

gtcata

gtcata  
Depth:5 (HOXB_XENOPUS)  
Ei-value:Undefined, Pi-value:Undefined  
Er-value:0.000, Pr-value:0.000  
No matches to TargetScan


gcgacttt

gcgacttt  
Depth:5 (HOXB_XENOPUS)  
Ei-value:Undefined, Pi-value:Undefined  
Er-value:0.000, Pr-value:0.000  
No matches to TargetScan

------------------------------------------------- 480  
 -----------------------------------------------------------------------------------------------------------------||----- 598  
 ------------------------------------------------------------------------------------------------------------------------ 718  
 ----------------------------------------------------------------------------||------------------------------------------ 836  
 ------------------------------------------------------------------------------------------------------------------------ 956  
 -

gccacc

gccacc  
Depth:5 (HOXB_XENOPUS)  
Ei-value:Undefined, Pi-value:Undefined  
Er-value:0.000, Pr-value:0.000  
No matches to TargetScan

----------------------------------------------------------------------------------------------------------------- 1076  
 ------------------------------------------------------------------------------------------------------------------------ 1196  
 --------------------------------------------------------------------------

taaact

taaact  
Depth:5 (HOXB_XENOPUS)  
Ei-value:Undefined, Pi-value:Undefined  
Er-value:0.000, Pr-value:0.000  
No matches to TargetScan

---------------------------------------- 1316  
 -----------------------------------------------------------------                                                        1381
```

---

## >HOXB\_XENOPUS (647 bases)

```
 -----------------------------------------------------------

gtcata

gtcata  
Depth:5 (HOXB_XENOPUS)  
Ei-value:Undefined, Pi-value:Undefined  
Er-value:0.000, Pr-value:0.000  
No matches to TargetScan

------------------------------------------------------- 120  
 ---------------||------------------------------------------------------------------------------------------------------- 238  
 ------------------------------------------------------------------------------------------------------------------------ 358  
 ---------

gcgacttt

gcgacttt  
Depth:5 (HOXB_XENOPUS)  
Ei-value:Undefined, Pi-value:Undefined  
Er-value:0.000, Pr-value:0.000  
No matches to TargetScan


gccacc

gccacc  
Depth:5 (HOXB_XENOPUS)  
Ei-value:Undefined, Pi-value:Undefined  
Er-value:0.000, Pr-value:0.000  
No matches to TargetScan

------------------------------------------------------------------------------------------------- 478  
 ----------------------------------------------------

taaact

taaact  
Depth:5 (HOXB_XENOPUS)  
Ei-value:Undefined, Pi-value:Undefined  
Er-value:0.000, Pr-value:0.000  
No matches to TargetScan

-------------------------------------------------------------- 598  
 -------------------------------------------------                                                                        647
```

---

## >HOXB\_COELACANTH\_HOXB (974 bases)

```
 NO CONSERVED NODES FOUND  
------------------------------------------------------------------------------------------------------------------------ 120  
 ------------------------------------------------------------------------------------------------------------------------ 240  
 --------------------------------------------------------------------------------------------------------||-------------- 358  
 -----------------------------------------------------------------------------||----------------------------------------- 476  
 ------------------------------------------------------------------------------------------------------------------------ 596  
 ------------------------------------------------------------------------------------------------------------------------ 716  
 ------------------------------------------------------------------------------------------------------------------------ 836  
 ------------------------------------------------------------------------------------------------------------------------ 956  
 ------------------                                                                                                       974
```

---

## >HOXB\_GAR (2133 bases)

```
 NO CONSERVED NODES FOUND  
------------------------------------------------------------------------------------------------------------------------ 120  
 --------------------------------||-------------------------------------------------------------------------------------- 238  
 ------------------------------------------------------------------------------------------------------------------------ 358  
 ------------------------------------------------------------------------------------------------------------------------ 478  
 ------------------------------------------------------------------------------------------------------------------------ 598  
 ------------------------------------------------------------------------------------------------------------------------ 718  
 ------------------------------------------------------------------------------------------------------------------------ 838  
 ------------------------------------------------------------------------------------------------------------------------ 958  
 ------------------------------------------------------------------------------------------------------------------------ 1078  
 ------------------------------------------------------------------------------------------------------------------------ 1198  
 ------------------------------------------------------------------------------------------------------------------------ 1318  
 ------------------------------------------------------------------------------------------------------------------------ 1438  
 ------------------------------------------------------------------------------------------------------------------------ 1558  
 ------------------------------------------------------------------------------------------------------------------------ 1678  
 ------------------------------------------------------------------------------------------------------------------------ 1798  
 ------------------------------------------------------------------------------------------------------------------------ 1918  
 ------------------------------------------------------------------------------------------------------------------------ 2038  
 -----------------------------------------------------------------------------------------------                          2133
```

---

## >HOXB\_SHARK (912 bases)

```
 NO CONSERVED NODES FOUND  
------------------------------------------------------------------------------------------------------------------------ 120  
 ----------------------------------------------------||------------------------------------------------------------------ 238  
 ------------------------------------------------------------------------------------------------------------------------ 358  
 ------------------------------------------------------------------------------------------------------------------------ 478  
 ------------------------------------------------------------------------------------------------------------------------ 598  
 ------------------------------------------------------------------------------------------------------------------------ 718  
 ------------------------------------------------------------------------------------------------------------------------ 838  
 --------------------------------------------------------------------------                                               912
```

---
