# Supplementary material for: Highly conserved and cis-acting lncRNAs produced from paralogous regions in the center of HOXA and HOXB clusters in the endoderm lineage
Source: PLoS Genet. 2021 Jul 19;17(7):e1009681. doi: 10.1371/journal.pgen.1009681 (PMC8330917; doi:10.1371/journal.pgen.1009681)
Supplement: S1 Dataset — (ZIP) [file pgen.1009681.s015.zip › HOXB-AS3_var1/Html_Files/miRNA_Matches.html]

 miRNA Matches

# Matches to miRNA Families Retrieved from TargetScan

  

\*\*SEED defined as positions 2-7 of mature miRNA sequence  
\*\*MOTIF MATCHES that correspond to the reverse complement of miRNA SEEDS are displayed from 5`-3` relative to lincRNA sequence

  

| Seed Matches to the miRNA miR-485-5p | | | | | |
| --- | --- | --- | --- | --- | --- |
| Seed | Conservation | Species | Matches | | |
|  |  |  | Sequence | Motif | Type |
| GAGGCUG | Conserved | Human (Homo sapiens)  Rhesus (Macaca mulatta)  Rat (Rattus norvegicus) | HOXB-AS3 | AGTAGAGCCTC | 6mer |
|  |  |  | HOXB-AS3 | AGCCTC | 6mer |
|  |  |  | HOXB\_DOG\_ISOFORM1 | AGTAGAGCCTC | 6mer |
|  |  |  | HOXB\_DOG\_ISOFORM1 | AGCCTC | 6mer |
|  |  |  | HOXB5OS | AGCCTC | 7mer-m8 |

  
  
  
  

| Seed Matches to the miRNA miR-760 | | | | | |
| --- | --- | --- | --- | --- | --- |
| Seed | Conservation | Species | Matches | | |
|  |  |  | Sequence | Motif | Type |
| GGCUCUG | Conserved | Human (Homo sapiens)  Rhesus (Macaca mulatta)  Chimp (Pan troglodytes) | HOXB-AS3 | AGTAGAGCCTC | 6mer |
|  |  |  | HOXB\_DOG\_ISOFORM1 | AGTAGAGCCTC | 6mer |

  
  
  
  

| Seed Matches to the miRNA miR-1224-5p | | | | | |
| --- | --- | --- | --- | --- | --- |
| Seed | Conservation | Species | Matches | | |
|  |  |  | Sequence | Motif | Type |
| UGAGGAC | Conserved | Human (Homo sapiens)  Rhesus (Macaca mulatta)  Chimp (Pan troglodytes)  Mouse (Mus musculus) | HOXB-AS3 | CTCCTCACCAGCTCCCC | 6mer |
|  |  |  | HOXB\_DOG\_ISOFORM1 | CTCCTCACCAGCTCCCC | 6mer |

  
  
  
  

| Seed Matches to the miRNA miR-138-5p | | | | | |
| --- | --- | --- | --- | --- | --- |
| Seed | Conservation | Species | Matches | | |
|  |  |  | Sequence | Motif | Type |
| GCUGGUG | Broadly Conserved | Human (Homo sapiens)  Chicken (Gallus gallus)  Rhesus (Macaca mulatta)  Mouse (Mus musculus)  Rat (Rattus norvegicus) | HOXB-AS3 | CTCCTCACCAGCTCCCC | 7mer-m8 |
|  |  |  | HOXB\_DOG\_ISOFORM1 | CTCCTCACCAGCTCCCC | 7mer-m8 |

  
  
  
  

| Seed Matches to the miRNA miR-188-5p | | | | | |
| --- | --- | --- | --- | --- | --- |
| Seed | Conservation | Species | Matches | | |
|  |  |  | Sequence | Motif | Type |
| AUCCCUU | Conserved | Human (Homo sapiens)  Rhesus (Macaca mulatta)  Mouse (Mus musculus)  Rat (Rattus norvegicus) | HOXB-AS3 | GTAAGAAGTTGGGCCAAGCTGGAAGGGATTGACCGGCCG | 7mer-m8 |
|  |  |  | HOXB-AS3 | AGCTGGAAGGGATTGACCG | 7mer-m8 |
|  |  |  | HOXB\_DOG\_ISOFORM1 | GTAAGAAGTTGGGCCAAGCTGGAAGGGATTGACCGGCCG | 7mer-m8 |
|  |  |  | HOXB\_DOG\_ISOFORM1 | AGCTGGAAGGGATTGACCG | 7mer-m8 |
|  |  |  | HOXB5OS | AGCTGGAAGGGATTGACCG | 7mer-m8 |

  
  
  
  

| Seed Matches to the miRNA miR-204-5p/211-5p | | | | | |
| --- | --- | --- | --- | --- | --- |
| Seed | Conservation | Species | Matches | | |
|  |  |  | Sequence | Motif | Type |
| UCCCUUU | Broadly Conserved | Human (Homo sapiens)  Mouse (Mus musculus)  Rat (Rattus norvegicus) | HOXB-AS3 | GTAAGAAGTTGGGCCAAGCTGGAAGGGATTGACCGGCCG | 6mer |
|  |  |  | HOXB-AS3 | AGCTGGAAGGGATTGACCG | 6mer |
|  |  |  | HOXB-AS3 | GAAGGGA | 6mer |
|  |  |  | HOXB\_DOG\_ISOFORM1 | GTAAGAAGTTGGGCCAAGCTGGAAGGGATTGACCGGCCG | 6mer |
|  |  |  | HOXB\_DOG\_ISOFORM1 | AGCTGGAAGGGATTGACCG | 6mer |
|  |  |  | HOXB\_DOG\_ISOFORM1 | GAAGGGA | 6mer |
|  |  |  | HOXB5OS | AGCTGGAAGGGATTGACCG | 6mer |
|  |  |  | HOXB5OS | GAAGGGA | 6mer |
|  |  |  | HOXB\_OPOSSUM | GAAGGGA | 6mer |

  
  
  
  

| Seed Matches to the miRNA miR-328-3p | | | | | |
| --- | --- | --- | --- | --- | --- |
| Seed | Conservation | Species | Matches | | |
|  |  |  | Sequence | Motif | Type |
| UGGCCCU | Conserved | Human (Homo sapiens)  Mouse (Mus musculus)  Rat (Rattus norvegicus) | HOXB-AS3 | GTAAGAAGTTGGGCCAAGCTGGAAGGGATTGACCGGCCG | 7mer-A1 |
|  |  |  | HOXB\_DOG\_ISOFORM1 | GTAAGAAGTTGGGCCAAGCTGGAAGGGATTGACCGGCCG | 7mer-A1 |

  
  
  
  

| Seed Matches to the miRNA miR-216a-5p | | | | | |
| --- | --- | --- | --- | --- | --- |
| Seed | Conservation | Species | Matches | | |
|  |  |  | Sequence | Motif | Type |
| AAUCUCA | Broadly Conserved | Human (Homo sapiens)  Mouse (Mus musculus)  Rat (Rattus norvegicus) | HOXB-AS3 | GCGGAGATTCCAGGCCCT | 6mer |
|  |  |  | HOXB-AS3 | GCGGAGATTCCAGGCCC | 6mer |
|  |  |  | HOXB\_DOG\_ISOFORM1 | GCGGAGATTCCAGGCCCT | 6mer |
|  |  |  | HOXB\_DOG\_ISOFORM1 | GCGGAGATTCCAGGCCC | 6mer |
|  |  |  | HOXB5OS | GCGGAGATTCCAGGCCC | 6mer |

  
  
  
  

| Seed Matches to the miRNA miR-216b-5p | | | | | |
| --- | --- | --- | --- | --- | --- |
| Seed | Conservation | Species | Matches | | |
|  |  |  | Sequence | Motif | Type |
| AAUCUCU | Broadly Conserved | Human (Homo sapiens)  Mouse (Mus musculus)  Rat (Rattus norvegicus) | HOXB-AS3 | GCGGAGATTCCAGGCCCT | 6mer |
|  |  |  | HOXB-AS3 | GCGGAGATTCCAGGCCC | 6mer |
|  |  |  | HOXB\_DOG\_ISOFORM1 | GCGGAGATTCCAGGCCCT | 6mer |
|  |  |  | HOXB\_DOG\_ISOFORM1 | GCGGAGATTCCAGGCCC | 6mer |
|  |  |  | HOXB5OS | GCGGAGATTCCAGGCCC | 6mer |

  
  
  
  

| Seed Matches to the miRNA miR-324-5p | | | | | |
| --- | --- | --- | --- | --- | --- |
| Seed | Conservation | Species | Matches | | |
|  |  |  | Sequence | Motif | Type |
| GCAUCCC | Conserved | Human (Homo sapiens)  Rhesus (Macaca mulatta)  Mouse (Mus musculus)  Rat (Rattus norvegicus) | HOXB-AS3 | GAGCGGCCGGGATGCGGCCACACC | 7mer-m8 |
|  |  |  | HOXB\_DOG\_ISOFORM1 | GAGCGGCCGGGATGCGGCCACACC | 7mer-m8 |

  
  
  
  

| Seed Matches to the miRNA miR-491-5p | | | | | |
| --- | --- | --- | --- | --- | --- |
| Seed | Conservation | Species | Matches | | |
|  |  |  | Sequence | Motif | Type |
| GUGGGGA | Conserved | Human (Homo sapiens)  Rhesus (Macaca mulatta)  Mouse (Mus musculus) | HOXB-AS3 | TCCCCAC | 7mer-m8 |
|  |  |  | HOXB\_DOG\_ISOFORM1 | TCCCCAC | 7mer-m8 |

  
  
  
  
