# Supplementary material for: Highly conserved and cis-acting lncRNAs produced from paralogous regions in the center of HOXA and HOXB clusters in the endoderm lineage
Source: PLoS Genet. 2021 Jul 19;17(7):e1009681. doi: 10.1371/journal.pgen.1009681 (PMC8330917; doi:10.1371/journal.pgen.1009681)
Supplement: S1 Dataset — (ZIP) [file pgen.1009681.s015.zip › HOXB-AS3_var1/Html_Files/kmers_in_blocks_layer_conservation.html]

 BLOCKS\_CONSERVATION

# MOTIF CONSERVATION IN BLOCK DIAGRAMS

## Motifs mapped to anchor sequence

  

NAVIGATE ▼

▶HOXB\_DOG\_ISOFORM1▶HOXB5OS▶HOXB\_OPOSSUM▶HOXB\_XENOPUS▶HOXB\_COELACANTH\_HOXB▶HOXB\_GAR▶HOXB\_SHARK

  
  
  

## >HOXB-AS3 TO HOXB\_DOG\_ISOFORM1 (573 bases)

```
gtcata

gtcata  
Depth:5 (HOXB_XENOPUS)  
Ei-value:Undefined, Pi-value:Undefined  
Er-value:0.000, Pr-value:0.000  
No matches to eCLIP DataNo matches to TargetScan


gcgacttt

gcgacttt  
Depth:5 (HOXB_XENOPUS)  
Ei-value:Undefined, Pi-value:Undefined  
Er-value:0.000, Pr-value:0.000  
No matches to eCLIP DataNo matches to TargetScan


tggg

gtcatagcgacttttggg  
Depth:4 (HOXB_OPOSSUM)  
Ei-value:Undefined, Pi-value:Undefined  
Er-value:0.000, Pr-value:0.000  
No matches to eCLIP DataNo matches to TargetScan


a

gtcatagcgacttttgggatagtttgctat  
Depth:2 (HOXB_DOG_ISOFORM1)  
Ei-value:Undefined, Pi-value:Undefined  
Er-value:0.000, Pr-value:0.000  
No matches to eCLIP DataNo matches to TargetScan


tagtttgct

tagtttgct  
Depth:4 (HOXB_OPOSSUM)  
Ei-value:Undefined, Pi-value:Undefined  
Er-value:0.000, Pr-value:0.000  
No matches to eCLIP DataNo matches to TargetScan


at

gtcatagcgacttttgggatagtttgctat  
Depth:2 (HOXB_DOG_ISOFORM1)  
Ei-value:Undefined, Pi-value:Undefined  
Er-value:0.000, Pr-value:0.000  
No matches to eCLIP DataNo matches to TargetScan

-

ga

gacaaaggg  
Depth:2 (HOXB_DOG_ISOFORM1)  
Ei-value:Undefined, Pi-value:Undefined  
Er-value:0.000, Pr-value:0.000  
No matches to eCLIP DataNo matches to TargetScan


caaaggg

caaaggg  
Depth:4 (HOXB_OPOSSUM)  
Ei-value:Undefined, Pi-value:Undefined  
Er-value:0.000, Pr-value:0.000  
No matches to eCLIP DataNo matches to TargetScan

-

gacaaagtca

gacaaagtca  
Depth:3 (HOXB5OS)  
Ei-value:Undefined, Pi-value:Undefined  
Er-value:0.000, Pr-value:0.000  
No matches to eCLIP DataNo matches to TargetScan


agggg

gacaaagtcaagggg  
Depth:2 (HOXB_DOG_ISOFORM1)  
Ei-value:Undefined, Pi-value:Undefined  
Er-value:0.000, Pr-value:0.000  
No matches to eCLIP DataNo matches to TargetScan

--------

aaggagg

aaggagg  
Depth:3 (HOXB5OS)  
Ei-value:Undefined, Pi-value:Undefined  
Er-value:0.000, Pr-value:0.000  
No matches to eCLIP DataNo matches to TargetScan


gcc

aaggagggcc  
Depth:2 (HOXB_DOG_ISOFORM1)  
Ei-value:Undefined, Pi-value:Undefined  
Er-value:0.000, Pr-value:0.000  
No matches to eCLIP DataNo matches to TargetScan

-

agtag

agtagagcctc  
Depth:2 (HOXB_DOG_ISOFORM1)  
Ei-value:Undefined, Pi-value:Undefined  
Er-value:0.000, Pr-value:0.000  
No matches to eCLIP DataMATCHES To TargetScan▶ miR-485-5p:GAGGCUG▶ miR-760:GGCUCUG


agcctc

agcctc  
Depth:3 (HOXB5OS)  
Ei-value:Undefined, Pi-value:Undefined  
Er-value:0.000, Pr-value:0.000  
No matches to eCLIP DataMATCHES To TargetScan▶ miR-485-5p:GAGGCUG

----------------

ct

ctcctcaccagctcccc  
Depth:2 (HOXB_DOG_ISOFORM1)  
Ei-value:Undefined, Pi-value:Undefined  
Er-value:0.000, Pr-value:0.000  
No matches to eCLIP DataMATCHES To TargetScan▶ miR-1224-5p:UGAGGAC▶ miR-138-5p:GCUGGUG


cctcacca

cctcacca  
Depth:3 (HOXB5OS)  
Ei-value:Undefined, Pi-value:Undefined  
Er-value:0.000, Pr-value:0.000  
No matches to eCLIP DataNo matches to TargetScan


g

gctcccc  
Depth:3 (HOXB5OS)  
Ei-value:Undefined, Pi-value:Undefined  
Er-value:0.000, Pr-value:0.000  
No matches to eCLIP DataNo matches to TargetScan


ctcccc

ctcccc  
Depth:4 (HOXB_OPOSSUM)  
Ei-value:Undefined, Pi-value:Undefined  
Er-value:0.010, Pr-value:0.000  
No matches to eCLIP DataNo matches to TargetScan

- 120  
 -----

ccaagtcc

ccaagtcc  
Depth:2 (HOXB_DOG_ISOFORM1)  
Ei-value:Undefined, Pi-value:Undefined  
Er-value:0.000, Pr-value:0.000  
No matches to eCLIP DataNo matches to TargetScan

-

gtaagaagtt

gtaagaagtt  
Depth:4 (HOXB_OPOSSUM)  
Ei-value:Undefined, Pi-value:Undefined  
Er-value:0.000, Pr-value:0.000  
No matches to eCLIP DataNo matches to TargetScan


gggcc

gtaagaagttgggcc  
Depth:3 (HOXB5OS)  
Ei-value:Undefined, Pi-value:Undefined  
Er-value:0.000, Pr-value:0.000  
No matches to eCLIP DataNo matches to TargetScan


a

gtaagaagttgggccaagctggaagggattgaccggccg  
Depth:2 (HOXB_DOG_ISOFORM1)  
Ei-value:Undefined, Pi-value:Undefined  
Er-value:0.000, Pr-value:0.000  
No matches to eCLIP DataMATCHES To TargetScan▶ miR-188-5p:AUCCCUU▶ miR-204-5p/211-5p:UCCCUUU▶ miR-328-3p:UGGCCCU


agctg

agctggaagggattgaccg  
Depth:3 (HOXB5OS)  
Ei-value:Undefined, Pi-value:Undefined  
Er-value:0.000, Pr-value:0.000  
No matches to eCLIP DataMATCHES To TargetScan▶ miR-188-5p:AUCCCUU▶ miR-204-5p/211-5p:UCCCUUU


gaaggga

gaaggga  
Depth:4 (HOXB_OPOSSUM)  
Ei-value:Undefined, Pi-value:Undefined  
Er-value:0.000, Pr-value:0.000  
No matches to eCLIP DataMATCHES To TargetScan▶ miR-204-5p/211-5p:UCCCUUU


ttgaccg

agctggaagggattgaccg  
Depth:3 (HOXB5OS)  
Ei-value:Undefined, Pi-value:Undefined  
Er-value:0.000, Pr-value:0.000  
No matches to eCLIP DataMATCHES To TargetScan▶ miR-188-5p:AUCCCUU▶ miR-204-5p/211-5p:UCCCUUU


g||ccg

gtaagaagttgggccaagctggaagggattgaccggccg  
Depth:2 (HOXB_DOG_ISOFORM1)  
Ei-value:Undefined, Pi-value:Undefined  
Er-value:0.000, Pr-value:0.000  
No matches to eCLIP DataMATCHES To TargetScan▶ miR-188-5p:AUCCCUU▶ miR-204-5p/211-5p:UCCCUUU▶ miR-328-3p:UGGCCCU

---------

cctcgcc

cctcgcc  
Depth:2 (HOXB_DOG_ISOFORM1)  
Ei-value:Undefined, Pi-value:Undefined  
Er-value:0.000, Pr-value:0.000  
No matches to eCLIP DataNo matches to TargetScan


ggcctc

ggcctc  
Depth:4 (HOXB_OPOSSUM)  
Ei-value:Undefined, Pi-value:Undefined  
Er-value:0.010, Pr-value:0.000  
No matches to eCLIP DataNo matches to TargetScan

-

gcggagat

gcggagattccaggccc  
Depth:3 (HOXB5OS)  
Ei-value:Undefined, Pi-value:Undefined  
Er-value:0.000, Pr-value:0.000  
No matches to eCLIP DataMATCHES To TargetScan▶ miR-216a-5p:AAUCUCA▶ miR-216b-5p:AAUCUCU


tccaggc

tccaggc  
Depth:4 (HOXB_OPOSSUM)  
Ei-value:Undefined, Pi-value:Undefined  
Er-value:0.000, Pr-value:0.000  
No matches to eCLIP DataNo matches to TargetScan


cc

gcggagattccaggccc  
Depth:3 (HOXB5OS)  
Ei-value:Undefined, Pi-value:Undefined  
Er-value:0.000, Pr-value:0.000  
No matches to eCLIP DataMATCHES To TargetScan▶ miR-216a-5p:AAUCUCA▶ miR-216b-5p:AAUCUCU


t

gcggagattccaggccct  
Depth:2 (HOXB_DOG_ISOFORM1)  
Ei-value:Undefined, Pi-value:Undefined  
Er-value:0.000, Pr-value:0.000  
No matches to eCLIP DataMATCHES To TargetScan▶ miR-216a-5p:AAUCUCA▶ miR-216b-5p:AAUCUCU

----------

ggacgtccct

ggacgtccct  
Depth:2 (HOXB_DOG_ISOFORM1)  
Ei-value:Undefined, Pi-value:Undefined  
Er-value:0.000, Pr-value:0.000  
No matches to eCLIP DataNo matches to TargetScan

-

agc

agcgccaccgcc  
Depth:3 (HOXB5OS)  
Ei-value:Undefined, Pi-value:Undefined  
Er-value:0.000, Pr-value:0.000  
No matches to eCLIP DataNo matches to TargetScan

 238  


agcgccaccgcc  
Depth:3 (HOXB5OS)  
Ei-value:Undefined, Pi-value:Undefined  
Er-value:0.000, Pr-value:0.000  
No matches to eCLIP DataNo matches to TargetScan


gccacc

gccacc  
Depth:5 (HOXB_XENOPUS)  
Ei-value:Undefined, Pi-value:Undefined  
Er-value:0.000, Pr-value:0.000  
No matches to eCLIP DataNo matches to TargetScan


gcc

agcgccaccgcc  
Depth:3 (HOXB5OS)  
Ei-value:Undefined, Pi-value:Undefined  
Er-value:0.000, Pr-value:0.000  
No matches to eCLIP DataNo matches to TargetScan

----------------------------------

ccgcacc

ccgcacc  
Depth:2 (HOXB_DOG_ISOFORM1)  
Ei-value:Undefined, Pi-value:Undefined  
Er-value:0.000, Pr-value:0.000  
No matches to eCLIP DataNo matches to TargetScan

--||------------

caggctgc

caggctgc  
Depth:2 (HOXB_DOG_ISOFORM1)  
Ei-value:Undefined, Pi-value:Undefined  
Er-value:0.000, Pr-value:0.000  
No matches to eCLIP DataNo matches to TargetScan

----

ggcggcgc

ggcggcgc  
Depth:2 (HOXB_DOG_ISOFORM1)  
Ei-value:Undefined, Pi-value:Undefined  
Er-value:0.000, Pr-value:0.000  
No matches to eCLIP DataNo matches to TargetScan

-------------------------------

ccg

ccgggc  
Depth:2 (HOXB_DOG_ISOFORM1)  
Ei-value:Undefined, Pi-value:Undefined  
Er-value:0.090, Pr-value:0.010  
No matches to eCLIP DataNo matches to TargetScan

 356  


ggc

ccgggc  
Depth:2 (HOXB_DOG_ISOFORM1)  
Ei-value:Undefined, Pi-value:Undefined  
Er-value:0.090, Pr-value:0.010  
No matches to eCLIP DataNo matches to TargetScan

--------------------

gagcg

gagcggccgggatgcggccacacc  
Depth:2 (HOXB_DOG_ISOFORM1)  
Ei-value:Undefined, Pi-value:Undefined  
Er-value:0.000, Pr-value:0.000  
No matches to eCLIP DataMATCHES To TargetScan▶ miR-324-5p:GCAUCCC


gccggga

gccggga  
Depth:3 (HOXB5OS)  
Ei-value:Undefined, Pi-value:Undefined  
Er-value:0.000, Pr-value:0.000  
No matches to eCLIP DataNo matches to TargetScan


tgcgg

gagcggccgggatgcggccacacc  
Depth:2 (HOXB_DOG_ISOFORM1)  
Ei-value:Undefined, Pi-value:Undefined  
Er-value:0.000, Pr-value:0.000  
No matches to eCLIP DataMATCHES To TargetScan▶ miR-324-5p:GCAUCCC


ccacac

ccacac  
Depth:3 (HOXB5OS)  
Ei-value:Undefined, Pi-value:Undefined  
Er-value:0.000, Pr-value:0.000  
No matches to eCLIP DataNo matches to TargetScan


c

gagcggccgggatgcggccacacc  
Depth:2 (HOXB_DOG_ISOFORM1)  
Ei-value:Undefined, Pi-value:Undefined  
Er-value:0.000, Pr-value:0.000  
No matches to eCLIP DataMATCHES To TargetScan▶ miR-324-5p:GCAUCCC

-----

gg

ggtaaact  
Depth:3 (HOXB5OS)  
Ei-value:Undefined, Pi-value:Undefined  
Er-value:0.000, Pr-value:0.000  
No matches to eCLIP DataNo matches to TargetScan


taaact

taaact  
Depth:5 (HOXB_XENOPUS)  
Ei-value:Undefined, Pi-value:Undefined  
Er-value:0.000, Pr-value:0.000  
No matches to eCLIP DataNo matches to TargetScan

-------------------------------------

tccccac

tccccac  
Depth:2 (HOXB_DOG_ISOFORM1)  
Ei-value:Undefined, Pi-value:Undefined  
Er-value:0.000, Pr-value:0.000  
No matches to eCLIP DataMATCHES To TargetScan▶ miR-491-5p:GUGGGGA

--------------

tt

ttttatttgg  
Depth:2 (HOXB_DOG_ISOFORM1)  
Ei-value:Undefined, Pi-value:Undefined  
Er-value:0.000, Pr-value:0.000  
No matches to eCLIP DataNo matches to TargetScan

 476  


ttatttgg

ttttatttgg  
Depth:2 (HOXB_DOG_ISOFORM1)  
Ei-value:Undefined, Pi-value:Undefined  
Er-value:0.000, Pr-value:0.000  
No matches to eCLIP DataNo matches to TargetScan

------------

aatttagaa

aatttagaa  
Depth:3 (HOXB5OS)  
Ei-value:Undefined, Pi-value:Undefined  
Er-value:0.000, Pr-value:0.000  
No matches to eCLIP DataNo matches to TargetScan

-

gagataaa

gagataaa  
Depth:2 (HOXB_DOG_ISOFORM1)  
Ei-value:Undefined, Pi-value:Undefined  
Er-value:0.000, Pr-value:0.000  
No matches to eCLIP DataNo matches to TargetScan

-----------------------------------------------------------                        573
```

|  |  |  |  |  |  |  |
| --- | --- | --- | --- | --- | --- | --- |
| | | | | | | | | | | | | | |
| 2 |  |  | 5 |  |  | 8 |
| Depth of motif conservation (number of species) | | | | | | |

  
  

---

## >HOXB-AS3 TO HOXB5OS (573 bases)

```
gtcata

gtcata  
Depth:5 (HOXB_XENOPUS)  
Ei-value:Undefined, Pi-value:Undefined  
Er-value:0.000, Pr-value:0.000  
No matches to eCLIP DataNo matches to TargetScan


gcgacttt

gcgacttt  
Depth:5 (HOXB_XENOPUS)  
Ei-value:Undefined, Pi-value:Undefined  
Er-value:0.000, Pr-value:0.000  
No matches to eCLIP DataNo matches to TargetScan


tggg

gtcatagcgacttttggg  
Depth:4 (HOXB_OPOSSUM)  
Ei-value:Undefined, Pi-value:Undefined  
Er-value:0.000, Pr-value:0.000  
No matches to eCLIP DataNo matches to TargetScan

-

tagtttgct

tagtttgct  
Depth:4 (HOXB_OPOSSUM)  
Ei-value:Undefined, Pi-value:Undefined  
Er-value:0.000, Pr-value:0.000  
No matches to eCLIP DataNo matches to TargetScan

-----

caaaggg

caaaggg  
Depth:4 (HOXB_OPOSSUM)  
Ei-value:Undefined, Pi-value:Undefined  
Er-value:0.000, Pr-value:0.000  
No matches to eCLIP DataNo matches to TargetScan

-

gacaaagtca

gacaaagtca  
Depth:3 (HOXB5OS)  
Ei-value:Undefined, Pi-value:Undefined  
Er-value:0.000, Pr-value:0.000  
No matches to eCLIP DataNo matches to TargetScan

-------------

aaggagg

aaggagg  
Depth:3 (HOXB5OS)  
Ei-value:Undefined, Pi-value:Undefined  
Er-value:0.000, Pr-value:0.000  
No matches to eCLIP DataNo matches to TargetScan

---------

agcctc

agcctc  
Depth:3 (HOXB5OS)  
Ei-value:Undefined, Pi-value:Undefined  
Er-value:0.000, Pr-value:0.000  
No matches to eCLIP DataMATCHES To TargetScan▶ miR-485-5p:GAGGCUG

------------------

cctcacca

cctcacca  
Depth:3 (HOXB5OS)  
Ei-value:Undefined, Pi-value:Undefined  
Er-value:0.000, Pr-value:0.000  
No matches to eCLIP DataNo matches to TargetScan


g

gctcccc  
Depth:3 (HOXB5OS)  
Ei-value:Undefined, Pi-value:Undefined  
Er-value:0.000, Pr-value:0.000  
No matches to eCLIP DataNo matches to TargetScan


ctcccc

ctcccc  
Depth:4 (HOXB_OPOSSUM)  
Ei-value:Undefined, Pi-value:Undefined  
Er-value:0.010, Pr-value:0.000  
No matches to eCLIP DataNo matches to TargetScan

- 120  
 --------------

gtaagaagtt

gtaagaagtt  
Depth:4 (HOXB_OPOSSUM)  
Ei-value:Undefined, Pi-value:Undefined  
Er-value:0.000, Pr-value:0.000  
No matches to eCLIP DataNo matches to TargetScan


gggcc

gtaagaagttgggcc  
Depth:3 (HOXB5OS)  
Ei-value:Undefined, Pi-value:Undefined  
Er-value:0.000, Pr-value:0.000  
No matches to eCLIP DataNo matches to TargetScan

-

agctg

agctggaagggattgaccg  
Depth:3 (HOXB5OS)  
Ei-value:Undefined, Pi-value:Undefined  
Er-value:0.000, Pr-value:0.000  
No matches to eCLIP DataMATCHES To TargetScan▶ miR-188-5p:AUCCCUU▶ miR-204-5p/211-5p:UCCCUUU


gaaggga

gaaggga  
Depth:4 (HOXB_OPOSSUM)  
Ei-value:Undefined, Pi-value:Undefined  
Er-value:0.000, Pr-value:0.000  
No matches to eCLIP DataMATCHES To TargetScan▶ miR-204-5p/211-5p:UCCCUUU


ttgaccg

agctggaagggattgaccg  
Depth:3 (HOXB5OS)  
Ei-value:Undefined, Pi-value:Undefined  
Er-value:0.000, Pr-value:0.000  
No matches to eCLIP DataMATCHES To TargetScan▶ miR-188-5p:AUCCCUU▶ miR-204-5p/211-5p:UCCCUUU

-||-------------------

ggcctc

ggcctc  
Depth:4 (HOXB_OPOSSUM)  
Ei-value:Undefined, Pi-value:Undefined  
Er-value:0.010, Pr-value:0.000  
No matches to eCLIP DataNo matches to TargetScan

-

gcggagat

gcggagattccaggccc  
Depth:3 (HOXB5OS)  
Ei-value:Undefined, Pi-value:Undefined  
Er-value:0.000, Pr-value:0.000  
No matches to eCLIP DataMATCHES To TargetScan▶ miR-216a-5p:AAUCUCA▶ miR-216b-5p:AAUCUCU


tccaggc

tccaggc  
Depth:4 (HOXB_OPOSSUM)  
Ei-value:Undefined, Pi-value:Undefined  
Er-value:0.000, Pr-value:0.000  
No matches to eCLIP DataNo matches to TargetScan


cc

gcggagattccaggccc  
Depth:3 (HOXB5OS)  
Ei-value:Undefined, Pi-value:Undefined  
Er-value:0.000, Pr-value:0.000  
No matches to eCLIP DataMATCHES To TargetScan▶ miR-216a-5p:AAUCUCA▶ miR-216b-5p:AAUCUCU

----------------------

agc

agcgccaccgcc  
Depth:3 (HOXB5OS)  
Ei-value:Undefined, Pi-value:Undefined  
Er-value:0.000, Pr-value:0.000  
No matches to eCLIP DataNo matches to TargetScan

 238  


agcgccaccgcc  
Depth:3 (HOXB5OS)  
Ei-value:Undefined, Pi-value:Undefined  
Er-value:0.000, Pr-value:0.000  
No matches to eCLIP DataNo matches to TargetScan


gccacc

gccacc  
Depth:5 (HOXB_XENOPUS)  
Ei-value:Undefined, Pi-value:Undefined  
Er-value:0.000, Pr-value:0.000  
No matches to eCLIP DataNo matches to TargetScan


gcc

agcgccaccgcc  
Depth:3 (HOXB5OS)  
Ei-value:Undefined, Pi-value:Undefined  
Er-value:0.000, Pr-value:0.000  
No matches to eCLIP DataNo matches to TargetScan

-------------------------------------------||------------------------------------------------------------------ 356  
 ----------------------------

gccggga

gccggga  
Depth:3 (HOXB5OS)  
Ei-value:Undefined, Pi-value:Undefined  
Er-value:0.000, Pr-value:0.000  
No matches to eCLIP DataNo matches to TargetScan

-----

ccacac

ccacac  
Depth:3 (HOXB5OS)  
Ei-value:Undefined, Pi-value:Undefined  
Er-value:0.000, Pr-value:0.000  
No matches to eCLIP DataNo matches to TargetScan

------

gg

ggtaaact  
Depth:3 (HOXB5OS)  
Ei-value:Undefined, Pi-value:Undefined  
Er-value:0.000, Pr-value:0.000  
No matches to eCLIP DataNo matches to TargetScan


taaact

taaact  
Depth:5 (HOXB_XENOPUS)  
Ei-value:Undefined, Pi-value:Undefined  
Er-value:0.000, Pr-value:0.000  
No matches to eCLIP DataNo matches to TargetScan

------------------------------------------------------------ 476  
 --------------------

aatttagaa

aatttagaa  
Depth:3 (HOXB5OS)  
Ei-value:Undefined, Pi-value:Undefined  
Er-value:0.000, Pr-value:0.000  
No matches to eCLIP DataNo matches to TargetScan

--------------------------------------------------------------------                        573
```

|  |  |  |  |  |  |  |
| --- | --- | --- | --- | --- | --- | --- |
| | | | | | | | | | | | | | |
| 2 |  |  | 5 |  |  | 8 |
| Depth of motif conservation (number of species) | | | | | | |

  
  

---

## >HOXB-AS3 TO HOXB\_OPOSSUM (573 bases)

```
gtcata

gtcata  
Depth:5 (HOXB_XENOPUS)  
Ei-value:Undefined, Pi-value:Undefined  
Er-value:0.000, Pr-value:0.000  
No matches to eCLIP DataNo matches to TargetScan


gcgacttt

gcgacttt  
Depth:5 (HOXB_XENOPUS)  
Ei-value:Undefined, Pi-value:Undefined  
Er-value:0.000, Pr-value:0.000  
No matches to eCLIP DataNo matches to TargetScan


tggg

gtcatagcgacttttggg  
Depth:4 (HOXB_OPOSSUM)  
Ei-value:Undefined, Pi-value:Undefined  
Er-value:0.000, Pr-value:0.000  
No matches to eCLIP DataNo matches to TargetScan

-

tagtttgct

tagtttgct  
Depth:4 (HOXB_OPOSSUM)  
Ei-value:Undefined, Pi-value:Undefined  
Er-value:0.000, Pr-value:0.000  
No matches to eCLIP DataNo matches to TargetScan

-----

caaaggg

caaaggg  
Depth:4 (HOXB_OPOSSUM)  
Ei-value:Undefined, Pi-value:Undefined  
Er-value:0.000, Pr-value:0.000  
No matches to eCLIP DataNo matches to TargetScan

-------------------------------------------------------------------------

ctcccc

ctcccc  
Depth:4 (HOXB_OPOSSUM)  
Ei-value:Undefined, Pi-value:Undefined  
Er-value:0.010, Pr-value:0.000  
No matches to eCLIP DataNo matches to TargetScan

- 120  
 --------------

gtaagaagtt

gtaagaagtt  
Depth:4 (HOXB_OPOSSUM)  
Ei-value:Undefined, Pi-value:Undefined  
Er-value:0.000, Pr-value:0.000  
No matches to eCLIP DataNo matches to TargetScan

-----------

gaaggga

gaaggga  
Depth:4 (HOXB_OPOSSUM)  
Ei-value:Undefined, Pi-value:Undefined  
Er-value:0.000, Pr-value:0.000  
No matches to eCLIP DataMATCHES To TargetScan▶ miR-204-5p/211-5p:UCCCUUU

--------||-------------------

ggcctc

ggcctc  
Depth:4 (HOXB_OPOSSUM)  
Ei-value:Undefined, Pi-value:Undefined  
Er-value:0.010, Pr-value:0.000  
No matches to eCLIP DataNo matches to TargetScan

---------

tccaggc

tccaggc  
Depth:4 (HOXB_OPOSSUM)  
Ei-value:Undefined, Pi-value:Undefined  
Er-value:0.000, Pr-value:0.000  
No matches to eCLIP DataNo matches to TargetScan

--------------------------- 238  


gccacc

gccacc  
Depth:5 (HOXB_XENOPUS)  
Ei-value:Undefined, Pi-value:Undefined  
Er-value:0.000, Pr-value:0.000  
No matches to eCLIP DataNo matches to TargetScan

----------------------------------------------||------------------------------------------------------------------ 356  
 ------------------------------------------------------

taaact

taaact  
Depth:5 (HOXB_XENOPUS)  
Ei-value:Undefined, Pi-value:Undefined  
Er-value:0.000, Pr-value:0.000  
No matches to eCLIP DataNo matches to TargetScan

------------------------------------------------------------ 476  
 -------------------------------------------------------------------------------------------------                        573
```

|  |  |  |  |  |  |  |
| --- | --- | --- | --- | --- | --- | --- |
| | | | | | | | | | | | | | |
| 2 |  |  | 5 |  |  | 8 |
| Depth of motif conservation (number of species) | | | | | | |

  
  

---

## >HOXB-AS3 TO HOXB\_XENOPUS (573 bases)

```
gtcata

gtcata  
Depth:5 (HOXB_XENOPUS)  
Ei-value:Undefined, Pi-value:Undefined  
Er-value:0.000, Pr-value:0.000  
No matches to eCLIP DataNo matches to TargetScan


gcgacttt

gcgacttt  
Depth:5 (HOXB_XENOPUS)  
Ei-value:Undefined, Pi-value:Undefined  
Er-value:0.000, Pr-value:0.000  
No matches to eCLIP DataNo matches to TargetScan

---------------------------------------------------------------------------------------------------------- 120  
 --------------------------------------------------||-------------------------------------------------------------------- 238  


gccacc

gccacc  
Depth:5 (HOXB_XENOPUS)  
Ei-value:Undefined, Pi-value:Undefined  
Er-value:0.000, Pr-value:0.000  
No matches to eCLIP DataNo matches to TargetScan

----------------------------------------------||------------------------------------------------------------------ 356  
 ------------------------------------------------------

taaact

taaact  
Depth:5 (HOXB_XENOPUS)  
Ei-value:Undefined, Pi-value:Undefined  
Er-value:0.000, Pr-value:0.000  
No matches to eCLIP DataNo matches to TargetScan

------------------------------------------------------------ 476  
 -------------------------------------------------------------------------------------------------                        573
```

|  |  |  |  |  |  |  |
| --- | --- | --- | --- | --- | --- | --- |
| | | | | | | | | | | | | | |
| 2 |  |  | 5 |  |  | 8 |
| Depth of motif conservation (number of species) | | | | | | |

  
  

---

## >HOXB-AS3 TO HOXB\_COELACANTH\_HOXB (573 bases)

```
 NO CONSERVED NODES FOUND  
------------------------------------------------------------------------------------------------------------------------ 120  
 --------------------------------------------------||-------------------------------------------------------------------- 238  
 ----------------------------------------------------||------------------------------------------------------------------ 356  
 ------------------------------------------------------------------------------------------------------------------------ 476  
 -------------------------------------------------------------------------------------------------                        573
```

|  |  |  |  |  |  |  |
| --- | --- | --- | --- | --- | --- | --- |
| | | | | | | | | | | | | | |
| 2 |  |  | 5 |  |  | 8 |
| Depth of motif conservation (number of species) | | | | | | |

  
  

---

## >HOXB-AS3 TO HOXB\_GAR (573 bases)

```
 NO CONSERVED NODES FOUND  
------------------------------------------------------------------------------------------------------------------------ 120  
 --------------------------------------------------||-------------------------------------------------------------------- 238  
 ----------------------------------------------------||------------------------------------------------------------------ 356  
 ------------------------------------------------------------------------------------------------------------------------ 476  
 -------------------------------------------------------------------------------------------------                        573
```

|  |  |  |  |  |  |  |
| --- | --- | --- | --- | --- | --- | --- |
| | | | | | | | | | | | | | |
| 2 |  |  | 5 |  |  | 8 |
| Depth of motif conservation (number of species) | | | | | | |

  
  

---

## >HOXB-AS3 TO HOXB\_SHARK (573 bases)

```
 NO CONSERVED NODES FOUND  
------------------------------------------------------------------------------------------------------------------------ 120  
 --------------------------------------------------||-------------------------------------------------------------------- 238  
 ----------------------------------------------------||------------------------------------------------------------------ 356  
 ------------------------------------------------------------------------------------------------------------------------ 476  
 -------------------------------------------------------------------------------------------------                        573
```

|  |  |  |  |  |  |  |
| --- | --- | --- | --- | --- | --- | --- |
| | | | | | | | | | | | | | |
| 2 |  |  | 5 |  |  | 8 |
| Depth of motif conservation (number of species) | | | | | | |

  
  

---
