# Supplementary material for: Highly conserved and cis-acting lncRNAs produced from paralogous regions in the center of HOXA and HOXB clusters in the endoderm lineage
Source: PLoS Genet. 2021 Jul 19;17(7):e1009681. doi: 10.1371/journal.pgen.1009681 (PMC8330917; doi:10.1371/journal.pgen.1009681)
Supplement: S1 Dataset — (ZIP) [file pgen.1009681.s015.zip › HOXB-AS3_var1/Html_Files/kmers_in_seqs_graded.html]

 MOTIF CONSERVATION

# MOTIF CONSERVATION

  

NAVIGATE ▼

▶HOXB-AS3▶HOXB\_DOG\_ISOFORM1▶HOXB5OS▶HOXB\_OPOSSUM▶HOXB\_XENOPUS▶HOXB\_COELACANTH\_HOXB▶HOXB\_GAR▶HOXB\_SHARK

  
  
  
  

## >HOXB-AS3 (573 bases)

```
gtcata

gtcata  
Depth:5 (HOXB_XENOPUS)  
Ei-value:Undefined, Pi-value:Undefined  
Er-value:0.000, Pr-value:0.000  
No matches to eCLIP DataNo matches to TargetScan


gcgacttt

gcgacttt  
Depth:5 (HOXB_XENOPUS)  
Ei-value:Undefined, Pi-value:Undefined  
Er-value:0.000, Pr-value:0.000  
No matches to eCLIP DataNo matches to TargetScan


tggg

gtcatagcgacttttggg  
Depth:4 (HOXB_OPOSSUM)  
Ei-value:Undefined, Pi-value:Undefined  
Er-value:0.000, Pr-value:0.000  
No matches to eCLIP DataNo matches to TargetScan


a

gtcatagcgacttttgggatagtttgctat  
Depth:2 (HOXB_DOG_ISOFORM1)  
Ei-value:Undefined, Pi-value:Undefined  
Er-value:0.000, Pr-value:0.000  
No matches to eCLIP DataNo matches to TargetScan


tagtttgct

tagtttgct  
Depth:4 (HOXB_OPOSSUM)  
Ei-value:Undefined, Pi-value:Undefined  
Er-value:0.000, Pr-value:0.000  
No matches to eCLIP DataNo matches to TargetScan


at

gtcatagcgacttttgggatagtttgctat  
Depth:2 (HOXB_DOG_ISOFORM1)  
Ei-value:Undefined, Pi-value:Undefined  
Er-value:0.000, Pr-value:0.000  
No matches to eCLIP DataNo matches to TargetScan

C

ga

gacaaaggg  
Depth:2 (HOXB_DOG_ISOFORM1)  
Ei-value:Undefined, Pi-value:Undefined  
Er-value:0.000, Pr-value:0.000  
No matches to eCLIP DataNo matches to TargetScan


caaaggg

caaaggg  
Depth:4 (HOXB_OPOSSUM)  
Ei-value:Undefined, Pi-value:Undefined  
Er-value:0.000, Pr-value:0.000  
No matches to eCLIP DataNo matches to TargetScan

A

gacaaagtca

gacaaagtca  
Depth:3 (HOXB5OS)  
Ei-value:Undefined, Pi-value:Undefined  
Er-value:0.000, Pr-value:0.000  
No matches to eCLIP DataNo matches to TargetScan


agggg

gacaaagtcaagggg  
Depth:2 (HOXB_DOG_ISOFORM1)  
Ei-value:Undefined, Pi-value:Undefined  
Er-value:0.000, Pr-value:0.000  
No matches to eCLIP DataNo matches to TargetScan

TGAAGGGA

aaggagg

aaggagg  
Depth:3 (HOXB5OS)  
Ei-value:Undefined, Pi-value:Undefined  
Er-value:0.000, Pr-value:0.000  
No matches to eCLIP DataNo matches to TargetScan


gcc

aaggagggcc  
Depth:2 (HOXB_DOG_ISOFORM1)  
Ei-value:Undefined, Pi-value:Undefined  
Er-value:0.000, Pr-value:0.000  
No matches to eCLIP DataNo matches to TargetScan

A

agtag

agtagagcctc  
Depth:2 (HOXB_DOG_ISOFORM1)  
Ei-value:Undefined, Pi-value:Undefined  
Er-value:0.000, Pr-value:0.000  
No matches to eCLIP DataMATCHES To TargetScan▶ miR-485-5p:GAGGCUG▶ miR-760:GGCUCUG


agcctc

agcctc  
Depth:3 (HOXB5OS)  
Ei-value:Undefined, Pi-value:Undefined  
Er-value:0.000, Pr-value:0.000  
No matches to eCLIP DataMATCHES To TargetScan▶ miR-485-5p:GAGGCUG

CACGACCCTCGGCTTC

ct

ctcctcaccagctcccc  
Depth:2 (HOXB_DOG_ISOFORM1)  
Ei-value:Undefined, Pi-value:Undefined  
Er-value:0.000, Pr-value:0.000  
No matches to eCLIP DataMATCHES To TargetScan▶ miR-1224-5p:UGAGGAC▶ miR-138-5p:GCUGGUG


cctcacca

cctcacca  
Depth:3 (HOXB5OS)  
Ei-value:Undefined, Pi-value:Undefined  
Er-value:0.000, Pr-value:0.000  
No matches to eCLIP DataNo matches to TargetScan


g

gctcccc  
Depth:3 (HOXB5OS)  
Ei-value:Undefined, Pi-value:Undefined  
Er-value:0.000, Pr-value:0.000  
No matches to eCLIP DataNo matches to TargetScan


ctcccc

ctcccc  
Depth:4 (HOXB_OPOSSUM)  
Ei-value:Undefined, Pi-value:Undefined  
Er-value:0.010, Pr-value:0.000  
No matches to eCLIP DataNo matches to TargetScan

C 120  
 TCCCT

ccaagtcc

ccaagtcc  
Depth:2 (HOXB_DOG_ISOFORM1)  
Ei-value:Undefined, Pi-value:Undefined  
Er-value:0.000, Pr-value:0.000  
No matches to eCLIP DataNo matches to TargetScan

A

gtaagaagtt

gtaagaagtt  
Depth:4 (HOXB_OPOSSUM)  
Ei-value:Undefined, Pi-value:Undefined  
Er-value:0.000, Pr-value:0.000  
No matches to eCLIP DataNo matches to TargetScan


gggcc

gtaagaagttgggcc  
Depth:3 (HOXB5OS)  
Ei-value:Undefined, Pi-value:Undefined  
Er-value:0.000, Pr-value:0.000  
No matches to eCLIP DataNo matches to TargetScan


a

gtaagaagttgggccaagctggaagggattgaccggccg  
Depth:2 (HOXB_DOG_ISOFORM1)  
Ei-value:Undefined, Pi-value:Undefined  
Er-value:0.000, Pr-value:0.000  
No matches to eCLIP DataMATCHES To TargetScan▶ miR-188-5p:AUCCCUU▶ miR-204-5p/211-5p:UCCCUUU▶ miR-328-3p:UGGCCCU


agctg

agctggaagggattgaccg  
Depth:3 (HOXB5OS)  
Ei-value:Undefined, Pi-value:Undefined  
Er-value:0.000, Pr-value:0.000  
No matches to eCLIP DataMATCHES To TargetScan▶ miR-188-5p:AUCCCUU▶ miR-204-5p/211-5p:UCCCUUU


gaaggga

gaaggga  
Depth:4 (HOXB_OPOSSUM)  
Ei-value:Undefined, Pi-value:Undefined  
Er-value:0.000, Pr-value:0.000  
No matches to eCLIP DataMATCHES To TargetScan▶ miR-204-5p/211-5p:UCCCUUU


ttgaccg

agctggaagggattgaccg  
Depth:3 (HOXB5OS)  
Ei-value:Undefined, Pi-value:Undefined  
Er-value:0.000, Pr-value:0.000  
No matches to eCLIP DataMATCHES To TargetScan▶ miR-188-5p:AUCCCUU▶ miR-204-5p/211-5p:UCCCUUU


g||ccg

gtaagaagttgggccaagctggaagggattgaccggccg  
Depth:2 (HOXB_DOG_ISOFORM1)  
Ei-value:Undefined, Pi-value:Undefined  
Er-value:0.000, Pr-value:0.000  
No matches to eCLIP DataMATCHES To TargetScan▶ miR-188-5p:AUCCCUU▶ miR-204-5p/211-5p:UCCCUUU▶ miR-328-3p:UGGCCCU

TTTCCTCTC

cctcgcc

cctcgcc  
Depth:2 (HOXB_DOG_ISOFORM1)  
Ei-value:Undefined, Pi-value:Undefined  
Er-value:0.000, Pr-value:0.000  
No matches to eCLIP DataNo matches to TargetScan


ggcctc

ggcctc  
Depth:4 (HOXB_OPOSSUM)  
Ei-value:Undefined, Pi-value:Undefined  
Er-value:0.010, Pr-value:0.000  
No matches to eCLIP DataNo matches to TargetScan

G

gcggagat

gcggagattccaggccc  
Depth:3 (HOXB5OS)  
Ei-value:Undefined, Pi-value:Undefined  
Er-value:0.000, Pr-value:0.000  
No matches to eCLIP DataMATCHES To TargetScan▶ miR-216a-5p:AAUCUCA▶ miR-216b-5p:AAUCUCU


tccaggc

tccaggc  
Depth:4 (HOXB_OPOSSUM)  
Ei-value:Undefined, Pi-value:Undefined  
Er-value:0.000, Pr-value:0.000  
No matches to eCLIP DataNo matches to TargetScan


cc

gcggagattccaggccc  
Depth:3 (HOXB5OS)  
Ei-value:Undefined, Pi-value:Undefined  
Er-value:0.000, Pr-value:0.000  
No matches to eCLIP DataMATCHES To TargetScan▶ miR-216a-5p:AAUCUCA▶ miR-216b-5p:AAUCUCU


t

gcggagattccaggccct  
Depth:2 (HOXB_DOG_ISOFORM1)  
Ei-value:Undefined, Pi-value:Undefined  
Er-value:0.000, Pr-value:0.000  
No matches to eCLIP DataMATCHES To TargetScan▶ miR-216a-5p:AAUCUCA▶ miR-216b-5p:AAUCUCU

ATAGAAACCA

ggacgtccct

ggacgtccct  
Depth:2 (HOXB_DOG_ISOFORM1)  
Ei-value:Undefined, Pi-value:Undefined  
Er-value:0.000, Pr-value:0.000  
No matches to eCLIP DataNo matches to TargetScan

T

agc

agcgccaccgcc  
Depth:3 (HOXB5OS)  
Ei-value:Undefined, Pi-value:Undefined  
Er-value:0.000, Pr-value:0.000  
No matches to eCLIP DataNo matches to TargetScan

 238  


agcgccaccgcc  
Depth:3 (HOXB5OS)  
Ei-value:Undefined, Pi-value:Undefined  
Er-value:0.000, Pr-value:0.000  
No matches to eCLIP DataNo matches to TargetScan


gccacc

gccacc  
Depth:5 (HOXB_XENOPUS)  
Ei-value:Undefined, Pi-value:Undefined  
Er-value:0.000, Pr-value:0.000  
No matches to eCLIP DataNo matches to TargetScan


gcc

agcgccaccgcc  
Depth:3 (HOXB5OS)  
Ei-value:Undefined, Pi-value:Undefined  
Er-value:0.000, Pr-value:0.000  
No matches to eCLIP DataNo matches to TargetScan

TCACATGCCAGTGCTGCCGGGAACCCAGCGATAT

ccgcacc

ccgcacc  
Depth:2 (HOXB_DOG_ISOFORM1)  
Ei-value:Undefined, Pi-value:Undefined  
Er-value:0.000, Pr-value:0.000  
No matches to eCLIP DataNo matches to TargetScan

AG||CGGAGAAGGTTC

caggctgc

caggctgc  
Depth:2 (HOXB_DOG_ISOFORM1)  
Ei-value:Undefined, Pi-value:Undefined  
Er-value:0.000, Pr-value:0.000  
No matches to eCLIP DataNo matches to TargetScan

CGGC

ggcggcgc

ggcggcgc  
Depth:2 (HOXB_DOG_ISOFORM1)  
Ei-value:Undefined, Pi-value:Undefined  
Er-value:0.000, Pr-value:0.000  
No matches to eCLIP DataNo matches to TargetScan

AGAGAGCGGGAAGAGAGGCTCGGAGGAAGCC

ccg

ccgggc  
Depth:2 (HOXB_DOG_ISOFORM1)  
Ei-value:Undefined, Pi-value:Undefined  
Er-value:0.090, Pr-value:0.010  
No matches to eCLIP DataNo matches to TargetScan

 356  


ggc

ccgggc  
Depth:2 (HOXB_DOG_ISOFORM1)  
Ei-value:Undefined, Pi-value:Undefined  
Er-value:0.090, Pr-value:0.010  
No matches to eCLIP DataNo matches to TargetScan

GTGGCGTGGTCAGGCTCCGA

gagcg

gagcggccgggatgcggccacacc  
Depth:2 (HOXB_DOG_ISOFORM1)  
Ei-value:Undefined, Pi-value:Undefined  
Er-value:0.000, Pr-value:0.000  
No matches to eCLIP DataMATCHES To TargetScan▶ miR-324-5p:GCAUCCC


gccggga

gccggga  
Depth:3 (HOXB5OS)  
Ei-value:Undefined, Pi-value:Undefined  
Er-value:0.000, Pr-value:0.000  
No matches to eCLIP DataNo matches to TargetScan


tgcgg

gagcggccgggatgcggccacacc  
Depth:2 (HOXB_DOG_ISOFORM1)  
Ei-value:Undefined, Pi-value:Undefined  
Er-value:0.000, Pr-value:0.000  
No matches to eCLIP DataMATCHES To TargetScan▶ miR-324-5p:GCAUCCC


ccacac

ccacac  
Depth:3 (HOXB5OS)  
Ei-value:Undefined, Pi-value:Undefined  
Er-value:0.000, Pr-value:0.000  
No matches to eCLIP DataNo matches to TargetScan


c

gagcggccgggatgcggccacacc  
Depth:2 (HOXB_DOG_ISOFORM1)  
Ei-value:Undefined, Pi-value:Undefined  
Er-value:0.000, Pr-value:0.000  
No matches to eCLIP DataMATCHES To TargetScan▶ miR-324-5p:GCAUCCC

GGCCT

gg

ggtaaact  
Depth:3 (HOXB5OS)  
Ei-value:Undefined, Pi-value:Undefined  
Er-value:0.000, Pr-value:0.000  
No matches to eCLIP DataNo matches to TargetScan


taaact

taaact  
Depth:5 (HOXB_XENOPUS)  
Ei-value:Undefined, Pi-value:Undefined  
Er-value:0.000, Pr-value:0.000  
No matches to eCLIP DataNo matches to TargetScan

CGCACCTCTTAGGATCTTGCTCCCGGACTCATTCCCT

tccccac

tccccac  
Depth:2 (HOXB_DOG_ISOFORM1)  
Ei-value:Undefined, Pi-value:Undefined  
Er-value:0.000, Pr-value:0.000  
No matches to eCLIP DataMATCHES To TargetScan▶ miR-491-5p:GUGGGGA

CCCCTATTTTAAAG

tt

ttttatttgg  
Depth:2 (HOXB_DOG_ISOFORM1)  
Ei-value:Undefined, Pi-value:Undefined  
Er-value:0.000, Pr-value:0.000  
No matches to eCLIP DataNo matches to TargetScan

 476  


ttatttgg

ttttatttgg  
Depth:2 (HOXB_DOG_ISOFORM1)  
Ei-value:Undefined, Pi-value:Undefined  
Er-value:0.000, Pr-value:0.000  
No matches to eCLIP DataNo matches to TargetScan

GTCGTCTGTATC

aatttagaa

aatttagaa  
Depth:3 (HOXB5OS)  
Ei-value:Undefined, Pi-value:Undefined  
Er-value:0.000, Pr-value:0.000  
No matches to eCLIP DataNo matches to TargetScan

C

gagataaa

gagataaa  
Depth:2 (HOXB_DOG_ISOFORM1)  
Ei-value:Undefined, Pi-value:Undefined  
Er-value:0.000, Pr-value:0.000  
No matches to eCLIP DataNo matches to TargetScan

TTAAGACAAAGAAAGTAAAATAAATCGAAATAAAATATAGGAATAGCTCTTGGCGAAAA                        573
```

|  |  |  |  |  |  |  |
| --- | --- | --- | --- | --- | --- | --- |
| | | | | | | | | | | | | | |
| 2 |  |  | 5 |  |  | 8 |
| Depth of motif conservation (number of species) | | | | | | |

  
  

---

  

## >HOXB\_DOG\_ISOFORM1 (2741 bases)

```
 TGAGGGGTATCTGTCTGACTTCTCGGCGATTTTTACGATCTAACTTCGAGATAAAACCCCTATCCATTTGACATCTAAAT

gtcata

gtcata  
Depth:5 (HOXB_XENOPUS)  
Ei-value:Undefined, Pi-value:Undefined  
Er-value:0.000, Pr-value:0.000  
No matches to TargetScan


gcgacttt

gcgacttt  
Depth:5 (HOXB_XENOPUS)  
Ei-value:Undefined, Pi-value:Undefined  
Er-value:0.000, Pr-value:0.000  
No matches to TargetScan


tggg

gtcatagcgacttttggg  
Depth:4 (HOXB_OPOSSUM)  
Ei-value:Undefined, Pi-value:Undefined  
Er-value:0.000, Pr-value:0.000  
No matches to TargetScan


a

gtcatagcgacttttgggatagtttgctat  
Depth:2 (HOXB_DOG_ISOFORM1)  
Ei-value:Undefined, Pi-value:Undefined  
Er-value:0.000, Pr-value:0.000  
No matches to TargetScan


tagtttgct

tagtttgct  
Depth:4 (HOXB_OPOSSUM)  
Ei-value:Undefined, Pi-value:Undefined  
Er-value:0.000, Pr-value:0.000  
No matches to TargetScan


at

gtcatagcgacttttgggatagtttgctat  
Depth:2 (HOXB_DOG_ISOFORM1)  
Ei-value:Undefined, Pi-value:Undefined  
Er-value:0.000, Pr-value:0.000  
No matches to TargetScan

G

ga

gacaaaggg  
Depth:2 (HOXB_DOG_ISOFORM1)  
Ei-value:Undefined, Pi-value:Undefined  
Er-value:0.000, Pr-value:0.000  
No matches to TargetScan


caaaggg

caaaggg  
Depth:4 (HOXB_OPOSSUM)  
Ei-value:Undefined, Pi-value:Undefined  
Er-value:0.000, Pr-value:0.000  
No matches to TargetScan

 120  


caaaggg  
Depth:4 (HOXB_OPOSSUM)  
Ei-value:Undefined, Pi-value:Undefined  
Er-value:0.000, Pr-value:0.000  
No matches to TargetScan

G

gacaaagtca

gacaaagtca  
Depth:3 (HOXB5OS)  
Ei-value:Undefined, Pi-value:Undefined  
Er-value:0.000, Pr-value:0.000  
No matches to TargetScan


agggg

gacaaagtcaagggg  
Depth:2 (HOXB_DOG_ISOFORM1)  
Ei-value:Undefined, Pi-value:Undefined  
Er-value:0.000, Pr-value:0.000  
No matches to TargetScan

CGAGGGGG

aaggagg

aaggagg  
Depth:3 (HOXB5OS)  
Ei-value:Undefined, Pi-value:Undefined  
Er-value:0.000, Pr-value:0.000  
No matches to TargetScan


gcc

aaggagggcc  
Depth:2 (HOXB_DOG_ISOFORM1)  
Ei-value:Undefined, Pi-value:Undefined  
Er-value:0.000, Pr-value:0.000  
No matches to TargetScan

C

agtag

agtagagcctc  
Depth:2 (HOXB_DOG_ISOFORM1)  
Ei-value:Undefined, Pi-value:Undefined  
Er-value:0.000, Pr-value:0.000  
MATCHES To TargetScan▶ miR-485-5p:GAGGCUG▶ miR-760:GGCUCUG


agcctc

agcctc  
Depth:3 (HOXB5OS)  
Ei-value:Undefined, Pi-value:Undefined  
Er-value:0.000, Pr-value:0.000  
MATCHES To TargetScan▶ miR-485-5p:GAGGCUG

TACGATTCTTGGCTGT

ct

ctcctcaccagctcccc  
Depth:2 (HOXB_DOG_ISOFORM1)  
Ei-value:Undefined, Pi-value:Undefined  
Er-value:0.000, Pr-value:0.000  
MATCHES To TargetScan▶ miR-1224-5p:UGAGGAC▶ miR-138-5p:GCUGGUG


cctcacca

cctcacca  
Depth:3 (HOXB5OS)  
Ei-value:Undefined, Pi-value:Undefined  
Er-value:0.000, Pr-value:0.000  
No matches to TargetScan


g

gctcccc  
Depth:3 (HOXB5OS)  
Ei-value:Undefined, Pi-value:Undefined  
Er-value:0.000, Pr-value:0.000  
No matches to TargetScan


ctcccc

ctcccc  
Depth:4 (HOXB_OPOSSUM)  
Ei-value:Undefined, Pi-value:Undefined  
Er-value:0.010, Pr-value:0.000  
No matches to TargetScan

TCCCCC

ccaagtcc

ccaagtcc  
Depth:2 (HOXB_DOG_ISOFORM1)  
Ei-value:Undefined, Pi-value:Undefined  
Er-value:0.000, Pr-value:0.000  
No matches to TargetScan

T

gtaagaagtt

gtaagaagtt  
Depth:4 (HOXB_OPOSSUM)  
Ei-value:Undefined, Pi-value:Undefined  
Er-value:0.000, Pr-value:0.000  
No matches to TargetScan


gggcc

gtaagaagttgggcc  
Depth:3 (HOXB5OS)  
Ei-value:Undefined, Pi-value:Undefined  
Er-value:0.000, Pr-value:0.000  
No matches to TargetScan


a

gtaagaagttgggccaagctggaagggattgaccggccg  
Depth:2 (HOXB_DOG_ISOFORM1)  
Ei-value:Undefined, Pi-value:Undefined  
Er-value:0.000, Pr-value:0.000  
MATCHES To TargetScan▶ miR-188-5p:AUCCCUU▶ miR-204-5p/211-5p:UCCCUUU▶ miR-328-3p:UGGCCCU


agctg

agctggaagggattgaccg  
Depth:3 (HOXB5OS)  
Ei-value:Undefined, Pi-value:Undefined  
Er-value:0.000, Pr-value:0.000  
MATCHES To TargetScan▶ miR-188-5p:AUCCCUU▶ miR-204-5p/211-5p:UCCCUUU


gaagg

gaaggga  
Depth:4 (HOXB_OPOSSUM)  
Ei-value:Undefined, Pi-value:Undefined  
Er-value:0.000, Pr-value:0.000  
MATCHES To TargetScan▶ miR-204-5p/211-5p:UCCCUUU

 240  


ga

gaaggga  
Depth:4 (HOXB_OPOSSUM)  
Ei-value:Undefined, Pi-value:Undefined  
Er-value:0.000, Pr-value:0.000  
MATCHES To TargetScan▶ miR-204-5p/211-5p:UCCCUUU


ttgaccg

agctggaagggattgaccg  
Depth:3 (HOXB5OS)  
Ei-value:Undefined, Pi-value:Undefined  
Er-value:0.000, Pr-value:0.000  
MATCHES To TargetScan▶ miR-188-5p:AUCCCUU▶ miR-204-5p/211-5p:UCCCUUU


g||ccg

gtaagaagttgggccaagctggaagggattgaccggccg  
Depth:2 (HOXB_DOG_ISOFORM1)  
Ei-value:Undefined, Pi-value:Undefined  
Er-value:0.000, Pr-value:0.000  
MATCHES To TargetScan▶ miR-188-5p:AUCCCUU▶ miR-204-5p/211-5p:UCCCUUU▶ miR-328-3p:UGGCCCU

CCGCCT

cctcgcc

cctcgcc  
Depth:2 (HOXB_DOG_ISOFORM1)  
Ei-value:Undefined, Pi-value:Undefined  
Er-value:0.000, Pr-value:0.000  
No matches to TargetScan

CTCGAG

ggcctc

ggcctc  
Depth:4 (HOXB_OPOSSUM)  
Ei-value:Undefined, Pi-value:Undefined  
Er-value:0.010, Pr-value:0.000  
No matches to TargetScan

T

gcggagat

gcggagattccaggccc  
Depth:3 (HOXB5OS)  
Ei-value:Undefined, Pi-value:Undefined  
Er-value:0.000, Pr-value:0.000  
MATCHES To TargetScan▶ miR-216a-5p:AAUCUCA▶ miR-216b-5p:AAUCUCU


tccaggc

tccaggc  
Depth:4 (HOXB_OPOSSUM)  
Ei-value:Undefined, Pi-value:Undefined  
Er-value:0.000, Pr-value:0.000  
No matches to TargetScan


cc

gcggagattccaggccc  
Depth:3 (HOXB5OS)  
Ei-value:Undefined, Pi-value:Undefined  
Er-value:0.000, Pr-value:0.000  
MATCHES To TargetScan▶ miR-216a-5p:AAUCUCA▶ miR-216b-5p:AAUCUCU


t

gcggagattccaggccct  
Depth:2 (HOXB_DOG_ISOFORM1)  
Ei-value:Undefined, Pi-value:Undefined  
Er-value:0.000, Pr-value:0.000  
MATCHES To TargetScan▶ miR-216a-5p:AAUCUCA▶ miR-216b-5p:AAUCUCU

CCAGAGACCC

ggacgtccct

ggacgtccct  
Depth:2 (HOXB_DOG_ISOFORM1)  
Ei-value:Undefined, Pi-value:Undefined  
Er-value:0.000, Pr-value:0.000  
No matches to TargetScan

C

agc

agcgccaccgcc  
Depth:3 (HOXB5OS)  
Ei-value:Undefined, Pi-value:Undefined  
Er-value:0.000, Pr-value:0.000  
No matches to TargetScan


gccacc

gccacc  
Depth:5 (HOXB_XENOPUS)  
Ei-value:Undefined, Pi-value:Undefined  
Er-value:0.000, Pr-value:0.000  
No matches to TargetScan


gcc

agcgccaccgcc  
Depth:3 (HOXB5OS)  
Ei-value:Undefined, Pi-value:Undefined  
Er-value:0.000, Pr-value:0.000  
No matches to TargetScan

CCTGTGCCAATGCCGCTGGGAAACCGCC 358  
 GAGAC

ccgcacc

ccgcacc  
Depth:2 (HOXB_DOG_ISOFORM1)  
Ei-value:Undefined, Pi-value:Undefined  
Er-value:0.000, Pr-value:0.000  
No matches to TargetScan

GGGCAAGAGAACTGCAGGGGGCCAACTGGGGGAGGCCGGAACGAGGGAGGGAAGGGGGGAGGGGGCGCACACCCACAGAGGCCAGAGCGACCGGCTTCTGGGCGCCCT 478  
 GAAGCCCAGACACCCCTATGCTGTCTTTCGCAGCCCCCTCTCTGGAGCCTCCTCTCTCAGCTTAAAAGGGCGACTTAGAGCTCGCCTCCGTGGCCCCTTTCGGTTCCTCCTCCCATCTTC 598  
 CCTGCCTTGGCTCCCCCTCCAGGAAGCAGCCACCCCTCTCTCTTTCTCAGCCACCGAATTTCCTGGGAAGCCTCTCCCTGCAGCGCCCAGAGATGGGGTTCAGCCCCGTCTTGGGGTGGG 718  
 GGGAGAGGGAGACAAACCTAAGCCCCACCCCCTCCAAAATCGATGCAAAAGAATAAGAATTGTAGAATCTCCCTTACCTTTGTAGACCTTGGGATTTCTTCCCTCCGATTGAAAGGGGTG 838  
 AAGAGGAAGGGGGGGGGGAAGCGTTCTGTTTCTTCTTCATCTTCCAGATAATATTGTCCCAACCTGAGAGCCGTCGCTTCCCCTTCTCTGACTTGGGAGAGAGGGTGTTTCGCTTTTCTG 958  
 ATATTTGCATAGAAAATGGAGTAAGTTCTGGCTTTGAAAAATGGTAT

caggctgc

caggctgc  
Depth:2 (HOXB_DOG_ISOFORM1)  
Ei-value:Undefined, Pi-value:Undefined  
Er-value:0.000, Pr-value:0.000  
No matches to TargetScan

AGTCCTGGGCAGATTGTCCATTTACCCTCCATGATGACTAAAATCCCAGATCAGCAAGCAAATCA 1078  
 ACTTAGAAATTAAAATTATTTTTGGTTCTCTCTTTTGTGTAATTCCCACCCCTCCTTCTTTCTCTCTTTCTCTCTCTCTCTCTCTCTCTCTCTCTCTCTCCCTCCTTTCCCTCTCTCTTC 1198  
 CTCTCTCTCTTTTGCTTTATCGAGCTGATATTGAAGTATAAAAATATCAAGAGGCTGGAGCCCTGAAGGACGACAGTGCTCCGACCTAGGTGTGGTGTCCAAAAGAATGCTGCATTATAA 1318  
 CCACCAGGGAAATGATAAAAAGTTCATGTTCACGATCGCCCGGCCACATGACCGGCGCCGGCCAATCGCTGGATTCAACCACTCATAAACTTCTATCACAAAGTTGTAAATTTTCATAAA 1438  
 ACAACAAGGAATTTATTGCATTTCTTCATGGCTGCTCCACCAGCAACCCTTTTCTCGGTCGCCATCTTCTTTTCTTCTCCTTCTCGCTATTTGGGGAAACCCCAATCTGAGAAGGGATGA 1558  
 GATTTGGGGTGCAGGGAATCAGACTGAGGAGCCACAGGGGTCCATCTGGAGAGGGGATTCTCTCCAGAACCTCCCTATTCTCACAGGAGCTCTGCCTCCCTCTTCTAGTAAGAATTCACC 1678  
 CCAAATCCTCCACACAGGGGCCATTCTAAGGCAGTTTTGGGGTGAGTCGGGAGTAACAGCGGGTATCCAAAATTGCCACTTGCATCAAGGCAAAATGAAGGCTTCAGGGTCCCCTCTCCT 1798  
 CCTCTGAATGCCCCCCTCATTCCCAGCCCTCTAGATGGTGGGACCCCCACCTGTGAGGCGTGTAGCATTGCGGGTGTGGGGGTCTTAGATCCCCTAGGGCCTAGTTTTCACCTAGGAAAG 1918  
 AAAGGCGGCCCTGACAGAGGCCAAGGTGGACAGCCTAGCAACCAGAGTCTCGCCTCTGCACCCCAGAAACTGGAAGAGGGTGAAGCCAGATCCCTGCCTCTTCCACCCAAATGCCCACAT 2038  
 GCTCAGGTTCCCAATGACAGCTCCATGTGCTCTCCAGGGGGTTCTGCGGTCATTAATGGGGGAAAGTGCCGGTTCAGGGGGACTTTTACCTACAAATTCAAATTAAAGAGATTCTCCTAA 2158  
 AACGCGCTTCCATTCAAACCTGCTTCTTTCTTTCTGTCCCAAAACCTGTTCTTTGCATTTAAATAATCCAAGGTCTGTAATCGAATAAATTCTAAAAATCAGCTCCACAGGTCTAGATGC 2278  
 GTAGGCACCGTCCTCCAGGTTTATGTTCCTCAATCAAGGGGTGTTTTGGAGGAAAAAACCCTACAAAACAAACAAAAAAATCTCCACCGCCACCAAACAGAATTCACCCCGTGTTTCCCA 2398  
 GGGCCCCTGAAATACTGCTGTATTTTGAATTGAGCTACCAGCGCCCAAGTGCAGGAAACGAAATAACATCCCCCAAAAAACGTTTTCTCTTTTTTCTCTCTTTCTCTGTATTCCCGAGGA 2518  
 GATTCCAGGTTGCCGGCAGCGCGGACCGCGGGCAGCAAGGCGCGGAGGGAGCCCGCGGAGCTTGGGCG

ggcggcgc

ggcggcgc  
Depth:2 (HOXB_DOG_ISOFORM1)  
Ei-value:Undefined, Pi-value:Undefined  
Er-value:0.000, Pr-value:0.000  
No matches to TargetScan

GG

ccgggc

ccgggc  
Depth:2 (HOXB_DOG_ISOFORM1)  
Ei-value:Undefined, Pi-value:Undefined  
Er-value:0.090, Pr-value:0.010  
No matches to TargetScan

TCC

gagcg

gagcggccgggatgcggccacacc  
Depth:2 (HOXB_DOG_ISOFORM1)  
Ei-value:Undefined, Pi-value:Undefined  
Er-value:0.000, Pr-value:0.000  
MATCHES To TargetScan▶ miR-324-5p:GCAUCCC


gccggga

gccggga  
Depth:3 (HOXB5OS)  
Ei-value:Undefined, Pi-value:Undefined  
Er-value:0.000, Pr-value:0.000  
No matches to TargetScan


tgcgg

gagcggccgggatgcggccacacc  
Depth:2 (HOXB_DOG_ISOFORM1)  
Ei-value:Undefined, Pi-value:Undefined  
Er-value:0.000, Pr-value:0.000  
MATCHES To TargetScan▶ miR-324-5p:GCAUCCC


ccacac

ccacac  
Depth:3 (HOXB5OS)  
Ei-value:Undefined, Pi-value:Undefined  
Er-value:0.000, Pr-value:0.000  
No matches to TargetScan


c

gagcggccgggatgcggccacacc  
Depth:2 (HOXB_DOG_ISOFORM1)  
Ei-value:Undefined, Pi-value:Undefined  
Er-value:0.000, Pr-value:0.000  
MATCHES To TargetScan▶ miR-324-5p:GCAUCCC

AGCCG

gg

ggtaaact  
Depth:3 (HOXB5OS)  
Ei-value:Undefined, Pi-value:Undefined  
Er-value:0.000, Pr-value:0.000  
No matches to TargetScan


ta

taaact  
Depth:5 (HOXB_XENOPUS)  
Ei-value:Undefined, Pi-value:Undefined  
Er-value:0.000, Pr-value:0.000  
No matches to TargetScan

 2638  


aact

taaact  
Depth:5 (HOXB_XENOPUS)  
Ei-value:Undefined, Pi-value:Undefined  
Er-value:0.000, Pr-value:0.000  
No matches to TargetScan

TGCACCGCTCAGACTCTCGTTCTCAGGGCTCATCCACTCCCCCACC

tccccac

tccccac  
Depth:2 (HOXB_DOG_ISOFORM1)  
Ei-value:Undefined, Pi-value:Undefined  
Er-value:0.000, Pr-value:0.000  
MATCHES To TargetScan▶ miR-491-5p:GUGGGGA

GTAAAAA

ttttatttgg

ttttatttgg  
Depth:2 (HOXB_DOG_ISOFORM1)  
Ei-value:Undefined, Pi-value:Undefined  
Er-value:0.000, Pr-value:0.000  
No matches to TargetScan

ATCGCTGTGTT

aatttagaa

aatttagaa  
Depth:3 (HOXB5OS)  
Ei-value:Undefined, Pi-value:Undefined  
Er-value:0.000, Pr-value:0.000  
No matches to TargetScan

T

gagataaa

gagataaa  
Depth:2 (HOXB_DOG_ISOFORM1)  
Ei-value:Undefined, Pi-value:Undefined  
Er-value:0.000, Pr-value:0.000  
No matches to TargetScan

2741
```

|  |  |  |  |  |  |  |
| --- | --- | --- | --- | --- | --- | --- |
| | | | | | | | | | | | | | |
| 2 |  |  | 5 |  |  | 8 |
| Depth of motif conservation (number of species) | | | | | | |

  
  

---

  

## >HOXB5OS (596 bases)

```
 AT

gtcata

gtcata  
Depth:5 (HOXB_XENOPUS)  
Ei-value:Undefined, Pi-value:Undefined  
Er-value:0.000, Pr-value:0.000  
No matches to TargetScan


gcgacttt

gcgacttt  
Depth:5 (HOXB_XENOPUS)  
Ei-value:Undefined, Pi-value:Undefined  
Er-value:0.000, Pr-value:0.000  
No matches to TargetScan


tggg

gtcatagcgacttttggg  
Depth:4 (HOXB_OPOSSUM)  
Ei-value:Undefined, Pi-value:Undefined  
Er-value:0.000, Pr-value:0.000  
No matches to TargetScan

G

tagtttgct

tagtttgct  
Depth:4 (HOXB_OPOSSUM)  
Ei-value:Undefined, Pi-value:Undefined  
Er-value:0.000, Pr-value:0.000  
No matches to TargetScan

TTCGG

caaaggg

caaaggg  
Depth:4 (HOXB_OPOSSUM)  
Ei-value:Undefined, Pi-value:Undefined  
Er-value:0.000, Pr-value:0.000  
No matches to TargetScan

G

gacaaagtca

gacaaagtca  
Depth:3 (HOXB5OS)  
Ei-value:Undefined, Pi-value:Undefined  
Er-value:0.000, Pr-value:0.000  
No matches to TargetScan

TGGGGTGAGAGGG

aaggagg

aaggagg  
Depth:3 (HOXB5OS)  
Ei-value:Undefined, Pi-value:Undefined  
Er-value:0.000, Pr-value:0.000  
No matches to TargetScan

ACCCAAAAC

agcctc

agcctc  
Depth:3 (HOXB5OS)  
Ei-value:Undefined, Pi-value:Undefined  
Er-value:0.000, Pr-value:0.000  
MATCHES To TargetScan▶ miR-485-5p:GAGGCUG

CAAGAC

cctcacca

cctcacca  
Depth:3 (HOXB5OS)  
Ei-value:Undefined, Pi-value:Undefined  
Er-value:0.000, Pr-value:0.000  
No matches to TargetScan

TCTCTTCACCG

g

gctcccc  
Depth:3 (HOXB5OS)  
Ei-value:Undefined, Pi-value:Undefined  
Er-value:0.000, Pr-value:0.000  
No matches to TargetScan


ctcccc

ctcccc  
Depth:4 (HOXB_OPOSSUM)  
Ei-value:Undefined, Pi-value:Undefined  
Er-value:0.010, Pr-value:0.000  
No matches to TargetScan

 120  


ctcccc  
Depth:4 (HOXB_OPOSSUM)  
Ei-value:Undefined, Pi-value:Undefined  
Er-value:0.010, Pr-value:0.000  
No matches to TargetScan

CTCCCCCCCCACAGGTCCT

gtaagaagtt

gtaagaagtt  
Depth:4 (HOXB_OPOSSUM)  
Ei-value:Undefined, Pi-value:Undefined  
Er-value:0.000, Pr-value:0.000  
No matches to TargetScan


gggcc

gtaagaagttgggcc  
Depth:3 (HOXB5OS)  
Ei-value:Undefined, Pi-value:Undefined  
Er-value:0.000, Pr-value:0.000  
No matches to TargetScan

C

agctg

agctggaagggattgaccg  
Depth:3 (HOXB5OS)  
Ei-value:Undefined, Pi-value:Undefined  
Er-value:0.000, Pr-value:0.000  
MATCHES To TargetScan▶ miR-188-5p:AUCCCUU▶ miR-204-5p/211-5p:UCCCUUU


gaaggga

gaaggga  
Depth:4 (HOXB_OPOSSUM)  
Ei-value:Undefined, Pi-value:Undefined  
Er-value:0.000, Pr-value:0.000  
MATCHES To TargetScan▶ miR-204-5p/211-5p:UCCCUUU


ttgaccg||

agctggaagggattgaccg  
Depth:3 (HOXB5OS)  
Ei-value:Undefined, Pi-value:Undefined  
Er-value:0.000, Pr-value:0.000  
MATCHES To TargetScan▶ miR-188-5p:AUCCCUU▶ miR-204-5p/211-5p:UCCCUUU

ACGCCTCTTCGCCCTAGGCT

ggcctc

ggcctc  
Depth:4 (HOXB_OPOSSUM)  
Ei-value:Undefined, Pi-value:Undefined  
Er-value:0.010, Pr-value:0.000  
No matches to TargetScan

T

gcggagat

gcggagattccaggccc  
Depth:3 (HOXB5OS)  
Ei-value:Undefined, Pi-value:Undefined  
Er-value:0.000, Pr-value:0.000  
MATCHES To TargetScan▶ miR-216a-5p:AAUCUCA▶ miR-216b-5p:AAUCUCU


tccaggc

tccaggc  
Depth:4 (HOXB_OPOSSUM)  
Ei-value:Undefined, Pi-value:Undefined  
Er-value:0.000, Pr-value:0.000  
No matches to TargetScan


cc

gcggagattccaggccc  
Depth:3 (HOXB5OS)  
Ei-value:Undefined, Pi-value:Undefined  
Er-value:0.000, Pr-value:0.000  
MATCHES To TargetScan▶ miR-216a-5p:AAUCUCA▶ miR-216b-5p:AAUCUCU

CACAGAGACCAGGACTTCCC 238  
 TC

agc

agcgccaccgcc  
Depth:3 (HOXB5OS)  
Ei-value:Undefined, Pi-value:Undefined  
Er-value:0.000, Pr-value:0.000  
No matches to TargetScan


gccacc

gccacc  
Depth:5 (HOXB_XENOPUS)  
Ei-value:Undefined, Pi-value:Undefined  
Er-value:0.000, Pr-value:0.000  
No matches to TargetScan


gcc

agcgccaccgcc  
Depth:3 (HOXB5OS)  
Ei-value:Undefined, Pi-value:Undefined  
Er-value:0.000, Pr-value:0.000  
No matches to TargetScan

CCTCGTGCCAATGCA

gccggga

gccggga  
Depth:3 (HOXB5OS)  
Ei-value:Undefined, Pi-value:Undefined  
Er-value:0.000, Pr-value:0.000  
No matches to TargetScan

AATCGCCATTAC

ccacac

ccacac  
Depth:3 (HOXB5OS)  
Ei-value:Undefined, Pi-value:Undefined  
Er-value:0.000, Pr-value:0.000  
No matches to TargetScan

TGGGCAAGAGAAACCGCACGGGGGCAATCGGAGAGGCCAGGAAAAGGGAGAGATGGAGGGAG||AA 356  
 GGTTCCAGGTTGGCCGTACTCAGGAGCAGGCCGAACGGCCCAGAGGAAGCCCTGGCGTCCGGTGCTGCCAGGCTCACTGCGGACGCACCCCAGCCC

gg

ggtaaact  
Depth:3 (HOXB5OS)  
Ei-value:Undefined, Pi-value:Undefined  
Er-value:0.000, Pr-value:0.000  
No matches to TargetScan


taaact

taaact  
Depth:5 (HOXB_XENOPUS)  
Ei-value:Undefined, Pi-value:Undefined  
Er-value:0.000, Pr-value:0.000  
No matches to TargetScan

CACTGTCGCTCGCCGG 476  
 GACTGGTTTCCCTTCAATTTAAAAATTTCATGTGGATCGCCCATGCC

aatttagaa

aatttagaa  
Depth:3 (HOXB5OS)  
Ei-value:Undefined, Pi-value:Undefined  
Er-value:0.000, Pr-value:0.000  
No matches to TargetScan

TGAAATAGATTTAAATACATAAAGTAAAAATAAATTGAAATAAAAGCAAGGCATCTGATGAAAA 596  
                                                                                                                          596
```

|  |  |  |  |  |  |  |
| --- | --- | --- | --- | --- | --- | --- |
| | | | | | | | | | | | | | |
| 2 |  |  | 5 |  |  | 8 |
| Depth of motif conservation (number of species) | | | | | | |

  
  

---

  

## >HOXB\_OPOSSUM (1381 bases)

```
 ATCTAACCCCCAAGCCCCCATTGCCCTAAACACGCAGCGAAGAGAGGGAGAGGAACCGGACTTACTCTGGAGAACGCTGGACCTCGGGCCTCCCTCCGTTGTCTCCGCCGCCGCCGCCGC 120  
 CACCGTCGCTGCCACCTCTGCTTTTGCTGCTGCTGAGGCCGGGCCCTCAGCTCCTCTCAGCTGGGAGAGAGCTCACCTACCTCTGCCCCCTTCCCCGCCACTGCGAGCAGTTAAAGTGTC 240  
 ACTTACATTCTCGAGAATGTGAAATATACCGCGCGGTGTCAACTCCCCAAAACCATAAAACTAACTTTATGGACCTCACGTGACTTTCTCGAGCCAGTGAGGGGTATCTGTCTGACTTCT 360  
 CGGCGATTTTTACGATCTAACTTCGAGATAAAACCCCTATCCATTTGACATCTAAAT

gtcata

gtcata  
Depth:5 (HOXB_XENOPUS)  
Ei-value:Undefined, Pi-value:Undefined  
Er-value:0.000, Pr-value:0.000  
No matches to TargetScan


gcgacttt

gcgacttt  
Depth:5 (HOXB_XENOPUS)  
Ei-value:Undefined, Pi-value:Undefined  
Er-value:0.000, Pr-value:0.000  
No matches to TargetScan


tggg

gtcatagcgacttttggg  
Depth:4 (HOXB_OPOSSUM)  
Ei-value:Undefined, Pi-value:Undefined  
Er-value:0.000, Pr-value:0.000  
No matches to TargetScan

A

tagtttgct

tagtttgct  
Depth:4 (HOXB_OPOSSUM)  
Ei-value:Undefined, Pi-value:Undefined  
Er-value:0.000, Pr-value:0.000  
No matches to TargetScan

ATCGATAAAGGAAAAAAAATAAAGGGGGGGAGGGG 480  


caaaggg

caaaggg  
Depth:4 (HOXB_OPOSSUM)  
Ei-value:Undefined, Pi-value:Undefined  
Er-value:0.000, Pr-value:0.000  
No matches to TargetScan

GGGCGAACAGACACTCCTGGACTTTCGACCGGTCGCCTCCCTAGCT

ctcccc

ctcccc  
Depth:4 (HOXB_OPOSSUM)  
Ei-value:Undefined, Pi-value:Undefined  
Er-value:0.010, Pr-value:0.000  
No matches to TargetScan

CCCACCCCCCAAGTCCT

gtaagaagtt

gtaagaagtt  
Depth:4 (HOXB_OPOSSUM)  
Ei-value:Undefined, Pi-value:Undefined  
Er-value:0.000, Pr-value:0.000  
No matches to TargetScan

TGGCTAA

gaaggga

gaaggga  
Depth:4 (HOXB_OPOSSUM)  
Ei-value:Undefined, Pi-value:Undefined  
Er-value:0.000, Pr-value:0.000  
MATCHES To TargetScan▶ miR-204-5p/211-5p:UCCCUUU

GAGATTGAGCTGG||CCAGT 598  
 TCCCCAACCCTTACACGGAGTCCCTGCTCCTGCTGCCCTTCTTAGAACCAG

ggcctc

ggcctc  
Depth:4 (HOXB_OPOSSUM)  
Ei-value:Undefined, Pi-value:Undefined  
Er-value:0.010, Pr-value:0.000  
No matches to TargetScan

CAGGGATCTTTCCGATCCAGCTTGTTTCCCGTGCCAACTCCACCTGGACGCCTTGAAAACCCA 718  
 GCGTCAGACAAGAAAACTCCACTGGCACAATTGAGGCGGGGGTGAAGAAGGACTTGAACCCTCAGAGGCTAGAGAG||ATGTAAGGGAATACGACCATGCTGGGGACCATAAGGAACAGA 836  
 ACTAAAGAAGTGGGGAGCCACTAGGAGCACGGAGGCCCTCCCCGACTGACGCATTGCGGTTGCCTCCTGGTCTAAGGAA

tccaggc

tccaggc  
Depth:4 (HOXB_OPOSSUM)  
Ei-value:Undefined, Pi-value:Undefined  
Er-value:0.000, Pr-value:0.000  
No matches to TargetScan

ACCAACTGACCTCTTCAAACCCCAAGTTTACCAA 956  
 T

gccacc

gccacc  
Depth:5 (HOXB_XENOPUS)  
Ei-value:Undefined, Pi-value:Undefined  
Er-value:0.000, Pr-value:0.000  
No matches to TargetScan

TTAGGAATCACTTCTTCTCTTTTCTCTCCTTTTTCTTGCCCTCTGCTTTTCTTCTTTCTTTCTTAATTCCATTTACTCCCCCCCTCCCACCCCTTCTTCTCCACCTCTTCTAT 1076  
 CCGTGGAACCATAATGAAATCAAATAAAAGAAATTAGGGCCGAAAGAGAGCTTGCTCAGGAAGGCGGTTAATAAAGGGAAGCTGGTTTGAGGCACTTGATTTCCCCGTCTAATTGCCTTC 1196  
 ATTGGCTTTCCCTACTTTGATGTGGAACAGGATTTGGCGCTAATAAAATAAGGACTTTCGGTCTGGGTCTGCAA

taaact

taaact  
Depth:5 (HOXB_XENOPUS)  
Ei-value:Undefined, Pi-value:Undefined  
Er-value:0.000, Pr-value:0.000  
No matches to TargetScan

CATCTCCACACAAAATCAACCAAGTCTTCTTCCCTGCGTC 1316  
 TTCCTTTCCTAATGACCAGAGTGAAAAACATATATGTTATTTTCTTGCTGCTGAATTTTTTCAGT                                                        1381
```

|  |  |  |  |  |  |  |
| --- | --- | --- | --- | --- | --- | --- |
| | | | | | | | | | | | | | |
| 2 |  |  | 5 |  |  | 8 |
| Depth of motif conservation (number of species) | | | | | | |

  
  

---

  

## >HOXB\_XENOPUS (647 bases)

```
 TCTGGGCCATTTGTACCATGGAACTTCTACATAAAACCCAATCCATTTGACATGGAAAT

gtcata

gtcata  
Depth:5 (HOXB_XENOPUS)  
Ei-value:Undefined, Pi-value:Undefined  
Er-value:0.000, Pr-value:0.000  
No matches to TargetScan

CCTACTTGGGCTTTACTTTGCCCAAATCACCAAAGGGGACGGGGATGCCTTGGCT 120  
 TCACTCTGCTCTGGG||GGGAGCTGTATTTTAGCCTGATCGAATTTTCGTGGCTGCACAGAACAAACAGAAGAAAAGACTTTGCGGAACGGGGGGGAAAAAAAATGCAACAAAGTCATCT 238  
 GCTTTCCAGATTATTCGCCATAAACCACCCTCCCAATTCCCATGCCTGTAACTAAATAATCCGCTAGAAACGCAGCAAAACTGGTGTCATCTCCACAAAGTGTAAAAACCTGGAATTCAG 358  
 TGAGTAATT

gcgacttt

gcgacttt  
Depth:5 (HOXB_XENOPUS)  
Ei-value:Undefined, Pi-value:Undefined  
Er-value:0.000, Pr-value:0.000  
No matches to TargetScan


gccacc

gccacc  
Depth:5 (HOXB_XENOPUS)  
Ei-value:Undefined, Pi-value:Undefined  
Er-value:0.000, Pr-value:0.000  
No matches to TargetScan

TCTGTAACAGCATCAATTTGTCTGCCCTGCTCTGTGCAACACTTTCAGCTTCCCCCTTGGGTTACATAAGGGTGCAAAGAATCCCAATGATTGAGAA 478  
 AAGTTTATTTTCATTGCCAATGTGTTAACCTATTACATCATGGTTTTTATCA

taaact

taaact  
Depth:5 (HOXB_XENOPUS)  
Ei-value:Undefined, Pi-value:Undefined  
Er-value:0.000, Pr-value:0.000  
No matches to TargetScan

GCTCCGACCCTGTGTAAATAAATGCTTCTTAACGAGTGTGGGTGCTTATGATTTCCTCATTA 598  
 ACAACCTTGTTATACTCCAATTTACAGTATTTATATTTGAACGTGTCAG                                                                        647
```

|  |  |  |  |  |  |  |
| --- | --- | --- | --- | --- | --- | --- |
| | | | | | | | | | | | | | |
| 2 |  |  | 5 |  |  | 8 |
| Depth of motif conservation (number of species) | | | | | | |

  
  

---

  

## >HOXB\_COELACANTH\_HOXB (974 bases)

```
 NO CONSERVED NODES FOUND  
AAAACACTAATACTAAGCTTTTCAACTGCATGTCATGGACGATTTACAATTTTTGGAGAATAAGAAAGTTTAGAACTCATTGGATTCATCTAAAAGTGCATGATATTTAATCGCCTAACG 120  
 GGATGACACATAGGTGAGTTTTGATTAAATAATTAGCTTGTTCAAATTTTGCATTGGAGTGTTGTGTGATTTAGCAACGCTCTGGTTGTCTCGTGCGAACTTAAATTTGTTCAGGCGGTA 240  
 GGAAAATAGATTCAATGGCCGGCAGCAAATTTTATCTAAAAGAGGATGCAATGACTGTGAACTTGGTCTGAAGCGGGCTCTGCTGGTGGTTTGTGGTGTTACAG||ATCCTAGAGAATCA 358  
 ATTGTTCTACTGTCGGTATACCATTAAAGAGAGGAAGCCAATGAAAAATGAATTTGAAGTCTTCGCCTTTCTACACG||GGTTTTTCCTTTGTATATGGCTTGAAATCAGACCTATCCAA 476  
 AGTTTGCATTATTCACGCCAAGAATGTGGAAAAAGAGAAAAAGGAAACCGAACTCTTGAAACGGTTTGAAAAGAAAACGCCCTTAACAAGAAGTAATATCCAATTTAGGGAGGGGGCAAC 596  
 TAAGCAGGAGAATAACTAACTGGGGACATTGCAAAAACGTTTATAAATACAATTGTATCTATTTTCAAGTTAAAAGAAACAAGAAAGCGTTTGAAGACAACCCACTCCCTTCTTATTGGA 716  
 GAATGTTAAAAGAAGTTTGAAACCCTTATATTTAACTGCGTGAGTATTTCTTCTATGCATTATATTTTCTTGTACTTATACTTTAATGTATTGGATTAATTGTATTTTTTTTATCTGTAA 836  
 ATACCTTTTATAGCAATAGTTAGATGATCAGCATTGCCTTTGTAACTATTGTTGACTTCAGAACTGGATTATCTAACCGCAGTTTTAGTTTCCTTTATTGCACTGTATTTAAAACATGGA 956  
 AAAATAAATTGAAATGAG                                                                                                       974
```

|  |  |  |  |  |  |  |
| --- | --- | --- | --- | --- | --- | --- |
| | | | | | | | | | | | | | |
| 2 |  |  | 5 |  |  | 8 |
| Depth of motif conservation (number of species) | | | | | | |

  
  

---

  

## >HOXB\_GAR (2133 bases)

```
 NO CONSERVED NODES FOUND  
TTTGGTATACTTTGGCAAAGGGTACAGCAAAATTTCAAAACGCGGGGAGGCAGAGAAAGGCAGATGGTTACATTTGGCTTGAGAGCGCCTCCACTCCACCCCGAGCTCTCAAAAGAAATG 120  
 TGGGAAGTATCTCAACAGTGGGGGATTAATTG||GGTTTTCCTTTGTGAAACCGGTCGGACTCCGAGCAGCACAAACACATTTTCGAACTCGCCCCCTGAAAGAAACCAAGCGACAAACT 238  
 GTTTGGGGAGGGAGGGGGGAATTCAGAAGCCAGTGTCGCCAGGAGGAAACTGGGACAAGCAGCGAGAGAGCCCGGAATCTCAGCGGAAACACCGCCGAGGAATCGCACCTACGAGAACAG 358  
 CGCTCTTTTAAAACTATTAGCCTTGTTTTACCGAGACAGAAAAACAAGTCGGGCATGCCTGTCATTTTGAGTAACTTCTCCGAAGATCGCTCACTGCTCTGTGTACATGCTAGATTTGTT 478  
 TATGTAAGTAAATACTCTATTCGTAAAATGAAATGTTTTTTGTGTTTGTATTTGCTCTGATAAATCACATTGATCAAGATTTATTCAGGGAACTGATAGCATTGTTCGAGGTTTATGCCA 598  
 AATATTTTAATGTATGAGGTCATAATTACGAAAATTATATATATATATGCTTAGAAAATGTGTATATAAGGGCTTGATGCGTTTTGCATAAGTTAATTACAGTAAGTCCGTGTTCCTAGG 718  
 TACAGCTTCATTACAATTATACTGAAAATCATTTAGGTCTCTTTCTGTCTGGGTTTTAAACCCATTCTAGCTTTTTTAGTGTAAAATAAAAATTAAAATGTGAAGAAATGAATTTTCATC 838  
 TTTTTTTTTAAAGGAACCAAAAACTACTGAGTACTCTCTGGCAAAGAAAGGTCAGAACTCCAAAGGAAACTTTTAAATTCAAAACAGACCGTAGCGACCCAAAGGAAAAAACAGACTGCG 958  
 ATTTATTTCAACTTTTTTTTTCGAAAATAGTTTCTTTGTATACTTCCTTAAGAATAAATAAGGAGCTCGGTAAGTGGACTCCATGTTACACTTAAAAATGTTTAAATAGATGCACCCACT 1078  
 GAATTAAAGTCGAAGGAGTCCCGCAAGAGCTCATAAAAAGACTTTTCGAGTTCCTCTCGCCTATTTTTCACAGCACAGATTTCAGCCTTTTTAAAACGTTTTAACAAAAAAAAGTTTGAG 1198  
 GCTAAGAGTCCGAATCAATCGTCATTTACAATTCTTAAAATTAAATGTTAAAACATTAACAAAATGGTGAGATGTTTCGTTACATATTCTTAGCTATTATAAATCTGAACTTTTAATTTT 1318  
 AAGCACATTAAACAGAAGGCTTTTCTTTTTTTTTTATTCGCTACCCCGAGATCTTTCCCCTCATTCACCCTGTGAGACTGTTGCACTGATAAAAATCGCCCAAGCAAGACAGTTTGAAAA 1438  
 CACACTTTCCGACTGTTTCCAAAGTCAGAGCTTAATTGTTACTTACGGCTTTCGCATGAGGTTTCTAAGCGCGGTGGGTCCTTATTTCTGCATTAACTCGAGTTTATTTTAAAAAAAATC 1558  
 AAATACAAGAAGAAAATAAACATCAGCAAATCGATAATTACTGTAACATCGCTCTCTCGCGCGCGGGGGGGGAAGCCGCCTGTATTTAATTACTCTATTCCACGTGAGATTGTTCTTAAT 1678  
 TATAATTTAAAAAGGCACCATAAGGTGAACAATTAATAAAAGAAAGGCTGTTTTCGTCGCTAAAACGGTACCAATCATAACGACAATATAGAGAAAGAATCACCCTATAATATTTAAACC 1798  
 GACGTGGCAGGCAGAGTGATTCTATGGATTAAATGAAGTAGGTAAAAGATAATATAAACTTGCCTTGTTGACTGAGGTTTATTTCTAATTATTACTCATCAATTACTTGATCCTCAAAGA 1918  
 CACTTAATTGCGCTGAACTCATCAGCTGGCCCGTTACCTCCTACAATTCTGCGCAGATTAGCCGGTAAATATTTAATTGCTAGGTGCGAGTTTGATCTCATTTACTCCCGTTTTAAACGC 2038  
 TGATGTGGAAAACTGTAGCAAAGGCCAGGAACAACTTAAATGTTCACTGCTTTGCCCACTAACTAATGATTAACTCGGCTGGGGTGGAAATCCGC                          2133
```

|  |  |  |  |  |  |  |
| --- | --- | --- | --- | --- | --- | --- |
| | | | | | | | | | | | | | |
| 2 |  |  | 5 |  |  | 8 |
| Depth of motif conservation (number of species) | | | | | | |

  
  

---

  

## >HOXB\_SHARK (912 bases)

```
 NO CONSERVED NODES FOUND  
GCCTCCTTTTTTATAGTTTGTCCCAAAGCAGACTGGGGTTAGTTCAAAAACTGCCTCAGATAGCACACATACACACACAGACAGAATGCACGGAGGAGCGGGTCCAGAGGAGCAGGACCT 120  
 CTGCGCCCTAAATGCGTCTGGGAGCAGAGACGCTGCAGCACATCAGCCGCTG||AAAAGTAAACGGTGGCCGGTCTTGGAGAATGGGACACCTGGAGCTTGCATTTTGGGGATTGACAGC 238  
 GGCGTGTCATCCACCCGTGCTCTGCGCTGCAGCTTCACAGGCGGAGAAATTAACCGGCAGGCTCCACTTTCAGAGGGTCCACAGACCTCTGCACGGCTGCGCAAGGTTTTCCTCCATCCT 358  
 CTTCTTCCTCTCTGTGACCTAAATGTTTTTAGTTTGTTCTTCTGTTCAAACAGTTACTCCGTCCGTTTGTCCTCTTCATCTGTACCATTCACTGCCGAGGAAGAGGAAGCTGTCGGGGAT 478  
 GAGTTCATCAGTTTGTTCTCCTTCTTCCACTTCATCCTCCGGTTCTGGAACCAGATCTTGATCTGGCGCTCCGTGAGACAGAGCGCATGGGCGATCTCGATGCGCCGCCGCCGCGTCAGG 598  
 TACCGGTTGAAGTGGAACTCCTTCTCCAGCTCCAGAGTCTGGTAGCGCGTGTACGTCTGCCGCCCGCGCCTGCCGGGGCTGCCGAAGGTGCCTGCGTGCGGGGACAGAAGTACAGAAGGA 718  
 AAGTTAACGTTTAACCCACCTCAAAACAAAAACTGTACGTGTTTGAGACAGAAACAAGCTAAGCCTGTATAATTTACAGTATTTCATTTTCTCTTTTATTTTATTTTATTTTTACCAGAC 838  
 GATGTATTTGCATTTAATAGAATAAATATTTATTTTAGATTGTGTTTAACATTTTTGCCGGTTGTGCAGTACTT                                               912
```

|  |  |  |  |  |  |  |
| --- | --- | --- | --- | --- | --- | --- |
| | | | | | | | | | | | | | |
| 2 |  |  | 5 |  |  | 8 |
| Depth of motif conservation (number of species) | | | | | | |

  
  

---
