# Supplementary material for: Highly conserved and cis-acting lncRNAs produced from paralogous regions in the center of HOXA and HOXB clusters in the endoderm lineage
Source: PLoS Genet. 2021 Jul 19;17(7):e1009681. doi: 10.1371/journal.pgen.1009681 (PMC8330917; doi:10.1371/journal.pgen.1009681)
Supplement: S1 Dataset — (ZIP) [file pgen.1009681.s015.zip › HOXB-AS3_var1/Html_Files/kmers_in_seqs_layer_conservation.html]

 MOTIF CONSERVATION

# MOTIF CONSERVATION

## Motifs mapped to anchor sequence

  

NAVIGATE ▼

▶HOXB\_DOG\_ISOFORM1▶HOXB5OS▶HOXB\_OPOSSUM▶HOXB\_XENOPUS▶HOXB\_COELACANTH\_HOXB▶HOXB\_GAR▶HOXB\_SHARK

  
  
  
  

## >HOXB-AS3 TO HOXB\_DOG\_ISOFORM1 (573 bases)

```
gtcata

gtcata  
Depth:5 (HOXB_XENOPUS)  
Ei-value:Undefined, Pi-value:Undefined  
Er-value:0.000, Pr-value:0.000  
No matches to eCLIP DataNo matches to TargetScan


gcgacttt

gcgacttt  
Depth:5 (HOXB_XENOPUS)  
Ei-value:Undefined, Pi-value:Undefined  
Er-value:0.000, Pr-value:0.000  
No matches to eCLIP DataNo matches to TargetScan


tggg

gtcatagcgacttttggg  
Depth:4 (HOXB_OPOSSUM)  
Ei-value:Undefined, Pi-value:Undefined  
Er-value:0.000, Pr-value:0.000  
No matches to eCLIP DataNo matches to TargetScan


a

gtcatagcgacttttgggatagtttgctat  
Depth:2 (HOXB_DOG_ISOFORM1)  
Ei-value:Undefined, Pi-value:Undefined  
Er-value:0.000, Pr-value:0.000  
No matches to eCLIP DataNo matches to TargetScan


tagtttgct

tagtttgct  
Depth:4 (HOXB_OPOSSUM)  
Ei-value:Undefined, Pi-value:Undefined  
Er-value:0.000, Pr-value:0.000  
No matches to eCLIP DataNo matches to TargetScan


at

gtcatagcgacttttgggatagtttgctat  
Depth:2 (HOXB_DOG_ISOFORM1)  
Ei-value:Undefined, Pi-value:Undefined  
Er-value:0.000, Pr-value:0.000  
No matches to eCLIP DataNo matches to TargetScan

C

ga

gacaaaggg  
Depth:2 (HOXB_DOG_ISOFORM1)  
Ei-value:Undefined, Pi-value:Undefined  
Er-value:0.000, Pr-value:0.000  
No matches to eCLIP DataNo matches to TargetScan


caaaggg

caaaggg  
Depth:4 (HOXB_OPOSSUM)  
Ei-value:Undefined, Pi-value:Undefined  
Er-value:0.000, Pr-value:0.000  
No matches to eCLIP DataNo matches to TargetScan

A

gacaaagtca

gacaaagtca  
Depth:3 (HOXB5OS)  
Ei-value:Undefined, Pi-value:Undefined  
Er-value:0.000, Pr-value:0.000  
No matches to eCLIP DataNo matches to TargetScan


agggg

gacaaagtcaagggg  
Depth:2 (HOXB_DOG_ISOFORM1)  
Ei-value:Undefined, Pi-value:Undefined  
Er-value:0.000, Pr-value:0.000  
No matches to eCLIP DataNo matches to TargetScan

TGAAGGGA

aaggagg

aaggagg  
Depth:3 (HOXB5OS)  
Ei-value:Undefined, Pi-value:Undefined  
Er-value:0.000, Pr-value:0.000  
No matches to eCLIP DataNo matches to TargetScan


gcc

aaggagggcc  
Depth:2 (HOXB_DOG_ISOFORM1)  
Ei-value:Undefined, Pi-value:Undefined  
Er-value:0.000, Pr-value:0.000  
No matches to eCLIP DataNo matches to TargetScan

A

agtag

agtagagcctc  
Depth:2 (HOXB_DOG_ISOFORM1)  
Ei-value:Undefined, Pi-value:Undefined  
Er-value:0.000, Pr-value:0.000  
No matches to eCLIP DataMATCHES To TargetScan▶ miR-485-5p:GAGGCUG▶ miR-760:GGCUCUG


agcctc

agcctc  
Depth:3 (HOXB5OS)  
Ei-value:Undefined, Pi-value:Undefined  
Er-value:0.000, Pr-value:0.000  
No matches to eCLIP DataMATCHES To TargetScan▶ miR-485-5p:GAGGCUG

CACGACCCTCGGCTTC

ct

ctcctcaccagctcccc  
Depth:2 (HOXB_DOG_ISOFORM1)  
Ei-value:Undefined, Pi-value:Undefined  
Er-value:0.000, Pr-value:0.000  
No matches to eCLIP DataMATCHES To TargetScan▶ miR-1224-5p:UGAGGAC▶ miR-138-5p:GCUGGUG


cctcacca

cctcacca  
Depth:3 (HOXB5OS)  
Ei-value:Undefined, Pi-value:Undefined  
Er-value:0.000, Pr-value:0.000  
No matches to eCLIP DataNo matches to TargetScan


g

gctcccc  
Depth:3 (HOXB5OS)  
Ei-value:Undefined, Pi-value:Undefined  
Er-value:0.000, Pr-value:0.000  
No matches to eCLIP DataNo matches to TargetScan


ctcccc

ctcccc  
Depth:4 (HOXB_OPOSSUM)  
Ei-value:Undefined, Pi-value:Undefined  
Er-value:0.010, Pr-value:0.000  
No matches to eCLIP DataNo matches to TargetScan

C 120  
 TCCCT

ccaagtcc

ccaagtcc  
Depth:2 (HOXB_DOG_ISOFORM1)  
Ei-value:Undefined, Pi-value:Undefined  
Er-value:0.000, Pr-value:0.000  
No matches to eCLIP DataNo matches to TargetScan

A

gtaagaagtt

gtaagaagtt  
Depth:4 (HOXB_OPOSSUM)  
Ei-value:Undefined, Pi-value:Undefined  
Er-value:0.000, Pr-value:0.000  
No matches to eCLIP DataNo matches to TargetScan


gggcc

gtaagaagttgggcc  
Depth:3 (HOXB5OS)  
Ei-value:Undefined, Pi-value:Undefined  
Er-value:0.000, Pr-value:0.000  
No matches to eCLIP DataNo matches to TargetScan


a

gtaagaagttgggccaagctggaagggattgaccggccg  
Depth:2 (HOXB_DOG_ISOFORM1)  
Ei-value:Undefined, Pi-value:Undefined  
Er-value:0.000, Pr-value:0.000  
No matches to eCLIP DataMATCHES To TargetScan▶ miR-188-5p:AUCCCUU▶ miR-204-5p/211-5p:UCCCUUU▶ miR-328-3p:UGGCCCU


agctg

agctggaagggattgaccg  
Depth:3 (HOXB5OS)  
Ei-value:Undefined, Pi-value:Undefined  
Er-value:0.000, Pr-value:0.000  
No matches to eCLIP DataMATCHES To TargetScan▶ miR-188-5p:AUCCCUU▶ miR-204-5p/211-5p:UCCCUUU


gaaggga

gaaggga  
Depth:4 (HOXB_OPOSSUM)  
Ei-value:Undefined, Pi-value:Undefined  
Er-value:0.000, Pr-value:0.000  
No matches to eCLIP DataMATCHES To TargetScan▶ miR-204-5p/211-5p:UCCCUUU


ttgaccg

agctggaagggattgaccg  
Depth:3 (HOXB5OS)  
Ei-value:Undefined, Pi-value:Undefined  
Er-value:0.000, Pr-value:0.000  
No matches to eCLIP DataMATCHES To TargetScan▶ miR-188-5p:AUCCCUU▶ miR-204-5p/211-5p:UCCCUUU


g||ccg

gtaagaagttgggccaagctggaagggattgaccggccg  
Depth:2 (HOXB_DOG_ISOFORM1)  
Ei-value:Undefined, Pi-value:Undefined  
Er-value:0.000, Pr-value:0.000  
No matches to eCLIP DataMATCHES To TargetScan▶ miR-188-5p:AUCCCUU▶ miR-204-5p/211-5p:UCCCUUU▶ miR-328-3p:UGGCCCU

TTTCCTCTC

cctcgcc

cctcgcc  
Depth:2 (HOXB_DOG_ISOFORM1)  
Ei-value:Undefined, Pi-value:Undefined  
Er-value:0.000, Pr-value:0.000  
No matches to eCLIP DataNo matches to TargetScan


ggcctc

ggcctc  
Depth:4 (HOXB_OPOSSUM)  
Ei-value:Undefined, Pi-value:Undefined  
Er-value:0.010, Pr-value:0.000  
No matches to eCLIP DataNo matches to TargetScan

G

gcggagat

gcggagattccaggccc  
Depth:3 (HOXB5OS)  
Ei-value:Undefined, Pi-value:Undefined  
Er-value:0.000, Pr-value:0.000  
No matches to eCLIP DataMATCHES To TargetScan▶ miR-216a-5p:AAUCUCA▶ miR-216b-5p:AAUCUCU


tccaggc

tccaggc  
Depth:4 (HOXB_OPOSSUM)  
Ei-value:Undefined, Pi-value:Undefined  
Er-value:0.000, Pr-value:0.000  
No matches to eCLIP DataNo matches to TargetScan


cc

gcggagattccaggccc  
Depth:3 (HOXB5OS)  
Ei-value:Undefined, Pi-value:Undefined  
Er-value:0.000, Pr-value:0.000  
No matches to eCLIP DataMATCHES To TargetScan▶ miR-216a-5p:AAUCUCA▶ miR-216b-5p:AAUCUCU


t

gcggagattccaggccct  
Depth:2 (HOXB_DOG_ISOFORM1)  
Ei-value:Undefined, Pi-value:Undefined  
Er-value:0.000, Pr-value:0.000  
No matches to eCLIP DataMATCHES To TargetScan▶ miR-216a-5p:AAUCUCA▶ miR-216b-5p:AAUCUCU

ATAGAAACCA

ggacgtccct

ggacgtccct  
Depth:2 (HOXB_DOG_ISOFORM1)  
Ei-value:Undefined, Pi-value:Undefined  
Er-value:0.000, Pr-value:0.000  
No matches to eCLIP DataNo matches to TargetScan

T

agc

agcgccaccgcc  
Depth:3 (HOXB5OS)  
Ei-value:Undefined, Pi-value:Undefined  
Er-value:0.000, Pr-value:0.000  
No matches to eCLIP DataNo matches to TargetScan

 238  


agcgccaccgcc  
Depth:3 (HOXB5OS)  
Ei-value:Undefined, Pi-value:Undefined  
Er-value:0.000, Pr-value:0.000  
No matches to eCLIP DataNo matches to TargetScan


gccacc

gccacc  
Depth:5 (HOXB_XENOPUS)  
Ei-value:Undefined, Pi-value:Undefined  
Er-value:0.000, Pr-value:0.000  
No matches to eCLIP DataNo matches to TargetScan


gcc

agcgccaccgcc  
Depth:3 (HOXB5OS)  
Ei-value:Undefined, Pi-value:Undefined  
Er-value:0.000, Pr-value:0.000  
No matches to eCLIP DataNo matches to TargetScan

TCACATGCCAGTGCTGCCGGGAACCCAGCGATAT

ccgcacc

ccgcacc  
Depth:2 (HOXB_DOG_ISOFORM1)  
Ei-value:Undefined, Pi-value:Undefined  
Er-value:0.000, Pr-value:0.000  
No matches to eCLIP DataNo matches to TargetScan

AG||CGGAGAAGGTTC

caggctgc

caggctgc  
Depth:2 (HOXB_DOG_ISOFORM1)  
Ei-value:Undefined, Pi-value:Undefined  
Er-value:0.000, Pr-value:0.000  
No matches to eCLIP DataNo matches to TargetScan

CGGC

ggcggcgc

ggcggcgc  
Depth:2 (HOXB_DOG_ISOFORM1)  
Ei-value:Undefined, Pi-value:Undefined  
Er-value:0.000, Pr-value:0.000  
No matches to eCLIP DataNo matches to TargetScan

AGAGAGCGGGAAGAGAGGCTCGGAGGAAGCC

ccg

ccgggc  
Depth:2 (HOXB_DOG_ISOFORM1)  
Ei-value:Undefined, Pi-value:Undefined  
Er-value:0.090, Pr-value:0.010  
No matches to eCLIP DataNo matches to TargetScan

 356  


ggc

ccgggc  
Depth:2 (HOXB_DOG_ISOFORM1)  
Ei-value:Undefined, Pi-value:Undefined  
Er-value:0.090, Pr-value:0.010  
No matches to eCLIP DataNo matches to TargetScan

GTGGCGTGGTCAGGCTCCGA

gagcg

gagcggccgggatgcggccacacc  
Depth:2 (HOXB_DOG_ISOFORM1)  
Ei-value:Undefined, Pi-value:Undefined  
Er-value:0.000, Pr-value:0.000  
No matches to eCLIP DataMATCHES To TargetScan▶ miR-324-5p:GCAUCCC


gccggga

gccggga  
Depth:3 (HOXB5OS)  
Ei-value:Undefined, Pi-value:Undefined  
Er-value:0.000, Pr-value:0.000  
No matches to eCLIP DataNo matches to TargetScan


tgcgg

gagcggccgggatgcggccacacc  
Depth:2 (HOXB_DOG_ISOFORM1)  
Ei-value:Undefined, Pi-value:Undefined  
Er-value:0.000, Pr-value:0.000  
No matches to eCLIP DataMATCHES To TargetScan▶ miR-324-5p:GCAUCCC


ccacac

ccacac  
Depth:3 (HOXB5OS)  
Ei-value:Undefined, Pi-value:Undefined  
Er-value:0.000, Pr-value:0.000  
No matches to eCLIP DataNo matches to TargetScan


c

gagcggccgggatgcggccacacc  
Depth:2 (HOXB_DOG_ISOFORM1)  
Ei-value:Undefined, Pi-value:Undefined  
Er-value:0.000, Pr-value:0.000  
No matches to eCLIP DataMATCHES To TargetScan▶ miR-324-5p:GCAUCCC

GGCCT

gg

ggtaaact  
Depth:3 (HOXB5OS)  
Ei-value:Undefined, Pi-value:Undefined  
Er-value:0.000, Pr-value:0.000  
No matches to eCLIP DataNo matches to TargetScan


taaact

taaact  
Depth:5 (HOXB_XENOPUS)  
Ei-value:Undefined, Pi-value:Undefined  
Er-value:0.000, Pr-value:0.000  
No matches to eCLIP DataNo matches to TargetScan

CGCACCTCTTAGGATCTTGCTCCCGGACTCATTCCCT

tccccac

tccccac  
Depth:2 (HOXB_DOG_ISOFORM1)  
Ei-value:Undefined, Pi-value:Undefined  
Er-value:0.000, Pr-value:0.000  
No matches to eCLIP DataMATCHES To TargetScan▶ miR-491-5p:GUGGGGA

CCCCTATTTTAAAG

tt

ttttatttgg  
Depth:2 (HOXB_DOG_ISOFORM1)  
Ei-value:Undefined, Pi-value:Undefined  
Er-value:0.000, Pr-value:0.000  
No matches to eCLIP DataNo matches to TargetScan

 476  


ttatttgg

ttttatttgg  
Depth:2 (HOXB_DOG_ISOFORM1)  
Ei-value:Undefined, Pi-value:Undefined  
Er-value:0.000, Pr-value:0.000  
No matches to eCLIP DataNo matches to TargetScan

GTCGTCTGTATC

aatttagaa

aatttagaa  
Depth:3 (HOXB5OS)  
Ei-value:Undefined, Pi-value:Undefined  
Er-value:0.000, Pr-value:0.000  
No matches to eCLIP DataNo matches to TargetScan

C

gagataaa

gagataaa  
Depth:2 (HOXB_DOG_ISOFORM1)  
Ei-value:Undefined, Pi-value:Undefined  
Er-value:0.000, Pr-value:0.000  
No matches to eCLIP DataNo matches to TargetScan

TTAAGACAAAGAAAGTAAAATAAATCGAAATAAAATATAGGAATAGCTCTTGGCGAAAA                        573
```

|  |  |  |  |  |  |  |
| --- | --- | --- | --- | --- | --- | --- |
| | | | | | | | | | | | | | |
| 2 |  |  | 5 |  |  | 8 |
| Depth of motif conservation (number of species) | | | | | | |

  
  

---

  

## >HOXB-AS3 TO HOXB5OS (573 bases)

```
gtcata

gtcata  
Depth:5 (HOXB_XENOPUS)  
Ei-value:Undefined, Pi-value:Undefined  
Er-value:0.000, Pr-value:0.000  
No matches to eCLIP DataNo matches to TargetScan


gcgacttt

gcgacttt  
Depth:5 (HOXB_XENOPUS)  
Ei-value:Undefined, Pi-value:Undefined  
Er-value:0.000, Pr-value:0.000  
No matches to eCLIP DataNo matches to TargetScan


tggg

gtcatagcgacttttggg  
Depth:4 (HOXB_OPOSSUM)  
Ei-value:Undefined, Pi-value:Undefined  
Er-value:0.000, Pr-value:0.000  
No matches to eCLIP DataNo matches to TargetScan

A

tagtttgct

tagtttgct  
Depth:4 (HOXB_OPOSSUM)  
Ei-value:Undefined, Pi-value:Undefined  
Er-value:0.000, Pr-value:0.000  
No matches to eCLIP DataNo matches to TargetScan

ATCGA

caaaggg

caaaggg  
Depth:4 (HOXB_OPOSSUM)  
Ei-value:Undefined, Pi-value:Undefined  
Er-value:0.000, Pr-value:0.000  
No matches to eCLIP DataNo matches to TargetScan

A

gacaaagtca

gacaaagtca  
Depth:3 (HOXB5OS)  
Ei-value:Undefined, Pi-value:Undefined  
Er-value:0.000, Pr-value:0.000  
No matches to eCLIP DataNo matches to TargetScan

AGGGGTGAAGGGA

aaggagg

aaggagg  
Depth:3 (HOXB5OS)  
Ei-value:Undefined, Pi-value:Undefined  
Er-value:0.000, Pr-value:0.000  
No matches to eCLIP DataNo matches to TargetScan

GCCAAGTAG

agcctc

agcctc  
Depth:3 (HOXB5OS)  
Ei-value:Undefined, Pi-value:Undefined  
Er-value:0.000, Pr-value:0.000  
No matches to eCLIP DataMATCHES To TargetScan▶ miR-485-5p:GAGGCUG

CACGACCCTCGGCTTCCT

cctcacca

cctcacca  
Depth:3 (HOXB5OS)  
Ei-value:Undefined, Pi-value:Undefined  
Er-value:0.000, Pr-value:0.000  
No matches to eCLIP DataNo matches to TargetScan


g

gctcccc  
Depth:3 (HOXB5OS)  
Ei-value:Undefined, Pi-value:Undefined  
Er-value:0.000, Pr-value:0.000  
No matches to eCLIP DataNo matches to TargetScan


ctcccc

ctcccc  
Depth:4 (HOXB_OPOSSUM)  
Ei-value:Undefined, Pi-value:Undefined  
Er-value:0.010, Pr-value:0.000  
No matches to eCLIP DataNo matches to TargetScan

C 120  
 TCCCTCCAAGTCCA

gtaagaagtt

gtaagaagtt  
Depth:4 (HOXB_OPOSSUM)  
Ei-value:Undefined, Pi-value:Undefined  
Er-value:0.000, Pr-value:0.000  
No matches to eCLIP DataNo matches to TargetScan


gggcc

gtaagaagttgggcc  
Depth:3 (HOXB5OS)  
Ei-value:Undefined, Pi-value:Undefined  
Er-value:0.000, Pr-value:0.000  
No matches to eCLIP DataNo matches to TargetScan

A

agctg

agctggaagggattgaccg  
Depth:3 (HOXB5OS)  
Ei-value:Undefined, Pi-value:Undefined  
Er-value:0.000, Pr-value:0.000  
No matches to eCLIP DataMATCHES To TargetScan▶ miR-188-5p:AUCCCUU▶ miR-204-5p/211-5p:UCCCUUU


gaaggga

gaaggga  
Depth:4 (HOXB_OPOSSUM)  
Ei-value:Undefined, Pi-value:Undefined  
Er-value:0.000, Pr-value:0.000  
No matches to eCLIP DataMATCHES To TargetScan▶ miR-204-5p/211-5p:UCCCUUU


ttgaccg

agctggaagggattgaccg  
Depth:3 (HOXB5OS)  
Ei-value:Undefined, Pi-value:Undefined  
Er-value:0.000, Pr-value:0.000  
No matches to eCLIP DataMATCHES To TargetScan▶ miR-188-5p:AUCCCUU▶ miR-204-5p/211-5p:UCCCUUU

G||CCGTTTCCTCTCCCTCGCC

ggcctc

ggcctc  
Depth:4 (HOXB_OPOSSUM)  
Ei-value:Undefined, Pi-value:Undefined  
Er-value:0.010, Pr-value:0.000  
No matches to eCLIP DataNo matches to TargetScan

G

gcggagat

gcggagattccaggccc  
Depth:3 (HOXB5OS)  
Ei-value:Undefined, Pi-value:Undefined  
Er-value:0.000, Pr-value:0.000  
No matches to eCLIP DataMATCHES To TargetScan▶ miR-216a-5p:AAUCUCA▶ miR-216b-5p:AAUCUCU


tccaggc

tccaggc  
Depth:4 (HOXB_OPOSSUM)  
Ei-value:Undefined, Pi-value:Undefined  
Er-value:0.000, Pr-value:0.000  
No matches to eCLIP DataNo matches to TargetScan


cc

gcggagattccaggccc  
Depth:3 (HOXB5OS)  
Ei-value:Undefined, Pi-value:Undefined  
Er-value:0.000, Pr-value:0.000  
No matches to eCLIP DataMATCHES To TargetScan▶ miR-216a-5p:AAUCUCA▶ miR-216b-5p:AAUCUCU

TATAGAAACCAGGACGTCCCTT

agc

agcgccaccgcc  
Depth:3 (HOXB5OS)  
Ei-value:Undefined, Pi-value:Undefined  
Er-value:0.000, Pr-value:0.000  
No matches to eCLIP DataNo matches to TargetScan

 238  


agcgccaccgcc  
Depth:3 (HOXB5OS)  
Ei-value:Undefined, Pi-value:Undefined  
Er-value:0.000, Pr-value:0.000  
No matches to eCLIP DataNo matches to TargetScan


gccacc

gccacc  
Depth:5 (HOXB_XENOPUS)  
Ei-value:Undefined, Pi-value:Undefined  
Er-value:0.000, Pr-value:0.000  
No matches to eCLIP DataNo matches to TargetScan


gcc

agcgccaccgcc  
Depth:3 (HOXB5OS)  
Ei-value:Undefined, Pi-value:Undefined  
Er-value:0.000, Pr-value:0.000  
No matches to eCLIP DataNo matches to TargetScan

TCACATGCCAGTGCTGCCGGGAACCCAGCGATATCCGCACCAG||CGGAGAAGGTTCCAGGCTGCCGGCGGCGGCGCAGAGAGCGGGAAGAGAGGCTCGGAGGAAGCCCCG 356  
 GGCGTGGCGTGGTCAGGCTCCGAGAGCG

gccggga

gccggga  
Depth:3 (HOXB5OS)  
Ei-value:Undefined, Pi-value:Undefined  
Er-value:0.000, Pr-value:0.000  
No matches to eCLIP DataNo matches to TargetScan

TGCGG

ccacac

ccacac  
Depth:3 (HOXB5OS)  
Ei-value:Undefined, Pi-value:Undefined  
Er-value:0.000, Pr-value:0.000  
No matches to eCLIP DataNo matches to TargetScan

CGGCCT

gg

ggtaaact  
Depth:3 (HOXB5OS)  
Ei-value:Undefined, Pi-value:Undefined  
Er-value:0.000, Pr-value:0.000  
No matches to eCLIP DataNo matches to TargetScan


taaact

taaact  
Depth:5 (HOXB_XENOPUS)  
Ei-value:Undefined, Pi-value:Undefined  
Er-value:0.000, Pr-value:0.000  
No matches to eCLIP DataNo matches to TargetScan

CGCACCTCTTAGGATCTTGCTCCCGGACTCATTCCCTTCCCCACCCCCTATTTTAAAGTT 476  
 TTATTTGGGTCGTCTGTATC

aatttagaa

aatttagaa  
Depth:3 (HOXB5OS)  
Ei-value:Undefined, Pi-value:Undefined  
Er-value:0.000, Pr-value:0.000  
No matches to eCLIP DataNo matches to TargetScan

CGAGATAAATTAAGACAAAGAAAGTAAAATAAATCGAAATAAAATATAGGAATAGCTCTTGGCGAAAA                        573
```

|  |  |  |  |  |  |  |
| --- | --- | --- | --- | --- | --- | --- |
| | | | | | | | | | | | | | |
| 2 |  |  | 5 |  |  | 8 |
| Depth of motif conservation (number of species) | | | | | | |

  
  

---

  

## >HOXB-AS3 TO HOXB\_OPOSSUM (573 bases)

```
gtcata

gtcata  
Depth:5 (HOXB_XENOPUS)  
Ei-value:Undefined, Pi-value:Undefined  
Er-value:0.000, Pr-value:0.000  
No matches to eCLIP DataNo matches to TargetScan


gcgacttt

gcgacttt  
Depth:5 (HOXB_XENOPUS)  
Ei-value:Undefined, Pi-value:Undefined  
Er-value:0.000, Pr-value:0.000  
No matches to eCLIP DataNo matches to TargetScan


tggg

gtcatagcgacttttggg  
Depth:4 (HOXB_OPOSSUM)  
Ei-value:Undefined, Pi-value:Undefined  
Er-value:0.000, Pr-value:0.000  
No matches to eCLIP DataNo matches to TargetScan

A

tagtttgct

tagtttgct  
Depth:4 (HOXB_OPOSSUM)  
Ei-value:Undefined, Pi-value:Undefined  
Er-value:0.000, Pr-value:0.000  
No matches to eCLIP DataNo matches to TargetScan

ATCGA

caaaggg

caaaggg  
Depth:4 (HOXB_OPOSSUM)  
Ei-value:Undefined, Pi-value:Undefined  
Er-value:0.000, Pr-value:0.000  
No matches to eCLIP DataNo matches to TargetScan

AGACAAAGTCAAGGGGTGAAGGGAAAGGAGGGCCAAGTAGAGCCTCCACGACCCTCGGCTTCCTCCTCACCAG

ctcccc

ctcccc  
Depth:4 (HOXB_OPOSSUM)  
Ei-value:Undefined, Pi-value:Undefined  
Er-value:0.010, Pr-value:0.000  
No matches to eCLIP DataNo matches to TargetScan

C 120  
 TCCCTCCAAGTCCA

gtaagaagtt

gtaagaagtt  
Depth:4 (HOXB_OPOSSUM)  
Ei-value:Undefined, Pi-value:Undefined  
Er-value:0.000, Pr-value:0.000  
No matches to eCLIP DataNo matches to TargetScan

GGGCCAAGCTG

gaaggga

gaaggga  
Depth:4 (HOXB_OPOSSUM)  
Ei-value:Undefined, Pi-value:Undefined  
Er-value:0.000, Pr-value:0.000  
No matches to eCLIP DataMATCHES To TargetScan▶ miR-204-5p/211-5p:UCCCUUU

TTGACCGG||CCGTTTCCTCTCCCTCGCC

ggcctc

ggcctc  
Depth:4 (HOXB_OPOSSUM)  
Ei-value:Undefined, Pi-value:Undefined  
Er-value:0.010, Pr-value:0.000  
No matches to eCLIP DataNo matches to TargetScan

GGCGGAGAT

tccaggc

tccaggc  
Depth:4 (HOXB_OPOSSUM)  
Ei-value:Undefined, Pi-value:Undefined  
Er-value:0.000, Pr-value:0.000  
No matches to eCLIP DataNo matches to TargetScan

CCTATAGAAACCAGGACGTCCCTTAGC 238  


gccacc

gccacc  
Depth:5 (HOXB_XENOPUS)  
Ei-value:Undefined, Pi-value:Undefined  
Er-value:0.000, Pr-value:0.000  
No matches to eCLIP DataNo matches to TargetScan

GCCTCACATGCCAGTGCTGCCGGGAACCCAGCGATATCCGCACCAG||CGGAGAAGGTTCCAGGCTGCCGGCGGCGGCGCAGAGAGCGGGAAGAGAGGCTCGGAGGAAGCCCCG 356  
 GGCGTGGCGTGGTCAGGCTCCGAGAGCGGCCGGGATGCGGCCACACCGGCCTGG

taaact

taaact  
Depth:5 (HOXB_XENOPUS)  
Ei-value:Undefined, Pi-value:Undefined  
Er-value:0.000, Pr-value:0.000  
No matches to eCLIP DataNo matches to TargetScan

CGCACCTCTTAGGATCTTGCTCCCGGACTCATTCCCTTCCCCACCCCCTATTTTAAAGTT 476  
 TTATTTGGGTCGTCTGTATCAATTTAGAACGAGATAAATTAAGACAAAGAAAGTAAAATAAATCGAAATAAAATATAGGAATAGCTCTTGGCGAAAA                        573
```

|  |  |  |  |  |  |  |
| --- | --- | --- | --- | --- | --- | --- |
| | | | | | | | | | | | | | |
| 2 |  |  | 5 |  |  | 8 |
| Depth of motif conservation (number of species) | | | | | | |

  
  

---

  

## >HOXB-AS3 TO HOXB\_XENOPUS (573 bases)

```
gtcata

gtcata  
Depth:5 (HOXB_XENOPUS)  
Ei-value:Undefined, Pi-value:Undefined  
Er-value:0.000, Pr-value:0.000  
No matches to eCLIP DataNo matches to TargetScan


gcgacttt

gcgacttt  
Depth:5 (HOXB_XENOPUS)  
Ei-value:Undefined, Pi-value:Undefined  
Er-value:0.000, Pr-value:0.000  
No matches to eCLIP DataNo matches to TargetScan

TGGGATAGTTTGCTATCGACAAAGGGAGACAAAGTCAAGGGGTGAAGGGAAAGGAGGGCCAAGTAGAGCCTCCACGACCCTCGGCTTCCTCCTCACCAGCTCCCCC 120  
 TCCCTCCAAGTCCAGTAAGAAGTTGGGCCAAGCTGGAAGGGATTGACCGG||CCGTTTCCTCTCCCTCGCCGGCCTCGGCGGAGATTCCAGGCCCTATAGAAACCAGGACGTCCCTTAGC 238  


gccacc

gccacc  
Depth:5 (HOXB_XENOPUS)  
Ei-value:Undefined, Pi-value:Undefined  
Er-value:0.000, Pr-value:0.000  
No matches to eCLIP DataNo matches to TargetScan

GCCTCACATGCCAGTGCTGCCGGGAACCCAGCGATATCCGCACCAG||CGGAGAAGGTTCCAGGCTGCCGGCGGCGGCGCAGAGAGCGGGAAGAGAGGCTCGGAGGAAGCCCCG 356  
 GGCGTGGCGTGGTCAGGCTCCGAGAGCGGCCGGGATGCGGCCACACCGGCCTGG

taaact

taaact  
Depth:5 (HOXB_XENOPUS)  
Ei-value:Undefined, Pi-value:Undefined  
Er-value:0.000, Pr-value:0.000  
No matches to eCLIP DataNo matches to TargetScan

CGCACCTCTTAGGATCTTGCTCCCGGACTCATTCCCTTCCCCACCCCCTATTTTAAAGTT 476  
 TTATTTGGGTCGTCTGTATCAATTTAGAACGAGATAAATTAAGACAAAGAAAGTAAAATAAATCGAAATAAAATATAGGAATAGCTCTTGGCGAAAA                        573
```

|  |  |  |  |  |  |  |
| --- | --- | --- | --- | --- | --- | --- |
| | | | | | | | | | | | | | |
| 2 |  |  | 5 |  |  | 8 |
| Depth of motif conservation (number of species) | | | | | | |

  
  

---

  

## >HOXB-AS3 TO HOXB\_COELACANTH\_HOXB (573 bases)

```
 NO CONSERVED NODES FOUND  
GTCATAGCGACTTTTGGGATAGTTTGCTATCGACAAAGGGAGACAAAGTCAAGGGGTGAAGGGAAAGGAGGGCCAAGTAGAGCCTCCACGACCCTCGGCTTCCTCCTCACCAGCTCCCCC 120  
 TCCCTCCAAGTCCAGTAAGAAGTTGGGCCAAGCTGGAAGGGATTGACCGG||CCGTTTCCTCTCCCTCGCCGGCCTCGGCGGAGATTCCAGGCCCTATAGAAACCAGGACGTCCCTTAGC 238  
 GCCACCGCCTCACATGCCAGTGCTGCCGGGAACCCAGCGATATCCGCACCAG||CGGAGAAGGTTCCAGGCTGCCGGCGGCGGCGCAGAGAGCGGGAAGAGAGGCTCGGAGGAAGCCCCG 356  
 GGCGTGGCGTGGTCAGGCTCCGAGAGCGGCCGGGATGCGGCCACACCGGCCTGGTAAACTCGCACCTCTTAGGATCTTGCTCCCGGACTCATTCCCTTCCCCACCCCCTATTTTAAAGTT 476  
 TTATTTGGGTCGTCTGTATCAATTTAGAACGAGATAAATTAAGACAAAGAAAGTAAAATAAATCGAAATAAAATATAGGAATAGCTCTTGGCGAAAA                        573
```

|  |  |  |  |  |  |  |
| --- | --- | --- | --- | --- | --- | --- |
| | | | | | | | | | | | | | |
| 2 |  |  | 5 |  |  | 8 |
| Depth of motif conservation (number of species) | | | | | | |

  
  

---

  

## >HOXB-AS3 TO HOXB\_GAR (573 bases)

```
 NO CONSERVED NODES FOUND  
GTCATAGCGACTTTTGGGATAGTTTGCTATCGACAAAGGGAGACAAAGTCAAGGGGTGAAGGGAAAGGAGGGCCAAGTAGAGCCTCCACGACCCTCGGCTTCCTCCTCACCAGCTCCCCC 120  
 TCCCTCCAAGTCCAGTAAGAAGTTGGGCCAAGCTGGAAGGGATTGACCGG||CCGTTTCCTCTCCCTCGCCGGCCTCGGCGGAGATTCCAGGCCCTATAGAAACCAGGACGTCCCTTAGC 238  
 GCCACCGCCTCACATGCCAGTGCTGCCGGGAACCCAGCGATATCCGCACCAG||CGGAGAAGGTTCCAGGCTGCCGGCGGCGGCGCAGAGAGCGGGAAGAGAGGCTCGGAGGAAGCCCCG 356  
 GGCGTGGCGTGGTCAGGCTCCGAGAGCGGCCGGGATGCGGCCACACCGGCCTGGTAAACTCGCACCTCTTAGGATCTTGCTCCCGGACTCATTCCCTTCCCCACCCCCTATTTTAAAGTT 476  
 TTATTTGGGTCGTCTGTATCAATTTAGAACGAGATAAATTAAGACAAAGAAAGTAAAATAAATCGAAATAAAATATAGGAATAGCTCTTGGCGAAAA                        573
```

|  |  |  |  |  |  |  |
| --- | --- | --- | --- | --- | --- | --- |
| | | | | | | | | | | | | | |
| 2 |  |  | 5 |  |  | 8 |
| Depth of motif conservation (number of species) | | | | | | |

  
  

---

  

## >HOXB-AS3 TO HOXB\_SHARK (573 bases)

```
 NO CONSERVED NODES FOUND  
GTCATAGCGACTTTTGGGATAGTTTGCTATCGACAAAGGGAGACAAAGTCAAGGGGTGAAGGGAAAGGAGGGCCAAGTAGAGCCTCCACGACCCTCGGCTTCCTCCTCACCAGCTCCCCC 120  
 TCCCTCCAAGTCCAGTAAGAAGTTGGGCCAAGCTGGAAGGGATTGACCGG||CCGTTTCCTCTCCCTCGCCGGCCTCGGCGGAGATTCCAGGCCCTATAGAAACCAGGACGTCCCTTAGC 238  
 GCCACCGCCTCACATGCCAGTGCTGCCGGGAACCCAGCGATATCCGCACCAG||CGGAGAAGGTTCCAGGCTGCCGGCGGCGGCGCAGAGAGCGGGAAGAGAGGCTCGGAGGAAGCCCCG 356  
 GGCGTGGCGTGGTCAGGCTCCGAGAGCGGCCGGGATGCGGCCACACCGGCCTGGTAAACTCGCACCTCTTAGGATCTTGCTCCCGGACTCATTCCCTTCCCCACCCCCTATTTTAAAGTT 476  
 TTATTTGGGTCGTCTGTATCAATTTAGAACGAGATAAATTAAGACAAAGAAAGTAAAATAAATCGAAATAAAATATAGGAATAGCTCTTGGCGAAAA                        573
```

|  |  |  |  |  |  |  |
| --- | --- | --- | --- | --- | --- | --- |
| | | | | | | | | | | | | | |
| 2 |  |  | 5 |  |  | 8 |
| Depth of motif conservation (number of species) | | | | | | |

  
  

---
