# Supplementary material for: Highly conserved and cis-acting lncRNAs produced from paralogous regions in the center of HOXA and HOXB clusters in the endoderm lineage
Source: PLoS Genet. 2021 Jul 19;17(7):e1009681. doi: 10.1371/journal.pgen.1009681 (PMC8330917; doi:10.1371/journal.pgen.1009681)
Supplement: Attachment — Submitted filename: HOX-AS PLoS Genetics point-by-point.pdf [file pgen.1009681.s016.pdf]

Point-by-point response to reviewers for *“Highly conserved and cis-acting lncRNAs produced from paralogous regions in the center of HOXA and HOXB clusters in the endoderm lineage”* by Degani et al.

Reviewer #1

The manuscript by Degani et al presents the analysis of the expression and regulation of two antisense lncRNAs within the Hox cluster. The study presented is of interest as the much remains to be learned regarding the function and impact of lncRNAs. The interest of the study stems from the high conservation of these two lncRNAs, their impact on Hox gene expression, but most importantly by the approach developed by the authors combining mining existing omic data and carefully designed experiments assessing the regulation of Hox genes by these lncRNAs and allowing to properly test transcription vs transcript mediated effect. The work is presented in a succinct, clear manner and is discussed in context of the current state of knowledge.

I have only minor comments

In the discussion it would be good to contrast the results shown on figure 2a and 2b with no or negative correlation between HOXA-AS3, HOXB-AS3 and the other HOX genes and the results stemming from the analyses of HT-29 cell as they appear somewhat conflicting. This is most likely the consequence of the tissues available and cell population represented in the data used for Figure 2.

We added a section on this to the Discussion (Page 26):

*“We report a positive effect of HOXA-AS3 and HOXB-AS3 production on the expression levels of their overlapping HOX5–7 genes. Notably, this positive effect does not translate into a tight co-expression between the lncRNAs and the protein-coding genes in this region when considering a broad range of conditions and cell types (**Fig. 2A** and **S4A Fig.**), likely because other mechanisms contribute to expression of the HOX5–7 genes in cells which do not express the lncRNAs. For example, we see strong expression of HOXA5 in the PFG cell population in differentiating hESCs (**Fig. 7A**).”*

- FigS1 - The labelling of strand and directionality for each of the species needs to be clarified. Perhaps more information in the legend, and a mention of the species-specificity of the strandedness where it is mentioned in the introduction. It is currently a little confusing as the figures seem to contradict the text without clarification that strandedness varies in the species presented.

We updated Figure S1 to always show the strand from which *HOXA-AS3* or *HOXB-AS3* are produced as the ‘+’ strand, to make it clear.

- Fig S1: can the authors check the tracks for the RNA-Seq and the annotations for the opossum, they are not aligned - spelling on the shark track - Kindey

We fixed both issues.

- Fig2 - the expression is of these lncRNAs is very low and the TPM>=1 threshold is understandable, but how robust are these findings to raising that threshold?

In the new Figure 2A that shows all the Hox genes, we now show the TPMs using several ranges, including 1–5, 5–50, and ≥50. The conclusions are not affected.

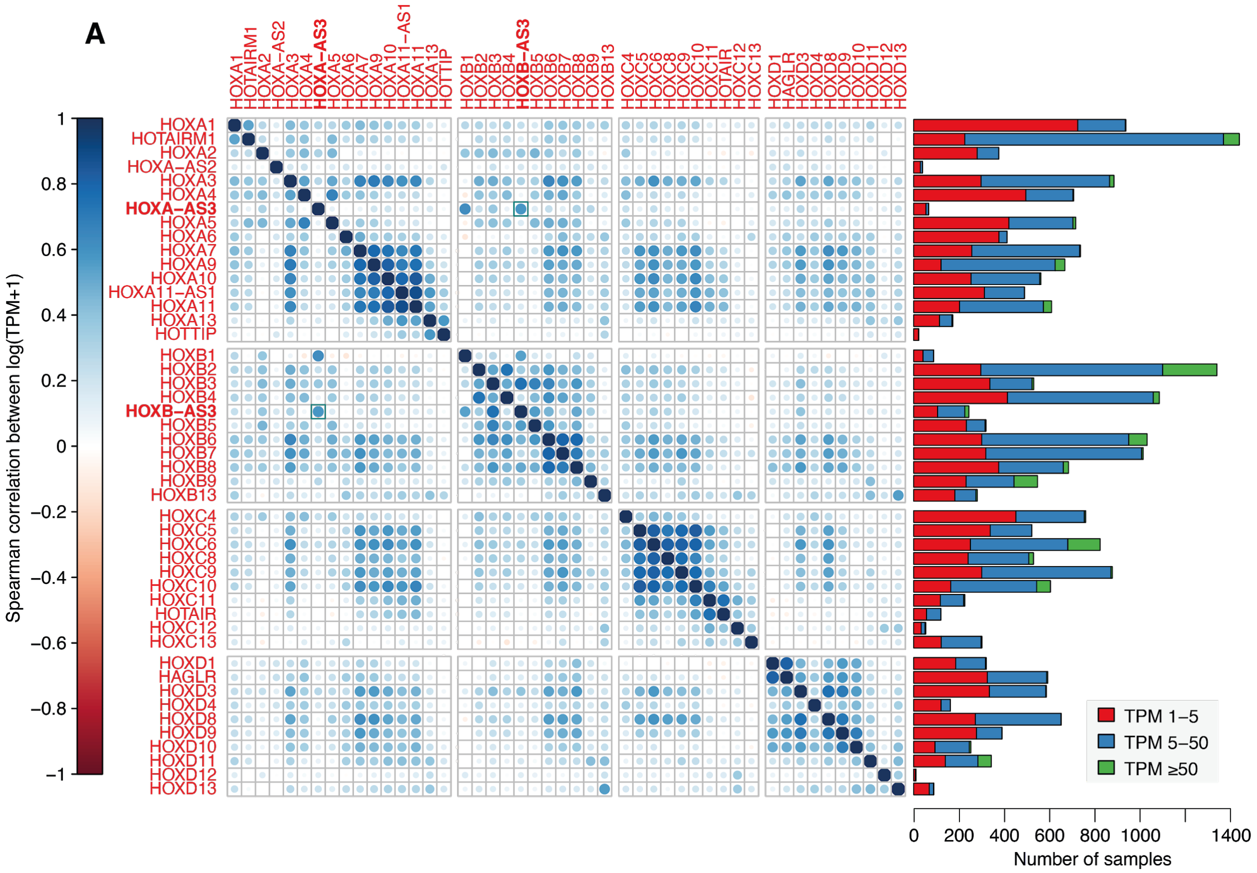

Figure 2A. Expression of HOXA-AS3 and HOXB-AS3 in adult and embryonic tissues. Left: Correlation coefficients between log-transformed FANTOM5.5 expression levels (Consortium, Fantom et al. 2014) in hundreds of samples for the indicated genes. Right: Number of samples in which each gene is expressed within the indicated TPM ranges.

- Fig S5 shows very large transcriptional activity on both strands, I would be careful in drawing conclusions here. To reduce the noise could you plot the splicing junctions? as the exons are clearly marked especially for HOXB-AS3?

Following the suggestion, we added the splice junction-supporting reads from the ENCODE data in the new Figure S6B. There is indeed substantial transcriptional activity on both strands, but the splice-supporting reads nevertheless support that the major isoforms expressed are the ones we work with.

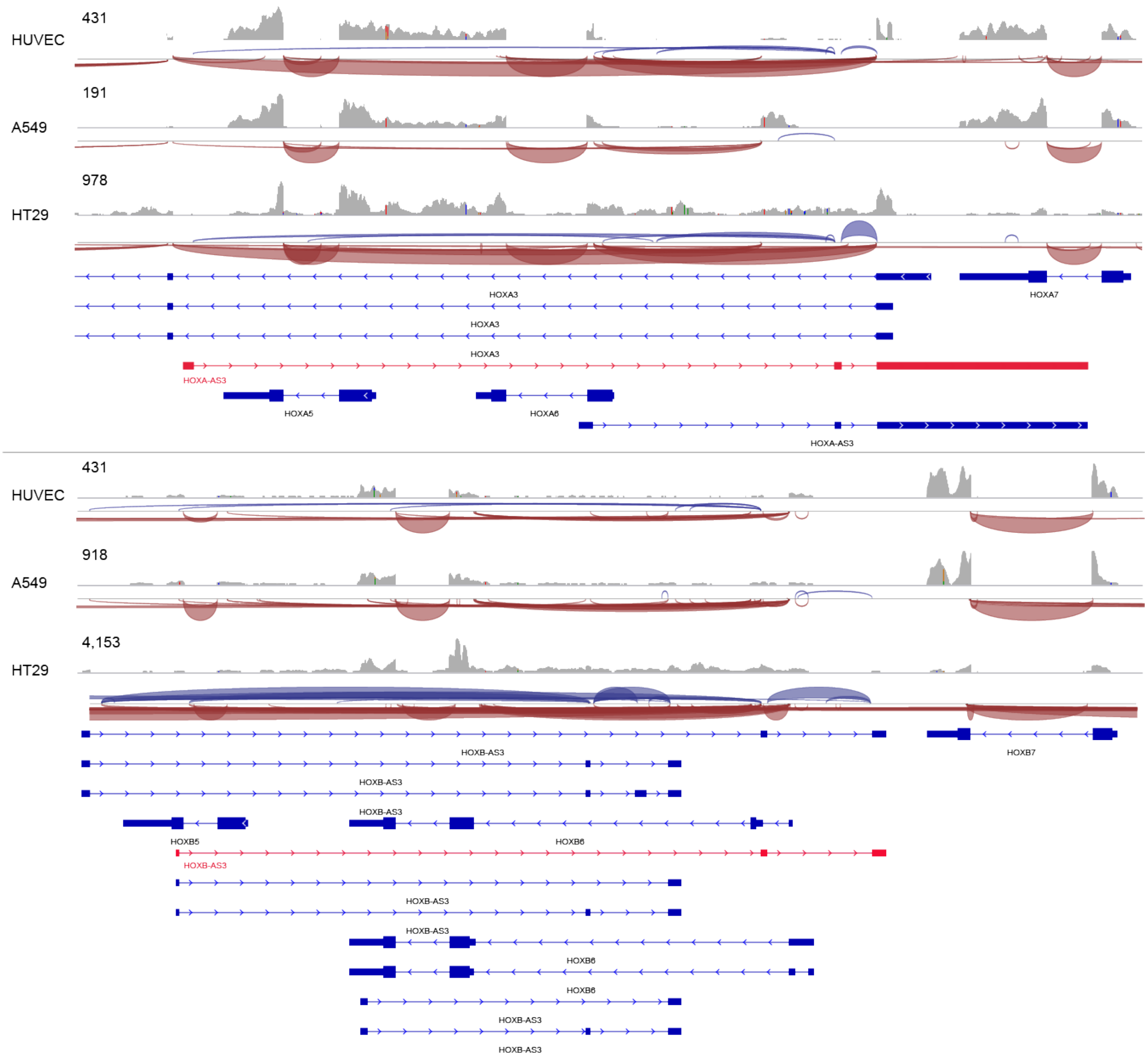

Figure S6B. Read coverage from the ENCODE datasets in IGV genome browser, showing the agglomerated coverage from both strands, and the splice-junction-supporting reads from the '+' strand (blue) and the '-' strand (red).

Page 9: "These results suggest that HOXA-AS3 and HOXB-AS3 production or their RNA products have a positive regulatory effect on the expression of the neighboring genes HOX5–7 genes." The authors do not show significant effect of activation for HOXB-AS3 it would be good to slightly change the phrasing to also include this observation.

We added a note on this in the preceding sentence: *"OE in HT-29 cells resulted in effects opposite to those observed following lncRNA KD, as it increased expression of the adjacent genes, significantly for HOXA-AS3* (***Fig. 3C-D***)*. "*

- Fig 6D - the arrows for HOXA-AS3, are these exhaustive? It looks like there are other molecules (including on the edge of next cell in the green "Phall region" top left of image). Do the different arrows colours represent anything? They are not referred to in the manuscript anywhere? Refer to them in the manuscript or remove them.

The arrows are not meant to be exhaustive, and we now clarify this in the figure legend. The different colors were a mistake that has now been fixed.

Reviewer #2

The authors of the study "Highly conserved and cis-acting lncRNAs produced from paralogous regions in the center of HOXA and HOXB clusters in the endoderm lineage" focus on a lncRNA derived antisense to HOXA3 in several species. Using their expertise in evolutionary conservation they start by finding syntenic regions across multiple species comprised of an array of evolutionary distances. The PhyloCSF analysis indicates that in most species this lncRNA is not likely to encode a protein (where as the positive control of HOX exons show clear synonymous mutations throughout evolution expected for proteins). Deeper sequence analysis reveals a tandem CDX1/2 sites that are very conserved. The authors perform co-expression analysis and find these two loci are highly correlated in expression. Moreover, it is expected that in IMR90 that only express proximal HOX genes that these genes would be expressed -- the authors find the same for HT-29 cancer line (where as a primary fibroblast line may have been more beneficial for normal HOX biology and a system developed by Howard Chang's lab (Rinn et al. PLoS Genetics 2006, Figure 7). The authors used the HT-29 line to perform LOF studies using CRISPR-I. They observe that the neighboring genes in both cases are down-regulated upon LOF of the lncRNA. Similarly, GOF results in increased expression of neighboring genes. The authors continue to show that HOXA3-as is expressed in specific regions of the intestine, but not relative to the HOXB-as. Moreover, the authors investigate the roles of these lncRNAs in hESC differentiation and find they are specifically expressed in mid/hind gut (MHG). The authors then perform CRISPR based LOF/GOF and see significant but very small effect sizes in regulation of MHG differentiation. Finally, the authors explore the transcriptional regulation of these Antisense lncRNAs by CDX1/2. They show that depletion of CDX1 down-regulates both HOXA3/B-as lncRNAs.

Overall, the evolutionary analysis of this study is interesting and the co-expression compelling. Yet all the LOF/GOF studies are far less convincing, mostly owing to highly variable and or small effect sizes. I have the following concerns before high-profile publication in PLoS Genetics.

1) How often does the CCATAA motif show up in randomly selected transcripts? Can the authors determine if this is unique to the HOXA/B-as genes relative to all other HOX genes?

We address this point by using JASPAR predictions of CDX1/2 binding site (that include CCATAA as well as other related motifs) and counting the number of hits in the ∓100 bp region around all human promoters. We find that the *HOXA-AS3* has 11 motif hits in its promoter, more than any other HOX genes, and more than 99.95% of human promoters annotated in FANOM5.5 (only 83 of the 200K promoters have 11 hits or more). *HOXB-AS3* promoter has two CDX1/2 motif hits, placing it in the first quartile of human promoters. We illustrate this in a new **S3B Fig.** and mention this in the text (Page 5):

*"When considering all the human promoters annotated in FANTOM5.5 (Consortium, Fantom et al. 2014), the HOXA-AS3 promoter contained 11 predicted CDX binding sites, a number of predicted binding sites larger than that in 99.95% of human promoters annotated in FANOM5.5 (only 83 of the 200K promoters had 11 predicted sites or more). HOXB-AS3 promoter had two predicted sites, a number comparable to that of several other Hox genes (**S3B Fig.**)."*

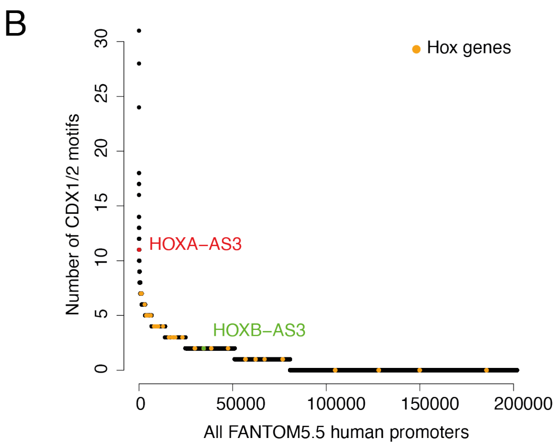

Figure S3B. Number of CDX1 or CDX2 binding sites predicted by JASPAR (Khan et al. 2018) in 201,802 human promoters annotated in FANTOM5.5 (Consortium, Fantom et al. 2014). For each TSS we considered the region −100..+100 relative to the TSS. Selected genes are highlighted.

2) HOXA/B-as lncRNAs are highly correlated. If all hox pairs are considered is the correlation more than expected for other hox genes that are highly correlated?

We have revised this analysis to include all Hox protein-coding genes and the prominent lncRNAs in the cluster, showing the human correlations in **Fig. 2A** and the mouse in **S4A Fig.**. The analysis of human data is generally more informative, as it includes more samples where *HOXA-AS3* is expressed with TPM of at least 1 (65 compared to 17 for the mouse *Hoxxas3*). From this analysis the correlation between *HOXA-AS3* and *HOXB-AS3* is comparable to the cross-correlation between other protein-coding genes in the HOX clusters, in particular the genes expressed in posterior regions. We describe this new analysis in the text (Page 4):

*"The correlation between HOXA-AS3 and HOXB-AS3 was comparable to the correlations between Hox protein-coding genes with a posterior expression domain (**Fig. 2A**), and larger than that found typically between Hox genes in different clusters (**S4A Fig.**). In mouse, Hoxaas3 and Hoxb5os were also more tissue specific than the Hox genes they overlapped, but the correlation between them was weaker (**S4B Fig.**). Notably, correlations in mouse were more difficult to assess, as Hoxaas3 was expressed with TPM≥1 only in 17 samples (for comparison, HOXA-AS3 was expressed with TPM≥1 in 65 human samples)."*

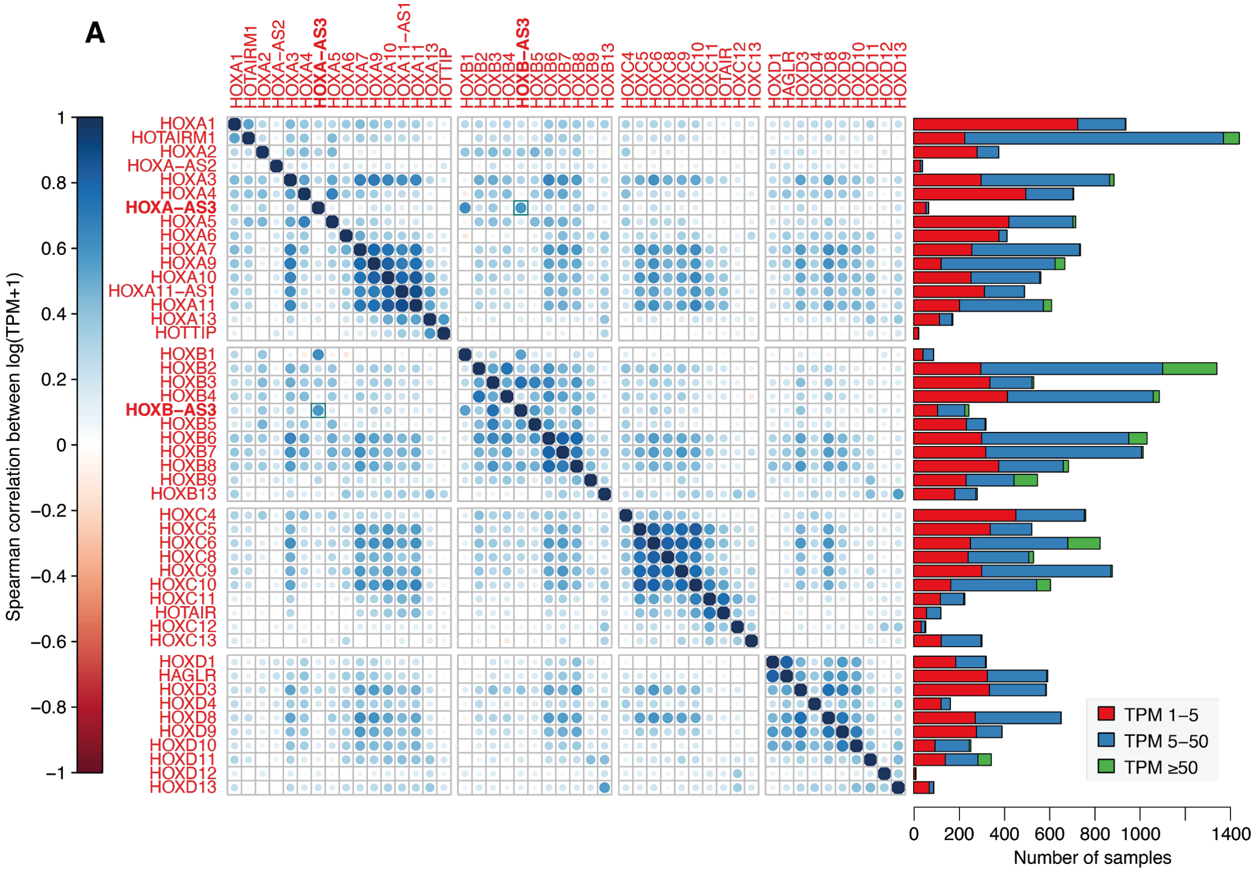

Figure 2A. Expression of HOXA-AS3 and HOXB-AS3 in adult and embryonic tissues. Left: Correlation coefficients between log-transformed FANTOM5.5 expression levels (Consortium, Fantom et al. 2014) in hundreds of samples for the indicated genes. Right: Number of samples in which each gene is expressed within the indicated TPM ranges.

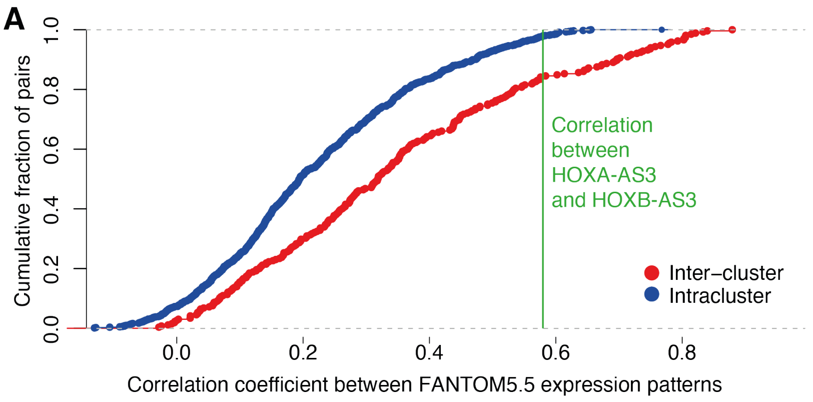

Figure S4A. Distribution of correlation coefficients between expression patterns of pairs of genes within the same Hox cluster (red) and found in different Hox clusters (blue). The correlation coefficient between *HOXA-AS3* and *HOXB-AS3* is shown in green.

For example HoxA1-7 should be highly correlated with HOXD1-7. Essentially one would expect a high correlation between the binary proximal and distal information expressed in each hox cluster. This was found by the Duboule lab in vivo as well. Counter to the co-linearity model it is noted in this and other studies that the hox cluster either expresses HOX1-7 or HOX9-13 paralogs (at least for HOXA and D clusters). Thus, it would not be surprising to find strong correlation across HOX clusters since there are typically only binary patters on prox-on or distal-on but not prox & distal on (as would have been suggested by the collinearity model).

As mentioned above, we see a strong correlation between the distal genes, and less so for the proximal ones. We note that the genes overlapping *HOXA-AS3* and *HOXB-AS3*, HOXA5-6 and HOXB5-6 don't show a strong correlation with genes in other clusters.

Similarly, Is HOTAIRM1 as or more correlated with HOXA1 & HOXA3 as HOXB3-as and HOXA3-as pairs? Or how does the HOXA3/B3-as correlation compare to intra-cluster correlation? This could be done systematically with all HOX pairs and determine the observed value of HOXA3/B3 relative to all other correlations -- is the observed significant relative the expected empirical null?

We now show all the pairwise correlations in **Fig. 2** and **Fig. S4B** presented above, and plot all the pairwise correlation coefficients between genes in the same cluster or in different clusters in **Fig. S4A**. We find that the correlation between *HOXA-AS3* and *HOXB-AS3* is higher than most other correlations between the clusters and even within the cluster. This is mentioned in the text (Page 4):

*"The correlation between HOXA-AS3 and HOXB-AS3 was comparable to the correlations between Hox protein-coding genes with a posterior expression domain (**Fig. 2A**), and larger than that found typically between Hox genes in different clusters (**S4A Fig.**)"*

3) The authors show that LOF and GOF decrease and increase the neighboring genes respectively. I may have missed it, but did the authors check if there was cross talk between the HOXA/B LOF/GOF experiments? For example does LOF/GOF of HOXA3-as affect HOXB? Vice-versa? From my reading, when HOXA3-as CRISPR-I/A was performed the authors only looked at the neighboring genes in cis -- where as it would seem important to see if HOXA3-as also affected HOXB-as. Same for Figure 7.

To address this point we have now performed RNA-seq on HT-29 cells treated with siRNAs targetings *HOXA-AS3* and *HOXB-AS3*, and performed qRT-PCR on genes from the other clusters in HT-29 CRISPRi cells, and in hESC-derived MHG cells treated with CRISPRi for HOXB-AS3 or shRNAs for HOXA-AS3. We focused the RNA-seq on the siRNA experiments, as they target the RNA product, and are thus the best readout of the RNA product activity. As these new data show, KD of *HOXA-AS3* has an overall negative effect on expression of the HOXB genes, and KD of *HOXB-AS3* has a negative effect on expression of HOXA genes. For *HOXB-AS3* (which is generally more highly expressed in HT-29 cells, and so its KD consequences are easier to interpret by RNA-seq), it is also evident that the impact on the HOXB5–7 genes that it overlaps is stronger than the effect on HOXB or HOXA genes. We also discuss and analyze the functional signatures in the non-HOX genes affected by HOXA-AS3 and HOXB-AS3 KD.

"In order to characterize more broadly the consequences of down-regulation of HOXA-AS3 and HOXB-AS3, we used RNA-seq to profile transcriptome-wide gene expression in HT-29 cells treated with siRNAs targeting these lncRNAs or with a non-targeting control. RNAi resulted in

*reduction in expression of the lncRNAs, concomitantly with reduction in the overlapping genes, and a broad mild reduction in expression of genes in the HOXA and HOXB clusters (HOXC and HOXD clusters are mostly silent in HT-29 cells) (**Fig. 4D** and **S2 Table**). In the case of HOXB-AS3 it was apparent that the KD had a strong effect on the levels of the overlapping HOXB5–7 genes relative to the other HOX genes. The repressive effect of KD of HOXA-AS3 on HOXB genes, and of HOXB-AS3 KD on HOXA genes was validated by qRT-PCR following siRNA KD or CRISPRi of these genes (**S7A-B Fig.**) These results suggest that loss of HOXA-AS3 and HOXB-AS3 has broad effects on expression of genes from HOXA and HOXB clusters.*

*Beyond the effect on the expression of HOX genes, HOXB-AS3 had a larger effect on gene expression (**S7C Fig.**), consistently with its higher expression levels in HT-29 cells. Analysis of the gene expression changes using GOrilla [35] (**S2 Table**) showed that HOXA-AS3 KD was associated with a significant reduction in genes related to cell cycle and proliferation (top down-regulated GO category "mitotic cell cycle process" adjusted $P=1.36\times10^{-13}$), consistent with its reported positive effect of proliferation reported in other cell lines [36] [30] [37] (see Discussion). HOXB-AS3 led to a significant up-regulation of genes whose protein products are involved in ncRNA processing, and specifically in rRNA processing (adjusted $P=3.58\times10^{-5}$), potentially related to its reported functions in rRNA biogenesis observed in leukemia cells [38]. The changes in gene expression outside of the HOX clusters following HOXA-AS3 or HOXB-AS3 KD could result from the consequences of changes in gene expression or from additional trans-acting functions of these lncRNAs (see Discussion)."*

4) Minor: It is somewhat concerning that HT-29 cell lines have low expression and extra copies of HOX clusters -- where as IMIR90 would not have this issue.

We agree with the reviewer that the use of cancer cell lines is not optimal due to their karyotype, but *HOXA-AS3* is barely detectable in IMR-90 cells even in the very deeply sequenced ENCODE data, so they were not fit for our purposes. IMR-90 cells do express *HOXB-AS3*, as mentioned in the manuscript.

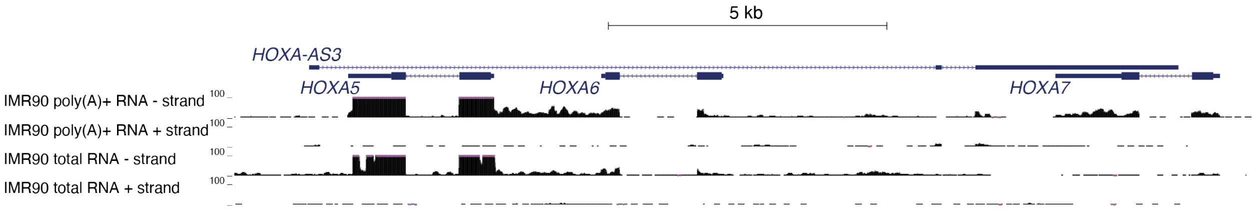

Reviewer Figure 1. RNA-seq coverage in the HOXA-AS3 locus in ENCODE project IMR-90 RNA-seq data (polyA-selected and total RNA). Values above 100 are shown as 100.

5) RNA-FISH wouldn't one expect to see more cytoplasmic localization of HOXA5 as a positive control for spatial resolution of RNA-FISH?

We now provide new images of smFISH images of *Hoxaas3* and *Hoxa5* in the mouse intestine, that show *Hoxa5* in both the nucleus and the cytoplasm. In ENCODE fractionation RNA-seq data, *HOXA5* is found in comparable amounts in the nucleus and cytoplasm, possibly because of low stability of the mRNA in the cytoplasm:

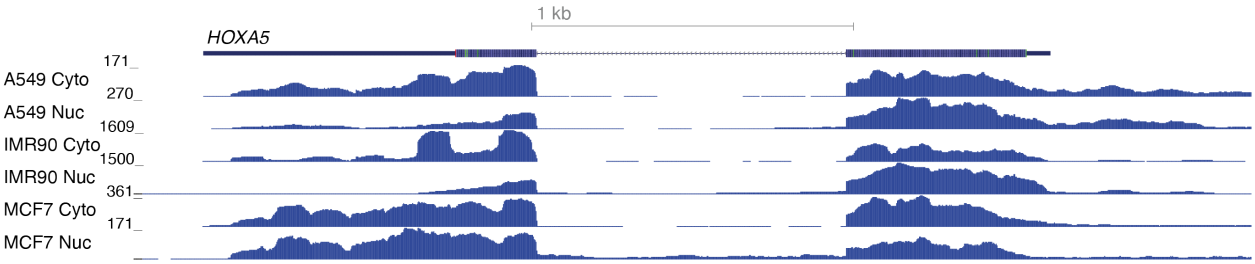

Reviewer Figure 2. RNA-seq coverage in the HOXA5 locus in ENCODE project RNA-seq data from the indicated cell lines (polyA-selected RNA, 1st replicate, only minus-strand data are shown).

6) Intestine FISH would be good to see HOXA3-as and HOXB-as co-localized with target genes as in Figure 5?

Unfortunately, as mentioned in the text, HOXB-AS3 is too short to be efficiently detected by Stellaris smFISH (*Hoxb5os* in mouse is only 597 nt, allowing only 11 probes even with the minimal filtering, whereas 25 probes are the minimum recommended by Stellaris). We now obtained and added new smFISH images showing co-localization of *Hoxaas3* and *Hoxa5* in the mouse intestine, matching what we observe in HT-29 cells. We now indicate this in the text (Page 21):

"*Hoxaas3* and *Hoxa5* were occasionally co-localized, similar to the observations in HT-29 cells (**Fig. 6D**)."

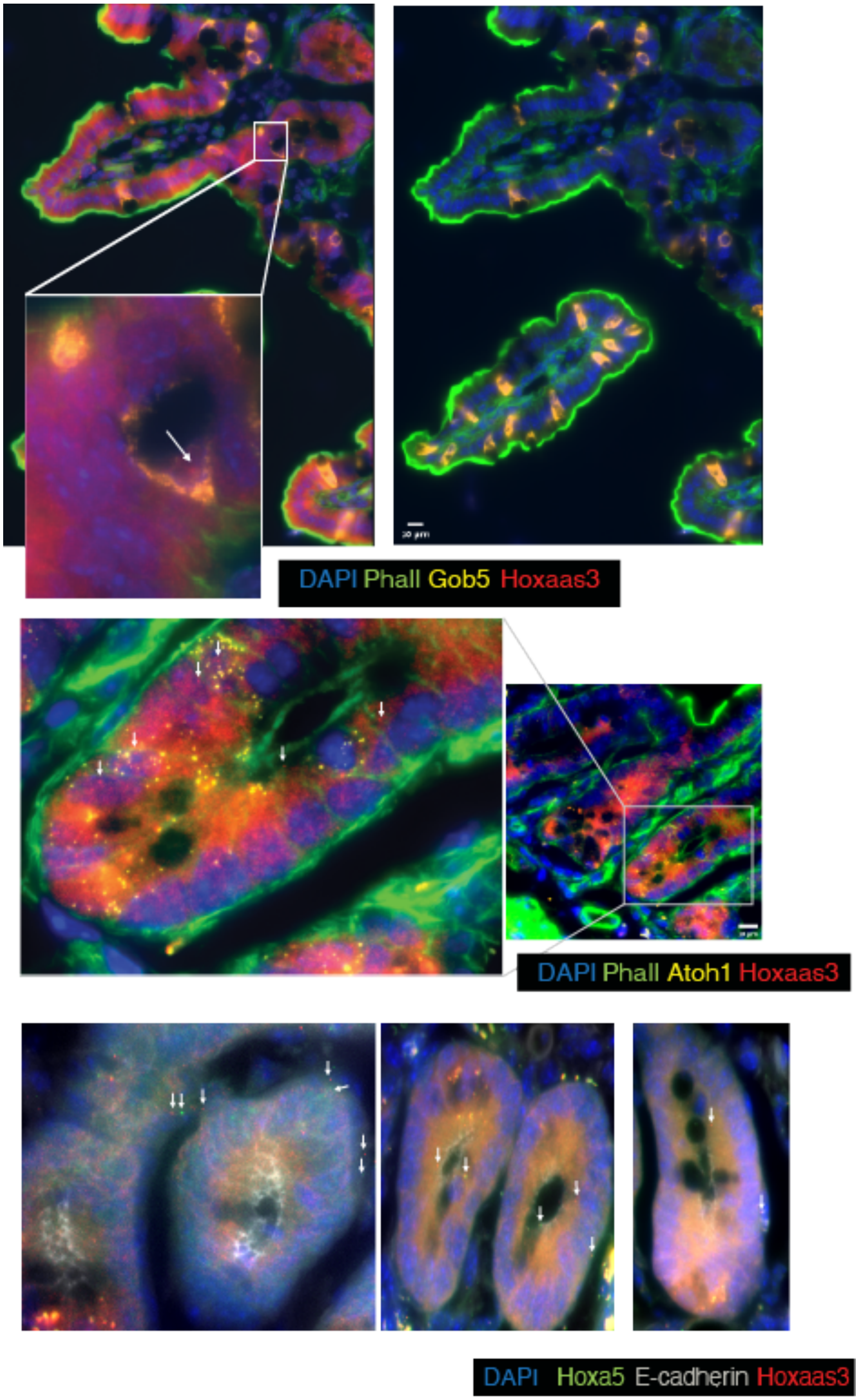

Figure 6D smFISH of *Hoxaas3*, *Hoxa5*, *Gob5* and *Atoh1* expression in the mouse intestine. Scale bar:10μm. Arrows indicate a subset of RNA molecules detected in the images.

7) Figure 7: everything in this figure trends the right way, but differences that are significant are very small in effect-size. While the HOXA3-as LOF/GOF seems ok in HESCs the depletion in MHG shows ~40% depletion of HOXA3-as and 20-30%% depletion of HOXA5. Whilst the non-significant OCT4 and SOX17 markers have a variance that encompasses the effect size of the "significant" changes. Does this have physiologically visible phenotype? For example, can the authors quantify using FISH approaches in previous figures that these markers and or genes are making a significant change in differentiation? Again is there cross-talk as the qRT-PCR only focuses on neighboring genes of LOF/GOF target and not the reciprocal cluster?

We agree with the reviewer that there is a substantial variability in the hESC differentiation, which is a complex system. We believe that the fact that we present multiple perturbation approaches (CRISPRi, CRISPRa, and shRNAs) in hESCs, and that the data support the findings from all these perturbation methods and also from siRNAs in the less noisy HT-29 system, add confidence to the results. We therefore would like to argue that the phenotypic characterization of the effects of *HOXA-AS3* and *HOXB-AS3* on hESC differentiation is out of the scope of the current study. To address the last point – we now added in **Figure 8E** qRT-PCR quantification of HOXA genes following CRISPRi of *HOXB-AS3* in MHG cells, and of HOXB genes in shHOXA-AS3 MHG cells. We observe reciprocal repression of the two clusters, similarly to what we now report in HT-29 cells. We note that in the context of hESC differentiation, it is difficult to dissect the direct consequences of *HOXA-AS3* and *HOXB-AS3* activity from the possible indirect effects, due to complexity of the differentiation process.

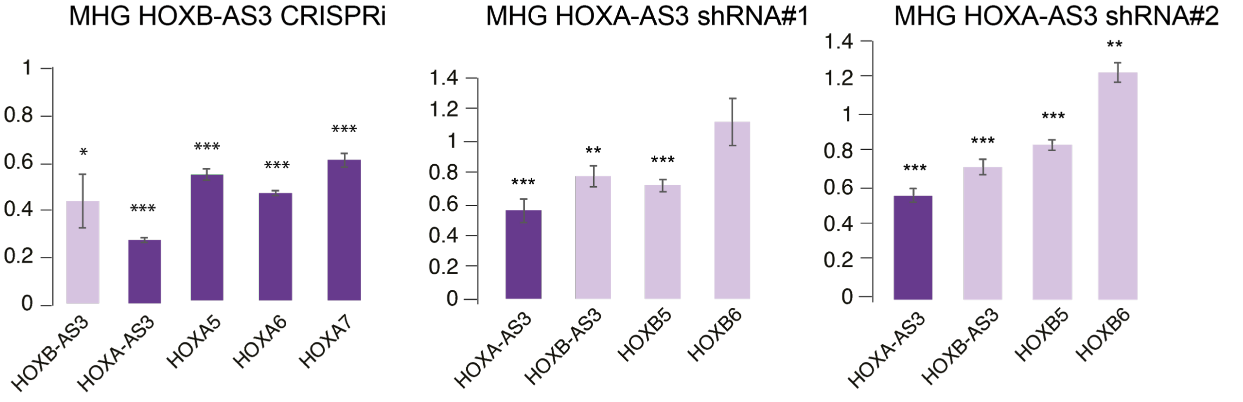

Figure S8F As in Fig. 7E (left) and Fig. 7F (right) for the indicated genes.

8) Surprising that the combo of CDX 1/2 has less of an affect than either alone?

Following the suggestion of reviewer #3, we removed the CDX1/2 data from the manuscript.

Reviewer #3

Hox cluster regulation has long been a central setting with which to reveal and dissect mechanisms of gene/genome regulation. Assessment of lncRNAs within Hox clusters have not been without significant controversy (Hotair), and the continued identification and functional assessment of Hox-embedded ncRNas is important to understand their quantitative impact in shaping Hox output and in revealing commonalities/differences in the mechanisms by which they control Hox cluster expression.

In this manuscript by Degani and colleagues demonstrate i) genomic conservation of two Hox-embedded lncRNAs across vertebrate species; ii) utilise published resources to good effect to characterise the developmental and adult expression of these lncRNAs with additional fluorescent in situ hybridisation to provide spatial detail; iii) perform in vitro functional analyses indicating these lncRNAs have a positive effect on Hox gene expression of the opposite strand. Overall, the manuscript is quite straightforward, well written, I have no major technical concerns with minor technical comments below. Greater understanding of global Hox impact, and assessment of any altered ESC differentiation endpoint, dowstream of altered Hox signatures, would strengthen the impact of this work.

Genomic and transcriptomic analyses:

The transcript assessment in Figure 1 looks simplified. For example, we know the Hoxa5/a6 locus is highly complex, with various transcripts produced (see work of Lucie Jeanotte). I understand this figure does not need to reflect that level of complexity in Hox protein coding genes, but is there any evidence of alternate antisense transcripts produced? What do the opening words “in human” mean – ie, what cumulative datasets is this data derived from? (I later see in Supp fig 2 there are 2 variants? … and Supp fig 5 that Hoxb-AS3 does appear 3 transcripts in cell lines). Please clarify.

Both loci indeed produce a high diversity of transcripts, and we focus most the analysis on the splice isoforms that are most common overall, as well as in the cells with work with. In the FANTOM and RNA-seq analysis we do combine all the annotated variants. We added a new **Figure S1A** that shows all the isoforms currently annotated in GENCODE alongside the FANTOM5.5 CAGE data, showing that the main promoters on the antisense strand in the center of the HOXA/HOXB clusters belong to the *HOXA-AS3* and *HOXB-AS3* isoforms that we focus on. In the new **Figure 6B** shown below we also now show all the splice-supporting reads from the ENCODE cell lines. We now clarified this in the text:

*“The central regions of HOX clusters give rise to a large variety of transcription products that undergo extensive alternative splicing (**S1A Fig.**). We first focused on HOXA-AS3, the main transcription start site of which starts ~700 nt downstream of the annotated 3' end of HOXA5 and it is transcribed antisense to HOXA5 and HOXA6, terminating in the single intron of HOXA7 (**Fig. 1A** and **S1A Fig.**).”*

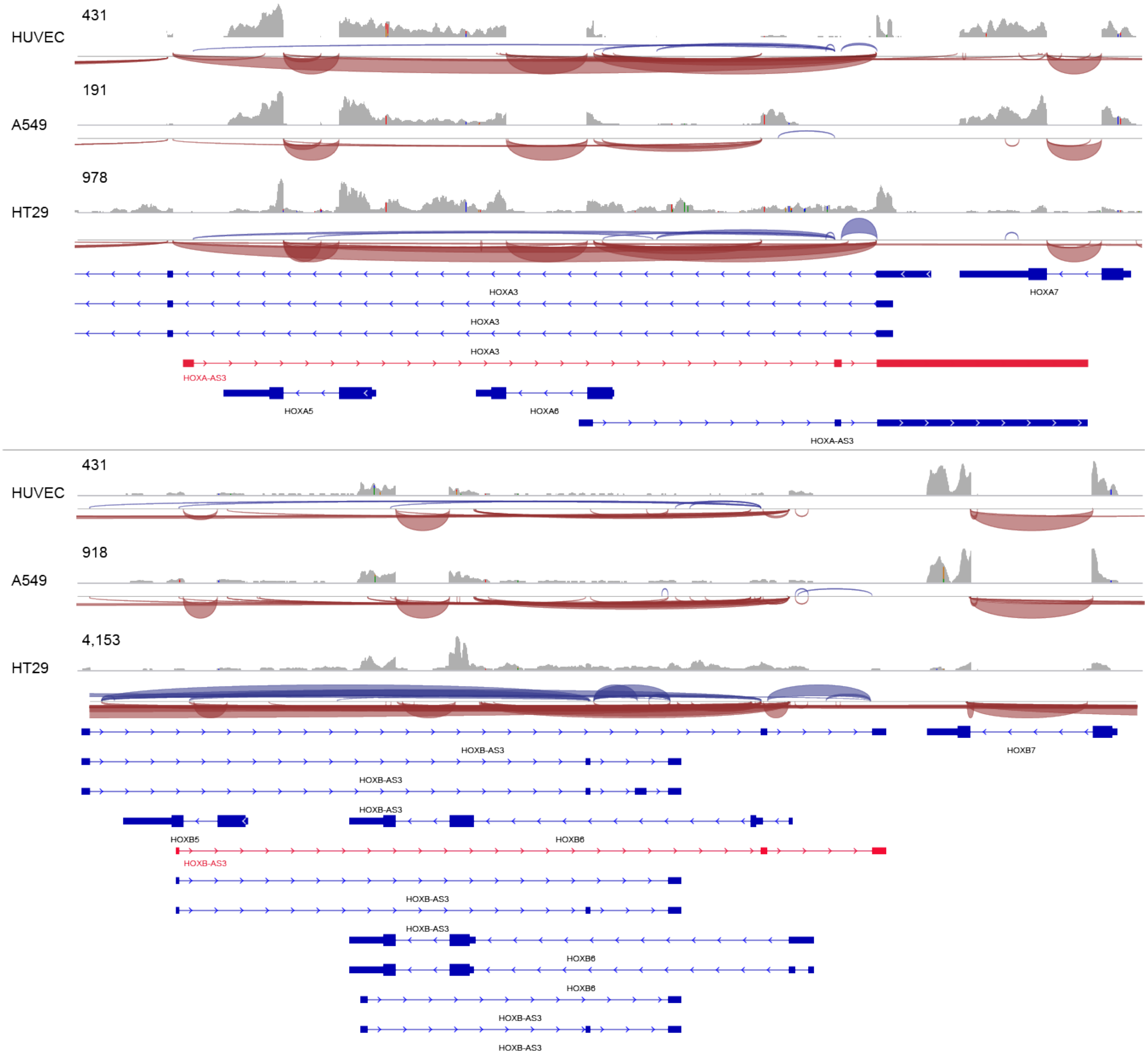

Figure S6B. Read coverage from the ENCODE datasets in IGV genome browser, showing the agglomerated coverage from both strands, and the splice-junction-supporting reads from the '+' strand (blue) and the '-' strand (red).

I believe the authors mean orthologue rather than homolog throughout when comparing species?

We indeed mean orthologs, and the text was updated accordingly.

Pg 4:

"The corresponding positions of the two lncRNAs and their high conservation in other species made us scrutinize…" Just to be clear, please add… the high conservation of their presence in other species (as written it could lead reader to think this was sequence conservation which I don't believe was assessed directly).

We changed the sequence accordingly (Page 5):

"The corresponding positions of the two lncRNAs and the high conservation of their presence in other species made us scrutinize and compare the sequences of their promoters."

Regarding this last point, is there any evidence of sequence conservation?

We now performed and described the sequence conservation of *HOXA-AS3* and *HOXB-AS3* using BLAST, and LncLOOM a tool we recently developed for studying subtle conservation signals in lncRNAs. These analyses are now described in the text (Page 3):

"HOXA-AS3 exhibited significant sequence similarity with the orthologs from mouse, opossum, and X. tropicalis (BLAST E-value<10^{-40}). Notably, homology with the X. tropicalis ortholog was restricted to the region overlapping HOXA7."

"HOXB-AS3 exhibited significant sequence similarity with the orthologs from mouse and opossum (BLAST E-value<10^{-40}), but not with more distant species. Comparison of the sequences with LncLOOM (Ross et al. 2021) *identified four motifs conserved in mammals and in X. tropicalis but no deeper conservation was detected (**Dataset S1**)."*

The single cell analysis is a good addition. The relatively higher expression of Hoxaas3 in NMP, caudal epiblast, caudal mesoderm would suggest these antisense transcripts are expressed similar to Hox protein coding genes in the overall whole embryo A-P context. Whole mount ISH of each antisense transcript would be a good addition here, particularly as this data indicates the antisense transcripts are more highly expressed than the protein-coding genes they overlap.

We address this point by re-analyzing the Geo-seq data set from E7.5 embryos (which allowed us to analyze *Hoxb5os* that wasn't part of the quantification in that study), and visualized the expression patterns of the Hox protein-coding genes and *Hoxaas3* and *Hoxb5os* side-by-side. These data are shown in **Fig. S5**, and discussed in the text (Page 9):

*"In order to assess the spatial expression patterns of Hoxaas3 and Hoxb5os and other Hox genes, we reanalyzed Geo-seq data from the mouse E7.5 embryos (Peng et al. 2019) (**S5 Fig.**). Both lncRNAs exhibited specific and overlapping expression domains in the region corresponding to primitive streak or 'late mesoderm' (sections 9-10, posterior region), consistently with the scRNA-seq data. Notably it has been suggested that some of the cells in this region are endoderm cells that egress through the mesoderm late in gastrulation (Peng et al. 2019; Nowotschin et al. 2019; Kwon, Viotti, and Hadjantonakis 2008; Chan et al. 2019). Hoxaas3 and Hoxb5os expression domain was more specific than that of the overlapping Hox genes, and interestingly, overlapped with the expression of Cdx1, and to a lesser extent Cdx2."*

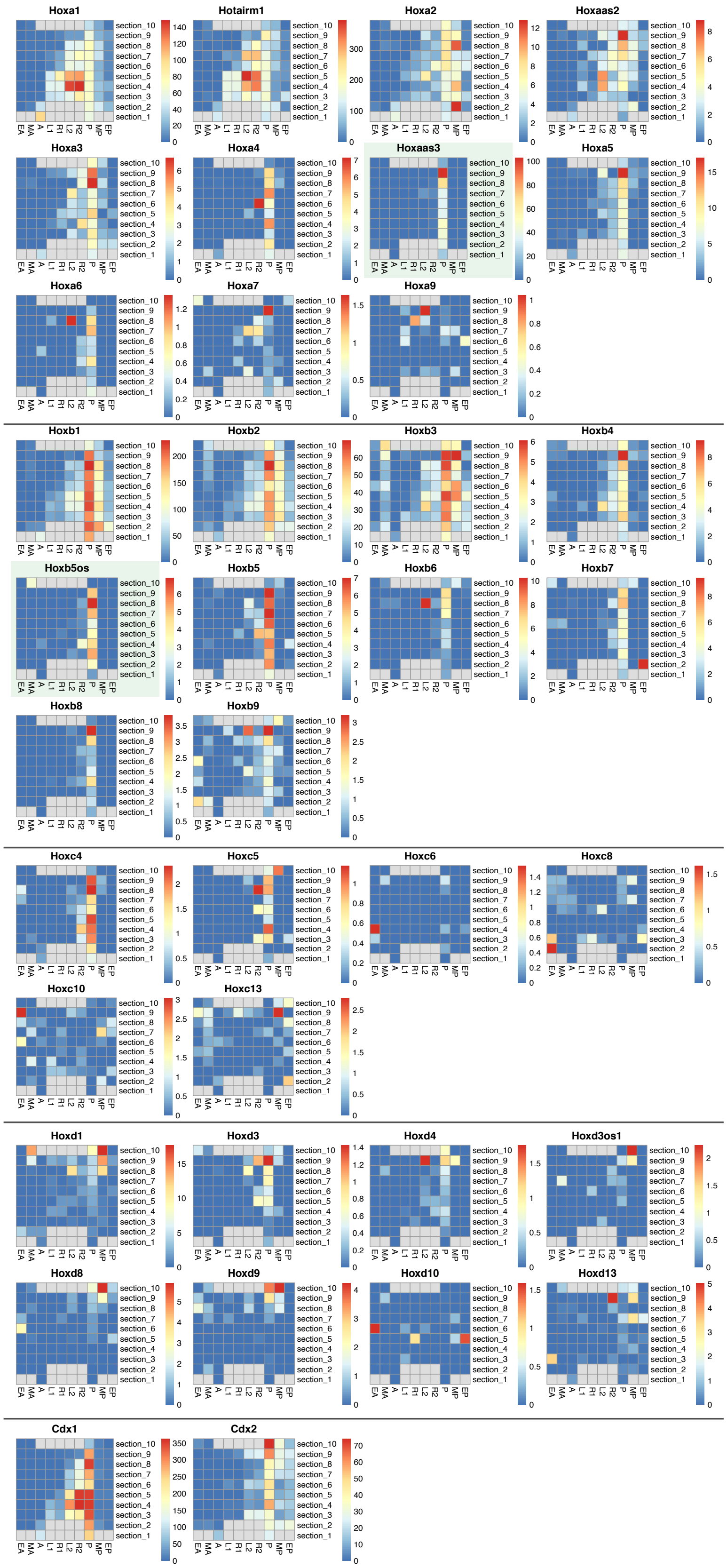

Figure S5. Spatial expression patterns of *HOXA-AS3* and *HOXB-AS3* and other genes in the E7.5 mouse embryo. Geo-seq data were re-mapped to the RefSeq annotations and visualized as in (Peng et al. 2019). Each gene is shown on a separate scale. Genes are grouped based on their genomic location or gene family.

Functional and expression analyses:

Elegant strategies employed, in some cases multiple strategies to corroborate.

For each gain or loss-of-function in vitro perturbation, a clear effect was observed for the Hox genes overlapping the antisense transcript in question (a5/a6/a7 for Hoxasa3). This manuscript would greatly benefit from a more comprehensive assessment of Hox genes both cis and trans, particularly given later the authors show it is the RNA transcript itself that is functional, not simply a local chromatin mechanism.

Following the requests of Reviewer #2 and #3, we now performed RNA-seq on cells treated with siRNAs targeting *HOXA-AS3* or *HOXB-AS3*, and performed qRT-PCR on genes from the other clusters in HT-29 CRISPRi cells, and in hESC-derived MHG cells treated with CRISPRi for HOXB-AS3 or shRNAs for HOXA-AS3. We focused the RNA-seq on the siRNA experiments, as they target the RNA product, and are thus the best readout of the RNA product activity. As these new data show, KD of *HOXA-AS3* has an overall negative effect on expression of the HOXB genes, and KD of *HOXB-AS3* has a negative effect on expression of HOXA genes. For *HOXB-AS3* (which is generally more highly expressed in HT-29 cells, and so its KD consequences are easier to interpret by RNA-seq), it is also evident that the impact on the HOXB5–7 genes that it overlaps is stronger than the effect on HOXB or HOXA genes. We also discuss and analyze the functional signatures in the non-HOX genes affected by *HOXA-AS3* and *HOXB-AS3* KD.

We now mention this in the text (Page 18)

Figure S6: MCF7 cell line, lncRNA activation – this experiment was done twice, with no indication of whether the Hox protein coding activation is significant (for a6 and a7 at least). Please repeat with stats or remove from manuscript.

We removed this experiment from the manuscript, as it indeed didn't contribute substantially.

Regarding Figure 6, I find it quite difficult to interpret image 6D. There appears to be a haze of red signal indicating Hoxaas3, however it is indicated in the text and from scRNAseq data to be restricted to certain cell types – this is not apparent. Can the authors please clarify, and describe what technical controls are performed.

In order to improve this figure, we have performed additional imaging and added additional panels showing cells expressing *Hoxaas3* and/or *Hoxa5*. We agree that it is difficult to see co-localization of *Hoxaas3* and the markers in individual cells, in part because of the large differences in expression levels, and the difficulties to observe the cell boundaries. Our inference of the cells in which Hoxaas3 is expressed is thus based on the overall evidence coming from the scRNA-seq data, the marker staining and the relative positions of the cells in which we see expression by smFISH. We have rephrased the text accordingly (Page 31):

"Based on the marker expression and the positions of the cells, we conclude that Hoxaas3 *is expressed in the early immature goblets and in the secretory precursor cells (**Fig. 6D**)."*

The reviewer is correct that there is a lot of background when imaging the intestine tissue. As controls, we imaged a channel in which no probe was added. In the analysis, we focused on points in which the signal matched diffraction-limited spots as shown in the following image.

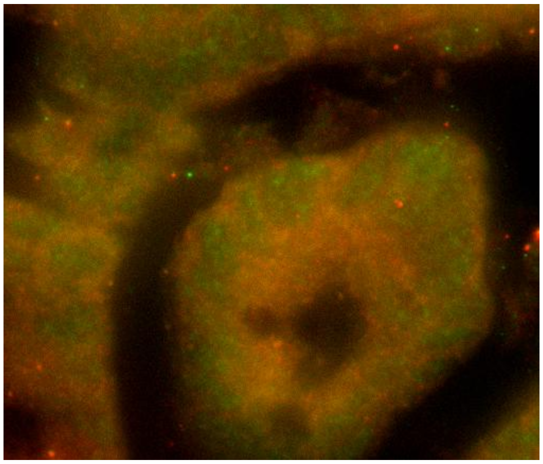

Reviewer Fig. 3 smFISH of *Hoxaas3* (red), *Hoxa5* (green) and no probe control (yellow) expression in the mouse intestine.

The ESC differentiation section was an excellent addition, strongly suggest to comprehensively characterise Hox expression in this system.

We now show the expression levels of all the Hox protein-coding genes and lncRNAs in the dataset from Loh et al. in **Fig S8A.** For characterization of the effects of HOXA/B-AS3 LOF/GOF on all the Hox expression, we prefer to focus on a more “static” system of HT-29, as changes in Hox expression can affect the pace of differentiation, which makes it difficult to discern between direct and indirect effects on the Hox network.

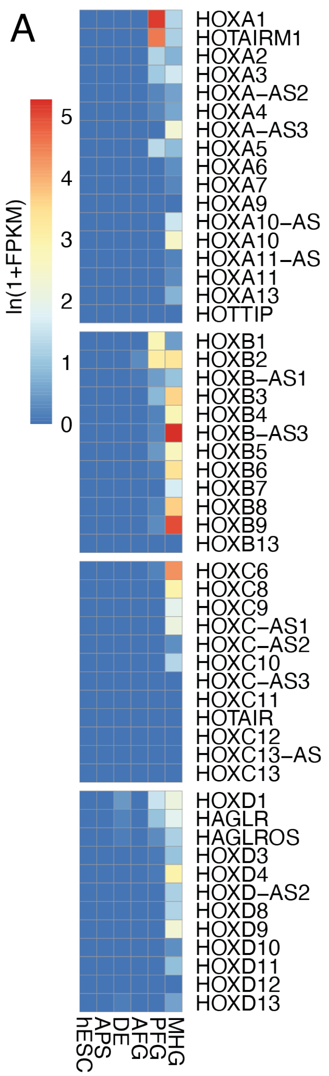

Figure S8A Expression levels of Hox genes and lncRNAs in data from (Loh et al. 2014)**.**

The Cdx1/2 direct regulation of HoxAS transcripts is interesting but of course preliminary. I find it difficult to interpret FigS8 (B) – siCdx1 results in expected knockdown of Cdx1, but siCdx2 also does, and when both siRNAs used the level of knockdown is less than either alone? Similar questions with other graphs. The benefit of its inclusion in general, and moreover, without directly supportive ChIPseq data is questionable.

We agree with the reviewer that these results are preliminary and have therefore removed this part from the manuscript.

Very minor text points

Abstract

“Sequence-similar homologs of both lncRNAs are found in multiple vertebrate species.” As mentioned above, I believe this is meant to be orthologs, and second, I believe you have compared

the promoter sequence but have you compared the lncRNA sequence similarity to support this statement?

We now call these orthologs, and as described above, perform analysis of lncRNA sequence similarity.

Pg 2:

“the molecular pathways that dictate their collinear expression remain mostly unknown.” Not sure this is strictly true, there’s increasing work in both ESCs and in vivo showing the signals and mechanisms that guide correct temporal Hox activation. This is of course not the point of this ms, but I would just slightly reword.

We rephrased this statement to: *“the molecular pathways that dictate their collinear expression are not fully understood.”*

Pg2:

“miR-196 (iab-4 in D. melanogaster)”

these microRNAs show functional conservation, but they are actually not conserved in sequence, nor located at the exact syntenic position, so are not related.

We removed the reference to iab-4, to avoid confusion.

References

Chan, Michelle M., Zachary D. Smith, Stefanie Grosswendt, Helene Kretzmer, Thomas M. Norman, Britt Adamson, Marco Jost, et al. 2019. “Molecular Recording of Mammalian Embryogenesis.” *Nature* 570 (7759): 77–82.

Consortium, Fantom, Riken Pmi The, Clst, A. R. Forrest, H. Kawaji, M. Rehli, J. K. Baillie, et al. 2014. “A Promoter-Level Mammalian Expression Atlas.” *Nature* 507 (7493): 462–70.

Khan, Aziz, Oriol Fornes, Arnaud Stigliani, Marius Gheorghe, Jaime A. Castro-Mondragon, Robin van der Lee, Adrien Bessy, et al. 2018. “JASPAR 2018: Update of the Open-Access Database of Transcription Factor Binding Profiles and Its Web Framework.” *Nucleic Acids Research* 46 (D1): D1284.

Kwon, Gloria S., Manuel Viotti, and Anna-Katerina Hadjantonakis. 2008. “The Endoderm of the Mouse Embryo Arises by Dynamic Widespread Intercalation of Embryonic and Extraembryonic Lineages.” *Developmental Cell* 15 (4): 509–20.

Loh, Kyle M., Lay Teng Ang, Jingyao Zhang, Vibhor Kumar, Jasmin Ang, Jun Qiang Auyeong, Kian Leong Lee, et al. 2014. “Efficient Endoderm Induction from Human Pluripotent Stem Cells by Logically Directing Signals Controlling Lineage Bifurcations.” *Cell Stem Cell* 14 (2): 237–52.

Nowotschin, Sonja, Manu Setty, Ying-Yi Kuo, Vincent Liu, Vidur Garg, Roshan Sharma, Claire S. Simon, et al. 2019. “The Emergent Landscape of the Mouse Gut Endoderm at Single-Cell Resolution.” *Nature* 569 (7756): 361–67.

Peng, Guangdun, Shengbao Suo, Guizhong Cui, Fang Yu, Ran Wang, Jun Chen, Shirui Chen, et al. 2019. “Molecular Architecture of Lineage Allocation and Tissue Organization in Early Mouse Embryo.” *Nature* 572 (7770): 528–32.

Ross, Caroline Jane, Aviv Rom, Amit Spinrad, Dikla Gelbard-Solodkin, Neta Degani, and Igor Ulitsky. 2021. “Uncovering Deeply Conserved Motif Combinations in Rapidly Evolving Noncoding Sequences.” *Genome Biology* 22 (1): 29.
