# Supplementary material for: Highly conserved and cis-acting lncRNAs produced from paralogous regions in the center of HOXA and HOXB clusters in the endoderm lineage
Source: PLoS Genet. 2021 Jul 19;17(7):e1009681. doi: 10.1371/journal.pgen.1009681 (PMC8330917; doi:10.1371/journal.pgen.1009681)
Supplement: Attachment — Submitted filename: Point-by-point HOXA_B-AS3 manuscript, round 2.pdf [file pgen.1009681.s017.pdf]

Point-by-point response to referees for “Highly conserved and *cis*-acting lncRNAs produced from paralogous regions in the center of HOXA and HOXB clusters in the endoderm lineage” by Degani et al.

Reviewer #4: The manuscript by Degani and co-authors presents an evolutionary and functional analysis of two long non-coding RNAs, transcribed on the antisense strand in the HOXA and HOXB clusters. The authors show that these lncRNAs are highly conserved during evolution and that their origin likely predates the whole-genome duplications that led to the emergence of the HOX gene clusters. They perform computational analyses of publicly available gene expression resources (including both bulk tissue expression and single cell RNA-seq data), as well as their own expression experiments to determine the spatial and temporal expression patterns of these two lncRNAs and of the neighboring HOXA and HOXB genes. Moreover, they perform in vitro knockdown (with CRISPRi and siRNA) and activation experiments for these two lncRNAs and they observe an up-regulation of the neighboring HOXA5-7 and HOXB5-7 genes. They conclude that these lncRNAs contribute to the complex regulatory mechanisms that control HOXA gene expression.

I find the evolutionary analyses convincing; there is no doubt that these lncRNAs are ancient and that they share similar structures (though not necessarily sequence conservation) in vertebrates.

I am less convinced by the loss-of-function and gain-of-function experiments and by the associated transcriptomics analyses. Here are my main comments:

1) As the authors themselves acknowledge in this manuscript, the transcriptional organization of the HOX clusters is very complex. There are many alternative isoforms, on the sense or antisense strand, not to mention numerous regulatory elements embedded in the locus. The molecular consequences of gene editing in these loci are thus hard to predict, and have to be carefully analyzed before drawing a definitive conclusion. I thus wonder what exactly happens with the transcriptional organization of the locus in the CRISPRi and CRISPRa experiments. Are the preferred transcript start sites identical? Albeit in a different experimental setting (a targeted genomic deletion of the HOTAIR lncRNA), it was shown that the HOTAIR alteration leads to the emergence of a new lncRNA at the locus and potentially to transcriptional leakage on neighboring HOX genes (Amandio et al, PLoS Genetics, 2016). As unfortunately we still know little of the molecular consequences of CRISPRi and CRISPRa experiments, it is worth examining the resulting transcript organization with RNA-seq assays rather than examining a few target genes with qRT-PCR.

We agree with the reviewer that consequences of genome editing within the HOX clusters are difficult to predict and interpret, and hence we focused on transient perturbations, including CRISPRi, CRISPRa, siRNAs, and shRNAs, in two experimental systems, HT29 and hESCs. We agree that a more detailed characterization of the cells perturbed with CRISPRi and CRISPRa, with various methods including RNA-seq, ATAC-seq etc. can shed further light on the details of the consequences on the loss of *HOXA-AS3* and *HOXB-AS3* on the complex loci their overlap and control, but we argue that this analysis is beyond the scope of the current study. Here we focused on the overall expression levels of the adjacent genes that we characterize with multiple systems and multiple perturbation methods. We performed, on a transcriptome-wide analysis, that is now expanded (see below), on siRNA treatments. We found that siRNAs have the best transfection efficiency in HT-29 cells, and the consequences of siRNAs are easier to interpret, as they are

considered to have a minimal effect on the chromatin and to predominantly target the RNA products of the lncRNAs. We now discuss the importance of future studies of the consequences of the perturbation methods on the different RNA isoforms produced from the HOXA and HOXB clusters (Page 10):
"It is of particular interest to study whether HOXA-AS3 and HOXB-AS3 influence the nature of the transcripts produced in the complex loci of the HOX clusters, e.g., but influencing promoter choice."

2) The authors do present an RNA-seq analysis for the siRNA knockdown performed on HT-29 cells (S2 table, qRT-PCR experiments in Figure 4). However, the differential expression analysis is not convincing: unless I have misunderstood the table (a detailed legend would help), there is no significant difference in expression for any HOX genes upon siRNA down-regulation of HOXA-AS3 and HOXB-AS3. In fact even the HOXA-AS3 and HOXB-AS3 transcripts do not show any significant expression change - I am not sure if this is because the expression levels are too low or too variable. This RNA-seq analysis (for the HOXA6-7 and HOXB6-7 genes) is not consistent with the qRT-PCR analysis shown in Figure 4. In general, qRT-PCR analyses, which show only fold changes but do not inform on the basal expression levels of the focus genes, ares not sufficient to prove that there is an effect on the target gene expression.

In the previous version of the manuscript, we only performed two biological replicates of the siRNA experiment followed by RNA-seq, which limited our power in detecting differential expression. We now performed and sequenced three additional biological replicates, and the changes in the expression of HOXA-AS3 and HOXB-AS3 in their respective perturbed cells are significant with adjusted $P<0.05$, as are changes in additional genes in the clusters, marked in Fig. 6C. We now emphasize that the changes observed in the HOXB clusters are generally more significant likely due to the higher expression levels of the HOXB genes in HT-29 cells (Page 6):
*"RNAi resulted in reduction in expression of the lncRNAs, concomitantly with reduction in the overlapping genes, and a broad mild reduction in expression of genes in the HOXA and HOXB clusters (HOXC and HOXD clusters are mostly silent in HT-29 cells) (**Fig. 4D** and **S2 Table**), with more significant effects observed in the HOXB cluster that is overall more expressed than HOXA in HT-29 cells (**S7A Fig.**)."*

Overall, I think it is important to emphasize that all the experiments performed in the manuscript are performed in vitro, often on cancer cell lines that likely have numerous chromosomal alterations, and may not adequately reflect the situation in vivo. I would thus recommend that the results be interpreted with extreme caution.

We agree with the reviewer and now mention this in the Discussion section:
"We report a positive effect of HOXA-AS3 and HOXB-AS3 production on the expression levels of their overlapping HOX5–7 genes. We studied these effects in vitro in cultured cells and mostly in a cancer cell line with an abnormal karyotype, and future studies will elucidate the roles of HOXA-AS3 and HOXB-AS3 RNA products in vivo."
